# Discovery of Quinazolone Pyridiniums as Potential Broad-Spectrum Antibacterial Agents

**DOI:** 10.3390/molecules30020243

**Published:** 2025-01-09

**Authors:** Jie Dai, Qianyue Li, Ziyi Li, Zhonglin Zang, Yan Luo, Chenghe Zhou

**Affiliations:** 1 Institute of Bioorganic & Medicinal Chemistry, Key Laboratory of Applied Chemistry of Chongqing Municipality, School of Chemistry and Chemical Engineering, Southwest University, Chongqing 400715, China; 2 College of Pharmacy, National & Local Joint Engineering Research Center of Targeted and Innovative Therapeutics, Chongqing Key Laboratory of Kinase Modulators as Innovative Medicine, Chongqing University of Arts and Sciences, Chongqing 402160, China

**Keywords:** quinazolone, pyridinium, resistance, antibacterial, PBP2a

## Abstract

The overprescription of antibiotics in medicine and agriculture has accelerated the development and spread of antibiotic resistance in bacteria, which severely limits the arsenal available to clinicians for treating bacterial infections. This work discovered a new class of heteroarylcyanovinyl quinazolones and quinazolone pyridiniums to surmount the increasingly severe bacterial resistance. Bioactive assays manifested that the highly active compound **19a** exhibited strong inhibition against MRSA and *Escherichia coli* with extremely low MICs of 0.5 μg/mL, being eightfold more active than that of norfloxacin (MICs = 4 μg/mL). The highly active **19a** with rapid bactericidal properties displayed imperceptible resistance development trends, negligible hemolytic toxicity, and effective biofilm inhibitory effects. Preliminary explorations on antibacterial mechanisms revealed that compound **19a** could cause membrane damage, embed in intracellular DNA to hinder bacterial DNA replication, and induce metabolic dysfunction. Surprisingly, active **19a** was found to trigger the conformational change in PBP2a of MRSA to open the active site, which might account for its high inhibition against MRSA. In addition, the little effect of molecule **19a** on the production of reactive oxygen species indicated that bacterial death was not caused by oxidative stress. The above comprehensive analyses highlighted the large potential of quinazolone pyridiniums as multitargeting broad-spectrum antibacterial agents.

## 1. Introduction

The advent of antibiotics in the 20th century marked a monumental leap in medical science, offering humanity a powerful weapon against bacterial infections. This discovery, alongside the subsequent development of various synthetic antibacterial agents, significantly reduced the morbidity and mortality associated with infectious diseases [1,2]. However, the overuse and misuse of antimicrobials have precipitated a decline in the effectiveness of traditional antibiotics, and exacerbated the emergence of cross-resistance among different classes of antibiotics, which poses a formidable challenge against microbial infections [3,4]. Hence, there is an urgent need to identify new classes of antibacterial drugs with novel structures to conquer infections caused by multidrug-resistant bacteria.

Quinazolone is a crucial structural scaffold widely present in some traditional Chinese herbal medicinal ingredients such as dichroine, evodiamine, and tryptanthrine. This unique scaffold with easy modification has been extensively employed in medicinal design, and a large number of quinazolone-based drugs such as idelalisib, metolazone, afloqualone, and so on have achieved remarkable successes in clinical practice to treat various diseases [5,6,7]. Particularly, quinazolone is structurally similar to benzopyridone, which is the core skeleton of the marketed antibacterial quinolones and is frequently employed as an attractive structural framework in the field of antibacterial aspects [8,9,10]. Much work has revealed the great potential of quinazolone derivatives in combating bacterial resistance through multitargeting antibacterial mechanisms [11,12,13]. One possible reason might be assigned to its large aromatic and rigid skeleton, which can helpfully intercalate into DNA by π-π stacking to form a supramolecular complex and thus interfere with the normal physiological function of DNA. Another reasonable possibility might be that some quinazolones have been proposed as promising PBP2a allosteric inhibitors, exhibiting excellent antibacterial activity [14,15,16]. The above findings stimulated our great enthusiasm to develop novel antibacterial quinazolone derivatives with multitargeting potential to overcome the growing bacterial resistance.

Aromatic heterocycles such as five membered heterocycles like thiazoles [17,18] and imidazoles [19,20], as well as six membered ones like pyrimidines [21,22] and pyridines [23], have played important roles in the antibacterial field, and many heterocyclic derivatives as antibacterial drugs have been marketed [24]. For instance, the incorporation of thiazole fragments in the third and fourth generations of cephalosporin drugs, as well as the inclusion of imidazole fragments in medications like ornidazole and metronidazole, implied their large potential in antibacterial aspects. Their unique structures could easily bind with various biological targets through noncovalent interactions by use of an aromatic skeleton and heterocycles, thus their incorporation might helpfully improve affinity and bioactivity [25,26]. Inspired by these achievements, various types of five- and six-membered aromatic heterocycles as well as their benzene-fused analogs were coupled with quinazolone with the expectation to identify novel potential antibacterial candidates that exhibit broad-spectrum, high-efficiency, and low-toxicity characteristics.

The cyanovinyl fragment with both cyano and vinyl active groups is an important functional group in chemical reactions, garnering special attention due to its structural similarity to ethenones. It is widely present in many drugs and candidates with various biological activities, including antimicrobial drugs like luliconazole and lanoconazole. Furthermore, the introduction of the cyanovinyl group could not only beneficially bind with biotargets via noncovalent interactions, but also helpfully improve the pharmacokinetic properties of small-molecule drugs and can be metabolized unchangeably by organisms without the release of cyanide [27,28]. Therefore, this work delves into the potential of quinazolones linked with different heterocycles through cyanovinyl linker with the aim of exploring their potential to combat microbial infections.

Quaternary ammonium compounds play a positive role in antibacterial drugs such as fourth-generation cephalosporins like antibacterial cefquinome and cefpirome [29,30,31]. It is well-known that pyridine is easy to react with some alkyl and benzyl halides to give stable quaternary ammonium salts. The resultant pyridiniums are capable of binding with the anionic groups of phospholipids in the membrane by the use of positively charged quaternary nitrogen, thus disrupting the structure of bacterial cell membranes, altering their permeability, and finally improving antibacterial performance [32,33]. Therefore, different substituted pyridiniums were prepared to investigate the effect on activity.

**Figure 1:**
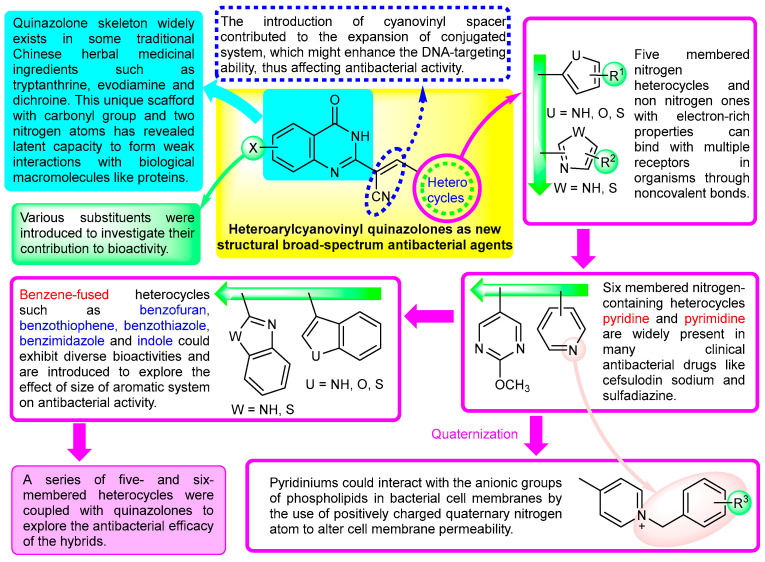
Design of heteroarylcyanovinyl quinazolones and quinazolone pyridiniums as potential antibacterial agents.

Given the above considerations, a series of novel structural heteroarylcyanovinyl quinazolones and quinazolone pyridiniums were prepared (Figure 1), and their antibacterial efficacy in vitro was tested. A highly active compound was selected to further investigate its druggabilities, including hemolytic effects, the development of resistance, bactericidal kinetics, and anti-biofilm activity. Moreover, the preliminary antibacterial mechanism for the highly active compound was explored by various experiments that were mainly concerned with the damage of membrane, leakage of intracellular protein, generation of reactive oxygen species, interaction with DNA, and allosteric modulation experiments of PBP2a.

## 2. Results and Discussion

### 2.1. Chemistry

**Scheme 1:**
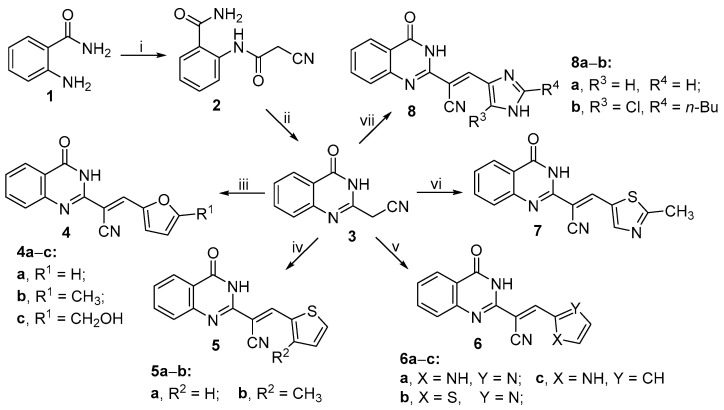
Preparation of the target products **3**–**7**. Reagents and conditions: (i) cyanoacetic acid, 1-(3-dimethylaminopropyl)-3-ethylcarbodiimide hydrochloride, 1-hydroxybenzotriazole hydrate, *N*,*N*-dimethyl formamide, r.t.; (ii) 10% sodium hydroxide aqueous solution, r.t.; (iii) furan aldehydes, piperidine, ethanol, 80 °C; (iv) thiophene aldehydes, piperidine, ethanol, 80 °C; (v) imidazole-2-carboxaldehyde or thiazole-2-carboxaldehyde or 2-pyrrolecarbaldehyde, piperidine, ethanol, 80 °C; (vi) 2-methylthiazole-5-carbaldehyde, piperidine, ethanol, 80 °C; (vii) imidazole-4-carboxaldehydes, piperidine, ethanol, 80 °C.

**Scheme 2:**
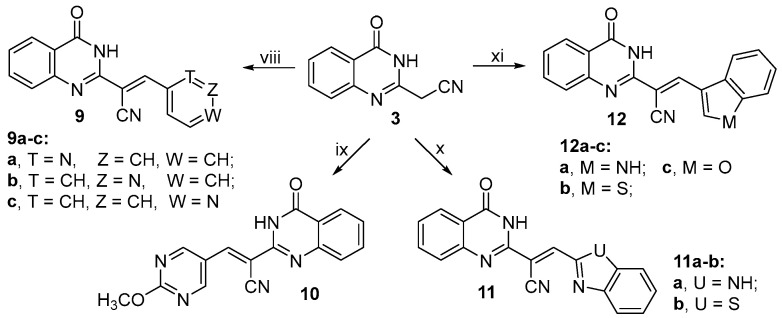
Preparation of the target products **9**–**12**. Reagents and conditions: (viii) pyridine aldehyde, piperidine, ethanol, 80 °C; (ix) 2-methoxy-5-pyrimidine aldehyde, piperidine, ethanol, 80 °C; (x) benzimidazole-2-formaldehyde or benzothiazole-2-formaldehyde, piperidine, ethanol, 80 °C; (xi) indole-3-carboxaldehyde or benzothiophene-3-carbaldehyde or benzofuran-3-carbaldehyde, piperidine, ethanol, 80 °C.

**Scheme 3:**
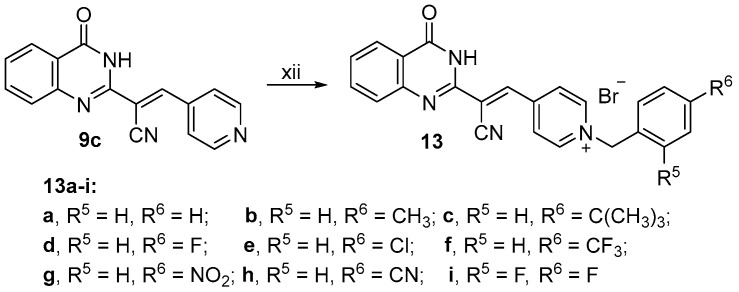
Preparation of quinazolone pyridiniums **13a**–**i**. Reagents and conditions: (xii) benzyl bromides, potassium carbonate, acetonitrile, 80 °C.

**Scheme 4:**
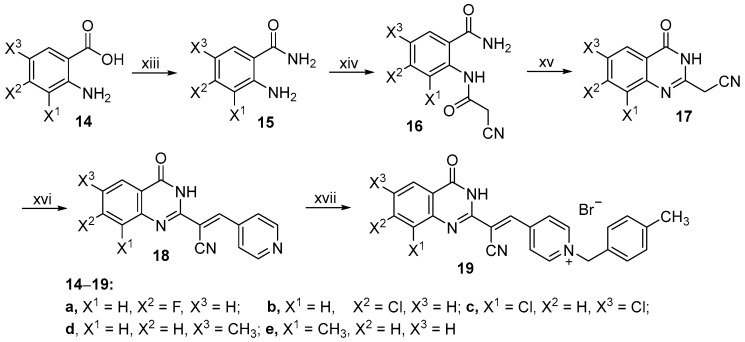
Preparation of quinazolone pyridiniums **19a**–**d** with different substituents on quinazolone ring. Reagents and conditions: (xiii) *N*,*N*-carbonyldiimidazole, 25% ammonia water solution, *N*,*N*-dimethyl formamide, 70 °C; (xiv) cyanoacetic acid, 1-(3-dimethylaminopropyl)-3-ethylcarbodiimide hydrochloride, 1-hydroxybenzotriazole hydrate, *N*,*N*-dimethyl formamide, r.t.; (xv) 10% sodium hydroxide aqueous solution, r.t.; (xvi) 4-formylpyridine, piperidine, ethanol, 80 °C; (xvii) 4-methylbenzyl bromide, potassium carbonate, acetonitrile, 80 °C.

The desired heteroarylcyanovinyl quinazolones and quinazolone pyridiniums were synthesized according to Scheme 1, Scheme 2, Scheme 3 and Scheme 4. The amide intermediate **2** was prepared in 50.3% yield by the reaction of commercially purchased *o*-aminobenzamide with cyanoacetic acid in the presence of condensing agents 1-(3-dimethylaminopropyl)-3-ethylcarbodiimide hydrochloride and 1-hydroxybenzotriazole hydrate at room temperature, and further cyclization in a 10% sodium hydroxide aqueous solution produced the important precursor **3** with a high yield of 88.2%. Cyanomethylquinazolone **3** was condensed with different five-membered heteroaromatic aldehydes in the presence of piperidine as a catalyst and ethanol as a solvent to afford the target products **4**–**8** with yields ranging from 49.2–93.2% (Scheme 1). To explore the effect of the larger conjugated system on antibacterial activity, six-membered pyridine and pyrimidine aldehydes and some benzene-fused heterocyclic aldehydes were employed to prepare the target products **9**–**12** with yields ranging from 32.4–94.7% under the same reaction conditions (Scheme 2).

Preliminary antibacterial assay indicated that 4-pyridine derivative **9c** exhibited good anti-*Escherichia coli* activity, thus compound **9c** was further optimized to react with different benzyl bromides to produce quinazolone pyridiniums **13a**–**i** (Scheme 3) because it has been revealed that quaternary ammonium compounds are able to not only disrupt the structure of bacterial cell membranes and alter their permeability but also improve their antibacterial activity.

Finally, the obtained highly active compound **13b** was further optimized by changing the substituents in the quinazolone ring such as introducing fluorine, chlorine, and methyl groups. A series of intermediates **15a**–**e** were prepared in acetonitrile at 70 °C by the reaction of corresponding aminobenzoic acids **14a**–**e** and 25% ammonia water solution, using *N*,*N’*-carbonyldiimidazole as catalyst. The target molecules **19a**–**e** were prepared according to the procedures for preparation of quinazolone pyridinium **13b** (Scheme 4).

### 2.2. Antibacterial Activity

The in vitro antibacterial activities of all the synthesized compounds were evaluated by the continuous dilution method recommended by Clinical and Laboratory Standards Institute (CLSI), and antibacterial norfloxacin was used as the positive control [34]. As shown in Table 1, most of the prepared compounds exhibited moderate to good antibacterial activity and some of them showed equipotent antibacterial activity to norfloxacin for some tested bacteria, indicating that heteroarylcyanovinyl quinazolones were potential to confront infectious bacteria.

Furyl derivatives **4a**–**c** demonstrated good antibacterial effects on *E. coli*, with MIC values ranging from 2 to 4 μg/mL, comparable to norfloxacin. Methyl-substituted compound **4b** gave better antibacterial activity than unsubstituted **4a** against *E. faecalis*, *E. coli* ATCC 25922, and *P. aeruginosa*, highlighting the beneficial role of methyl substitution. However, the hydroxymethyl derivative **4c** was unfavorable for enhancing activity. Thiophene hybrids **5a**–**b** also showed good inhibitory activity against most of the tested strains, and unmodified thienyl derivative **5a** exhibited better bacteriostatic effect against *S. aureus* ATCC 25923 and *P. aeruginosa* (MICs = 1 μg/mL), which was fourfold more active than norfloxacin. However, the incorporation of methyl group into thienyl ring was negative because compound **5b** gave lower activity in comparison to the unsubstituted **5a**. Among compounds **6**–**8** with other five-membered heterocycles, thiazolyl derivative **6b** effectively suppressed the growth of *E. coli* and *P. aeruginosa*, with MICs of 1 μg/mL. The pyrrolyl derivative **6c** gave a specific inhibition on *S. aureus* ATCC 25923, which was fourfold more potent than norfloxacin.

Noticeably, pyridyl derivatives **9a**–**c** also exerted good antibacterial inhibition against Gram-positive bacteria; particularly, 4-pyridyl derivative **9c** exhibited strong antibacterial potency against *S. aureus* ATCC 29213, *E. coli,* and *P. aeruginosa* with low MICs of 1 μg/mL, which was two-, four-, and fourfold more active than norfloxacin, respectively. It is noteworthy that 3-pyridyl analog **9b** gave good anti-MRSA performance with an MIC value of 2 μg/mL, which was superior to norfloxacin (MIC = 4 μg/mL). However, the pyrimidyl analog **10** displayed moderate antibacterial ability against most of the tested bacteria, but it demonstrated twofold inhibitory effect in contrast to norfloxacin against *S. aureus* ATCC 25923 and *P. aeruginosa*.

To investigate the effect of larger conjugated heterocycles on antibacterial activity, the five-membered heteroaryl moieties were replaced by benzimidazolyl and benzotriazolyl rings. Unfortunately, this replacement was unfavorable for enhancing the antibacterial activity, and compounds **11a** and **11b** showed some inhibition only for individual strains.

Quaternary ammonium compounds are widely present in antibacterial cephalosporins such as cefozopran and ceftaroline, in which organic oniums can not only destroy bacterial cell membranes but also enhance antibacterial potency. Therefore, compound **9c** was further transformed into the corresponding pyridiniums **13a**–**i** by introducing different substituted benzyl bromides. In comparison to compound **9c**, its pyridiniums **13a**–**i** gave better antibacterial performance. Particularly, *p*-methylbenzyl pyridinium **13b** showed outstanding antibacterial efficacy against MRSA and *E. coli*, with low MIC values of 1 and 0.5 μg/mL, respectively, which was four- and eightfold more active than norfloxacin. This result indicated that the introduction of pyridinium moiety was positive for antibacterial potential.

In view of the good antibacterial potential of *p*-methylbenzyl pyridinium **13b**, this encouraged us to further investigate the effect of different substituents in the quinazolone ring on antibacterial activity of target pyridiniums **19a**–**e**. Antibacterial evaluation indicated that 7-fluoroquinazolone pyridinium **19a** gave exceptional activity against MRSA and *E. coli*, with MIC values of 0.5 μg/mL, which were eightfold more potent than norfloxacin. Methyl substitution at *C*-6 position of quinazolone exerted good anti-*P. aeruginosa* potency; whereas the introduction of chorine at *C*-7 position or both *C*-6 and *C*-8 was unfavorable for bioactivity. Considering that quinazolone pyridinium **19a** displayed the most broad-spectrum antibacterial activity against all the tested strains (MIC values = 0.5–8 μg/mL), this compound was selected for further investigation on its druggability and preliminary antibacterial mechanisms.

### 2.3. Hemolytic Assay

**Figure 2:**
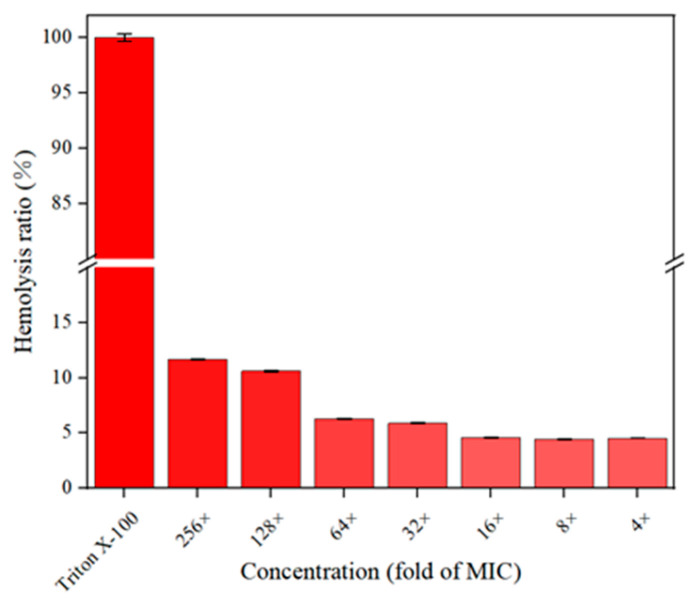
Assessment of hemolysis of quinazolone pyridinium **19a**.

The hemolysis test is an indispensable evaluation criterion in drug research and clinical application. Healthy human red blood cells were treated by varying concentrations of compound **19a** for 3 h, using saline and 1% Triton X-100 solution as negative and positive controls, respectively, and the percentage of red blood cell lysis was measured [35,36]. As depicted in Figure 2 and Figure S1, compound **19a** exhibited a low hemolysis rate at a high concentration of 256 × MIC. At the concentration of 16-fold MIC, the hemolytic activity of quinazolone pyridinium **19a** (4.57%) was below the highest international standard hemolytic rate of 5%, indicating the low toxicity of compound **19a** to human blood cells.

### 2.4. Resistance Study

**Figure 3:**
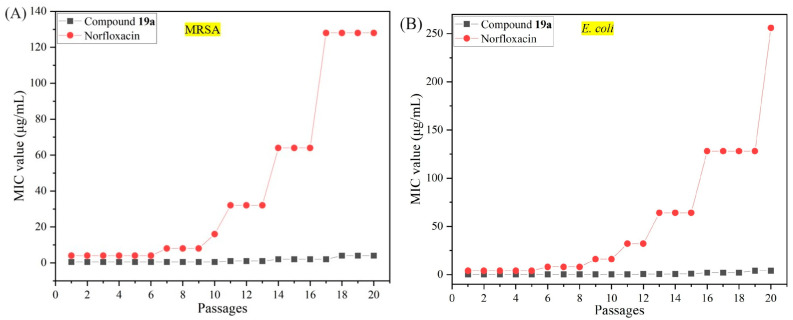
Resistance development of quinazolone pyridinium **19a** and norfloxacin against MRSA (**A**) and *E. coli* (**B**).

The emergence of bacterial resistance leads to a decrease in clinical drug efficacy or treatment failure, greatly increasing the difficulty of clinical treatment [37,38]. The trend of resistance development of quinazolone pyridinium **19a** against MRSA and *E. coli* was investigated, with norfloxacin as positive control [39,40]. After 20 generations of cultivation, both MRSA and *E. coli* exhibited significant resistance to norfloxacin, with MIC values increasing by 32- and 64-fold, respectively. In contrast, compound **19a** hardly developed resistance against both bacteria (Figure 3). These outcomes suggested that the occurrence of resistance was difficult under the treatment of compound **19a**, implying the large potential of the highly active molecule **19a** as an antibacterial candidate.

### 2.5. Bactericidal Kinetics

**Figure 4:**
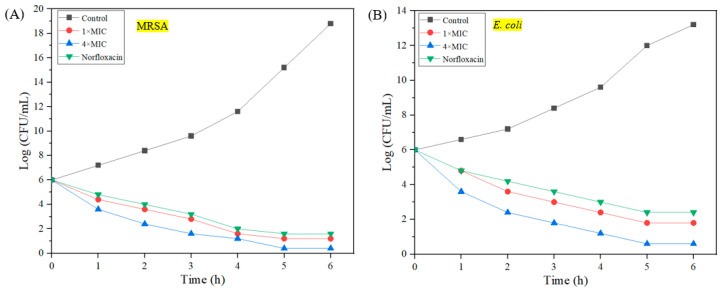
Bactericidal kinetic curves of quinazolone pyridinium **19a** and norfloxacin against MRSA (**A**) and *E. coli* (**B**).

The rapid bactericidal capability is helpful to reduce the duration of bacterial infections and thus lower the probability of resistance emergence. To further explore the efficiency of compound **19a** in killing bacterial cells, the bactericidal kinetics of quinazolone pyridinium **19a** and norfloxacin against MRSA and *E. coli* were evaluated [41,42,43]. As illustrated in Figure 4, compound **19a** was able to rapidly eliminate MRSA and *E. coli* within 6 h, displaying superior bactericidal effects to the reference drugs. Nearly complete bacterial eradication was achieved after 6 h of cultivation at a concentration of 4 × MIC. These results revealed that quinazolone pyridinium **19a** could quickly kill bacteria, which might contribute to preventing the development of drug resistance.

### 2.6. Antibiofilm Activity

**Figure 5:**
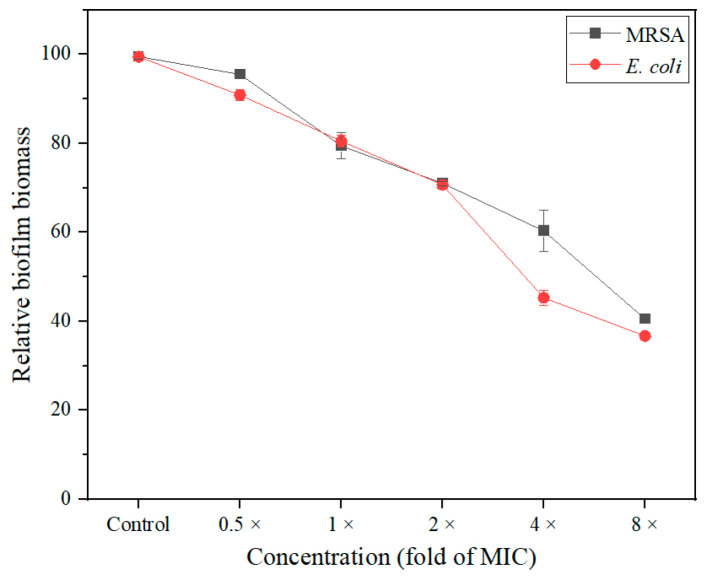
The biofilm inhibition of quinazolone pyridinium **19a** against MRSA and *E. coli*.

Bacterial biofilms composed of bacteria and their own extracellular polymers can protect bacteria from the killing of antibiotics and the impact of host immune response, increasing their resistance to antibiotics by 1000 times, resulting in wounds that are difficult to heal for a long time [44,45]. Therefore, the drug efficacy of antibacterial agents will be severely affected once biofilms are formed. Crystal violet staining assay was conducted to investigate the inhibitory effect of quinazolone pyridinium **19a** on the formation of MRSA and *E. coli* biofilm [46,47]. As shown in Figure 5, compound **19a** could inhibit biofilm formations of both bacteria in a concentration-dependent manner. The inhibitory rate of MRSA and *E. coli* biofilms exceeded 60% at the concentration of eightfold MIC. These results indicated the excellent anti-biofilm capability of quinazolone pyridinium **19a** against MRSA and *E. coli*, potentially contributing to its low propensity for resistance development.

### 2.7. Membrane Damage Assay

The bacterial cell membrane with specific morphology protects the cell from external disturbances to ensure a relatively stable internal microenvironment [48,49]. Additionally, the cell membrane controls the transport of nutrients as a conduit for intracellular and extracellular substance exchange. Many signal protein receptors on the cell membrane facilitate internal and external signal transmission, making membrane stability crucial for bacterial growth [50,51]. In view of the large potential of organic oniums in penetrating cell membranes and altering membrane permeability, the membrane-targeting ability of quinazolone pyridinium **19a** was investigated.

#### 2.7.1. Membrane Depolarization Assay

**Figure 6:**
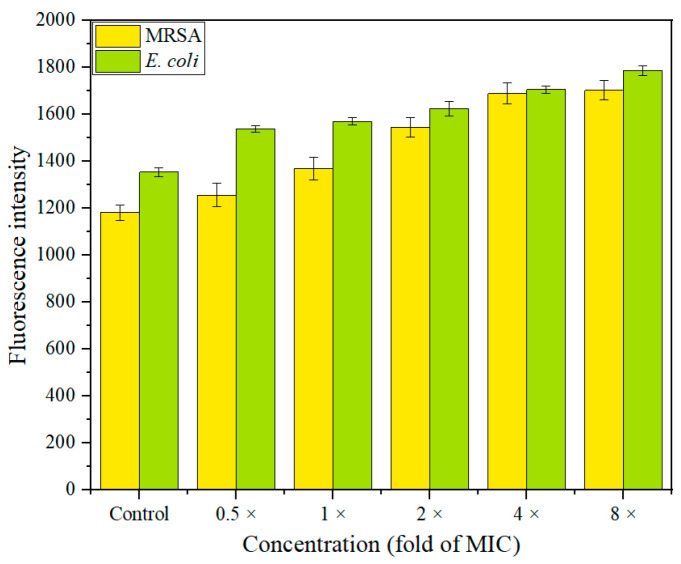
MRSA and *E. coli* cell membrane depolarization evoked by quinazolone pyridinium **19a**.

The depolarization of the cytoplasmic membrane can perturb the potential of the bacterial membrane, which disrupts the electrochemical balance and impairs normal physiological functions [52]. Using the 3,3′-dipropylthiadicarbocyanine iodide (diSC35) fluorescent indicator, the effect of quinazolone pyridinium **19a** on the membrane potential of MRSA and *E. coli* was assessed [53,54]. The results, shown in Figure 6, indicated that the fluorescence intensity in both bacteria increased with the concentration of compound **19a**, suggesting that it could depolarize the membrane in a concentration-dependent manner.

#### 2.7.2. Assay of Outer Membrane Damage

**Figure 7:**
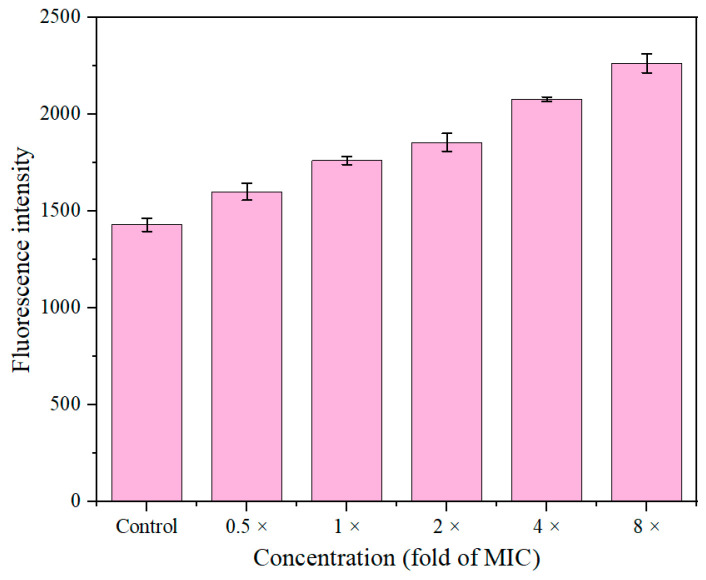
Outer membrane permeability of quinazolone pyridinium **19a** against *E. coli*.

Gram-negative bacteria with a rigid protective outer shell are more difficult to inactivate than Gram-positive bacteria. The Gram-negative cell membranes composed of outer and inner membranes are conducive to prevent antibiotics and other antibacterial agents from entering and destroying the cells [55,56]. The permeabilizing effect of quinazolone pyridinium **19a** on the outer membrane of *E. coli* was determined using hydrophobic 1-*N*-phenylnaphthylamine (NPN), a fluorescent indicator that emits fluorescence upon encountering membrane-internal hydrophobic substances [57,58]. As depicted in Figure 7, the fluorescence intensity positively correlated with the concentration of compound **19a**, indicating that quinazolone pyridinium **19a** could enhance the permeability of the *E. coli* outer membrane in a concentration-dependent manner, thereby compromising membrane integrity.

#### 2.7.3. Study of Inner Membrane Permeabilization

**Figure 8:**
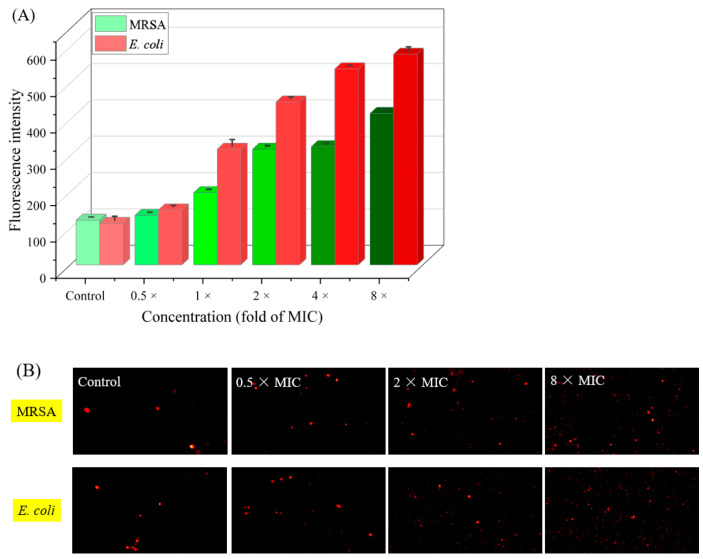
The permeabilization of inner membrane with increasing concentrations of quinazolone pyridinium **19a**. (**A**) Increased inner membrane permeability of MRSA and *E. coli* evoked by quinazolone pyridinium **19a**. (**B**) Fluorescence micrographs (400× magnification) of PI staining after treatment of MRSA and *E. coli* with quinazolone pyridinium **19a**.

Inner membrane permeability was further investigated toward MRSA and *E. coli* to explore the membrane disruption of quinazolone pyridinium **19a** using propidium iodide (PI), a living cell membrane impenetrable dye [59,60]. PI can only penetrate the cell membrane of dead bacteria to reach the nucleus, where it intercalates with the cell’s DNA to produce red fluorescence. The accumulation of red fluorescence within the cells of both MRSA and *E. coli* increased with the concentration of quinazolone pyridinium **19a**, manifesting the increasing contents of the PI in bacterial cell and the gradual death of strains. Microscopy images of bacteria stained with PI (Figure 8B) also gave stronger red fluorescence after treatment, which underscored the enhanced permeability of the inner membranes of MRSA and *E. coli* induced by compound **19a**.

Experiments on cell membrane depolarization and permeability revealed that quinazolone pyridinium **19a** effectively disrupted the membrane stability and integrity of MRSA and *E. coli*, potentially causing damage to the bacterial cell membrane, ultimately leading to bacterial apoptosis.

### 2.8. The Leakage of Intracellular Protein and Nucleic Acid

**Figure 9:**
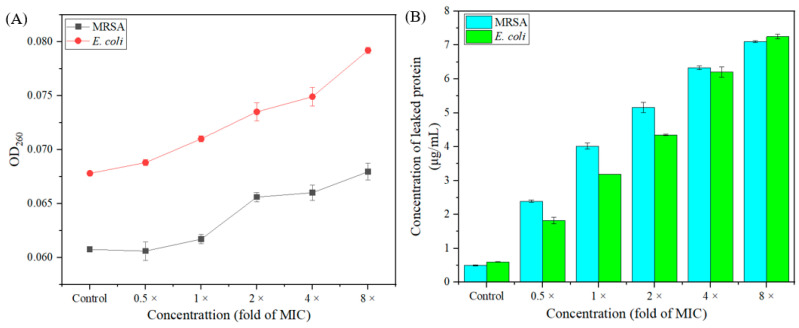
Nucleic acid (**A**) and protein (**B**) leakage induced by quinazolone pyridinium **19a**.

The biomacromolecules such as nucleic acids and protein inside the cell will have a chance to flow out once the structure of the cell membrane is destroyed [61]. This was evidenced by the measurement of nucleic acid leakage from MRSA and *E. coli* at 260 nm using a spectrophotometer, which showed an increase in leakage correlating with the concentration of **19a** (Figure 9). Similar results were obtained for protein leakage through the Bradford assay, and the fluorescence intensity in MRSA and *E. coli* treated by compound **19a** at 8 × MIC was 12- and 14-fold higher than that of the control group. These outcomes further supported a loss of cell membrane integrity caused by compound **19a**, consistent with its ability to depolarize bacterial membranes and enhance inner and outer membrane permeability.

### 2.9. Intracellular ROS Accumulation

**Figure 10:**
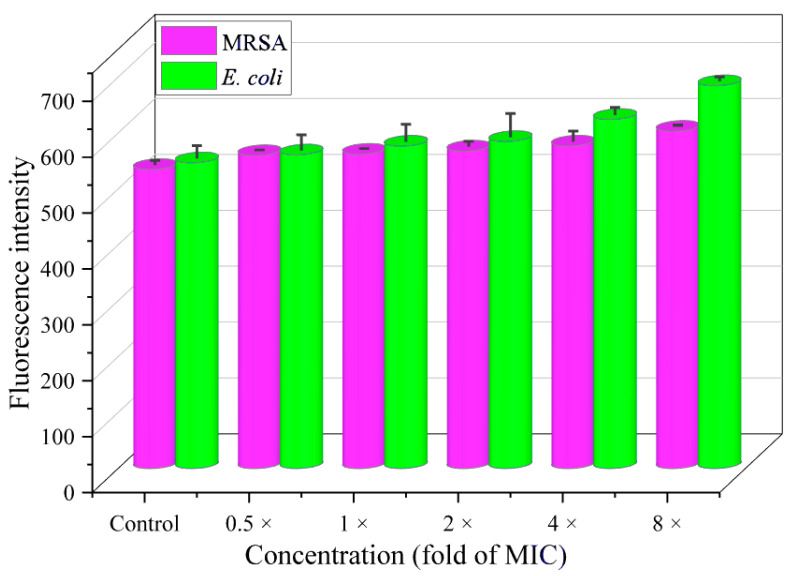
Enhancement of ROS levels in bacteria after treatment with quinazolone pyridinium **19a**.

Appropriate reactive oxygen species (ROS) plays an important role in bacterial signal transduction and homeostasis, while high levels of ROS may cause dysfunction, disease, and even cell death [62,63]. The aromatic conjugated system with a large skeleton is generally believed to stimulate the production of high concentrations of ROS, cause oxidative stress and serious damage to cell structure, and ultimately lead to bacterial apoptosis. Considering the large conjugated skeleton of quinazolone pyridinium **19a**, its effect on the generation of ROS in bacteria was assessed using the DCFH-DA probe [64,65]. As shown in Figure 10, a modest increase in fluorescence intensity indicated a slight stimulation of ROS production by compound **19a** in both MRSA and *E. coli*, suggesting that oxidative stress was not the dominant factor of bacterial death.

### 2.10. Detection of Metabolic Activity

**Figure 11:**
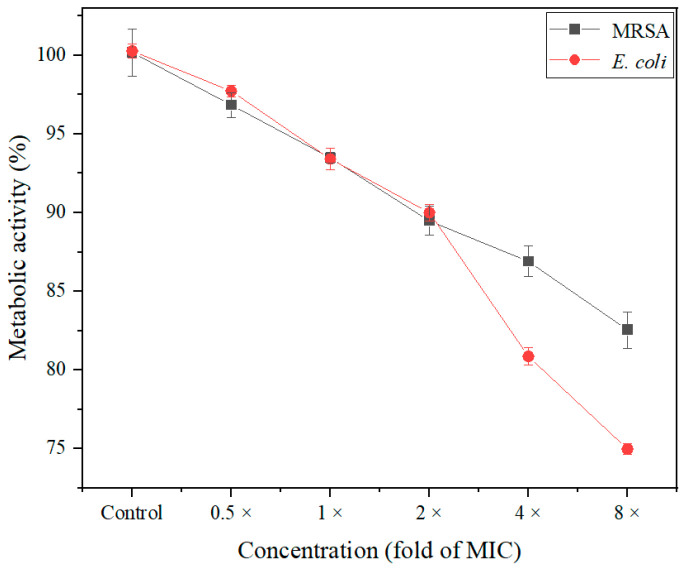
Decreased metabolic activity of bacteria after treatment with quinazolone pyridinium **19a**.

The change of metabolic activity in both bacteria types post-treatment with quinazolone pyridinium **19a** was determined using the redox indicator Alamar Blue [66,67]. As depicted in Figure 11, the metabolic activity of MRSA and *E. coli* decreased to varying degrees after treatment with compound **19a**, showing a negative correlation with the concentration of the compound. Notably, the metabolic activity of *E. coli* was more significantly affected than that of MRSA, which was consistent with the previous observations of inner membrane permeabilization. These results suggested that quinazolone pyridinium **19a** would cause bacterial membrane damage and further lead to metabolic inactivation.

### 2.11. Interaction Between Compound **19a** and DNA

DNA as an essential biomacromolecule for bacterial development and normal metabolism has become a significant therapeutic target in multitudinous bacterial infections [68]. The damage to bacterial cell membranes facilitates the entry of quinazolone pyridinium **19a** into the cells to bind with biological targets such as DNA, exerting its bioactivity. Therefore, the interaction between compound **19a** and calf thymus DNA was explored using UV-visible and fluorescence spectroscopy.

#### 2.11.1. DNA Binding Study

**Figure 12:**
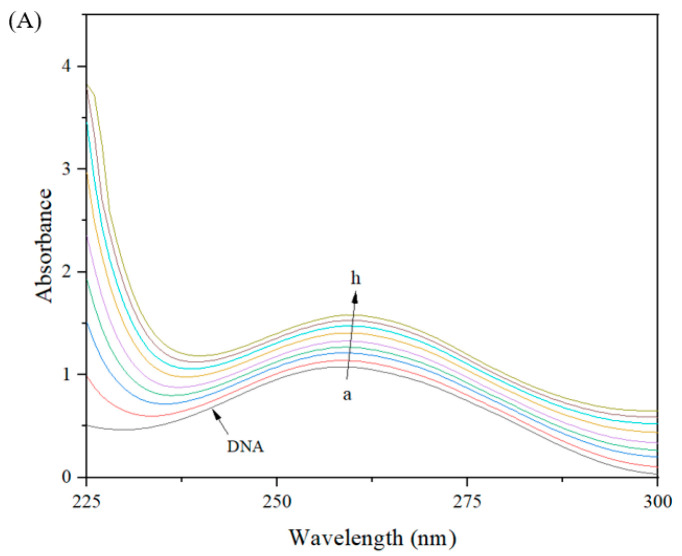

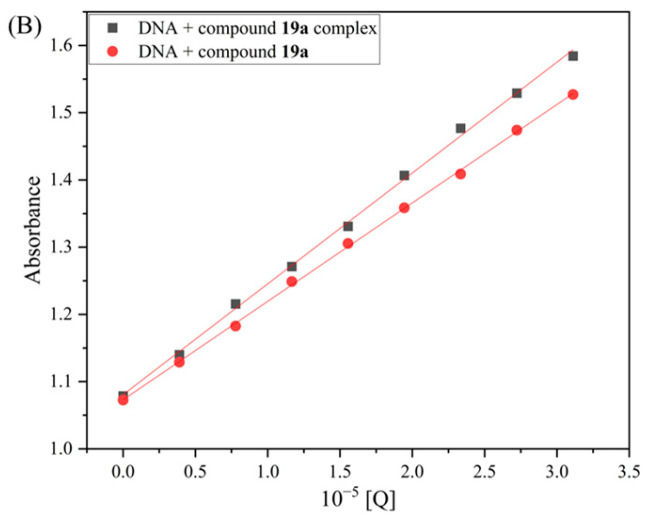
(**A**) Absorbance spectra of DNA with quinazolone pyridinium **19a**. *c*(DNA) = 7.41 × 10^−5^ mol/L, and *c*(compound **19a**) = 0−3.111 × 10^−5^ mol/L for curves a–h respectively at increment 0.4444 × 10^− 5^ mol/L. (**B**) Comparison of the absorption at 260 nm between the value of compound **19a**–DNA complex and the sum values of free DNA and free **19a**.

The binding ability of quinazolone pyridinium **19a** to calf thymus DNA was investigated by UV-vis spectroscopy [69,70]. As shown in Figure 12, the maximum absorption peak of DNA at 260 nm increased proportionally with the concentration of compound **19a**, accompanied by a slight red shift, potentially due to orbital overlap and coupling during the binding of compound **19a** with DNA, and the subsequent reduction in π-electron transition energy. Moreover, the absorption of the molecule **19a**-DNA complex exceeded the sum of their individual absorptions, indicating the presence of hyperchromic effect interaction. This might be attributed to the damage to the DNA double-helix structure.

#### 2.11.2. Competitive Binding Study

**Figure 13:**
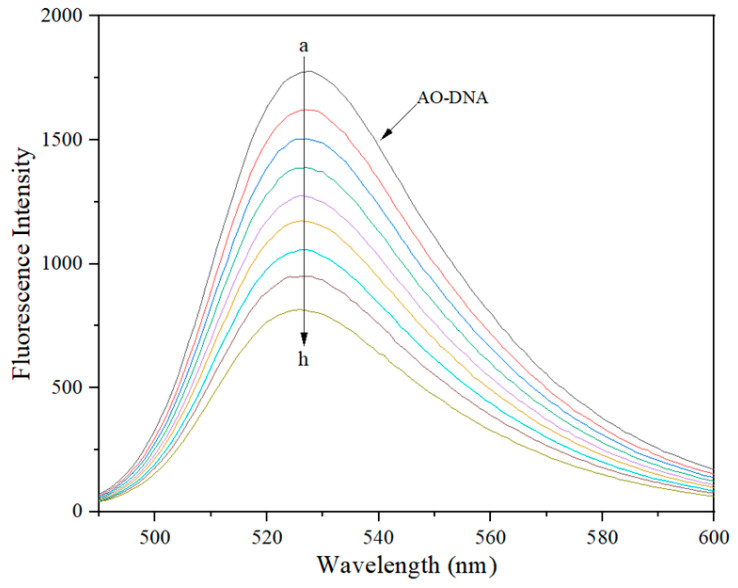
Competitive reaction between AO and quinazolone pyridinium **19a**. *c*(DNA) = 7.41 × 10^−5^ mol/L, and *c*(compound **19a**) = 0−3.111 × 10^−5^ mol/L for curves a–h respectively at increment 0.4444 × 10^− 5^ mol/L.

To identify the mode of interaction between DNA and compound **19a**, competitive studies were conducted using the classical DNA intercalator acridine orange (AO) [71,72]. With increasing concentrations of compound **19a**, a gradual decrease in the fluorescence intensity of the AO-DNA complex was observed in Figure 13, suggesting that compound **19a** can replace the DNA intercalator AO and embed into calf thymus DNA.

### 2.12. Allosteric Modulation of Compound **19a** with PBP2a

Previous studies have confirmed that the introduction of aromatic group at the *C*-2 position of quinazolinone can interact with the allosteric site of PBP2a, prompting the active site to interact with antibacterial drugs, and ultimately produce bactericidal effects [15,16]. In view of the excellent anti-MRSA activity of compound **19a**, the potential of quinazolone pyridinium **19a** as an allosteric to PBP2a was explored.

#### 2.12.1. Contents of PBP2a

The effect of compound **19a** on PBP2a was studied by measuring the changes of PBP2a content in MRSA treated with compound **19a** alone and in combination with *β*-lactam drug cefdinir using a PBP2a Elisa Assay Kit. The results in Table 2 showed that the content of PBP2a decreased after the treatment of compound **19a**. Moreover, the expressions of PBP2a treated with cefdinir and compound **19a** alone were higher than that of a combination of compound **19a** and cefdinir, indicating that quinazolone pyridinium **19a** could exert synergistic effects with cefdinir to inhibit the expression of PBP2a.

#### 2.12.2. Allosteric Site Binding Affinity of PBP2a

**Figure 14:**
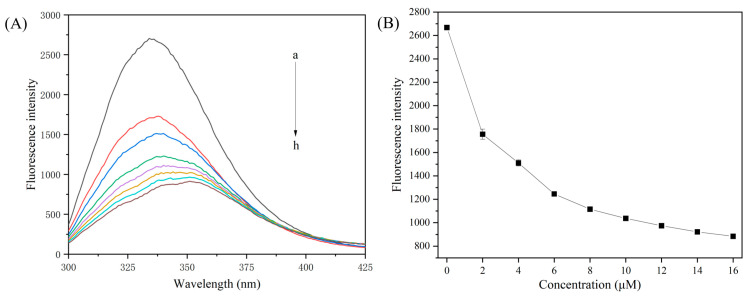
Binding of quinazolone pyridinium **19a** to the PBP2a allosteric site (λ_ex_ = 280 nm). (**A**) Emission spectra of acylated PBP2a intrinsic fluorescence with **19a** at different concentrations. *c*(compound **19a**) = 0−16 μM for curves a–h respectively at increment 2 μM. (**B**) The change in the maximum fluorescence intensity.

Binding of quinazolone pyridinium **19a** to the allosteric site was determined by monitoring the intrinsic fluorescence of tryptophan and tyrosine residues of PBP2a [73,74]. The emission spectra of individual proteins and at different concentrations of compound **19a** were measured by fluorescence photometers, and the normalized difference in fluorescence was plotted versus concentration. As illustrated in Figure 14, there was a gradual decrease in fluorescence intensity with increasing content of quinazolone pyridinium **19a**. This trend suggested a reduction in the intrinsic fluorescence of PBP2a, indicating the progressive binding of compound **19a** to PBP2a allosteric domain. In addition, a red shift could be observed distinctly.

#### 2.12.3. Molecular Docking

**Figure 15:**
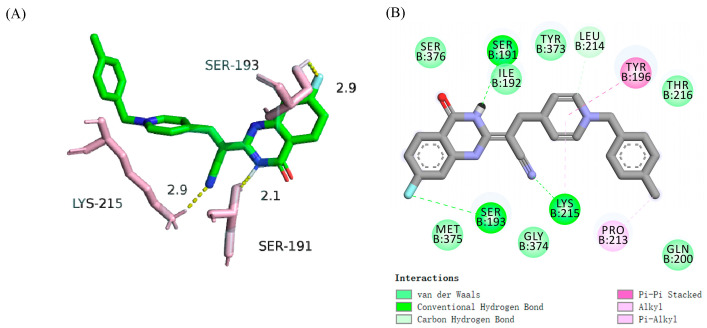
Interaction between the highly active quinazolone pyridinium **19a** with PBP2a (PDB code 4CJN) at the allosteric site. (**A**) Three-dimensional conformation of quinazolone pyridinium **19a** docked in PBP2a. Color-codes are as follows: green, compound **19a**; pink, residues that form hydrogen bonds with compound **19a**; yellow dashed lines, hydrogen bonds. (**B**) Two-dimensional conformation of quinazolone pyridinium **19a** docked in PBP2a.

Molecular docking is an important tool to helpfully understand the possible binding mode of molecules with protein [75,76], and the supramolecular interaction between active molecule **19a** and PBP2a (PDB code: 4CJN) was studied by computational simulation. As depicted in Figure 15, the amino group at *N*-3 position and the fluorine atom at *C*-7 position of quinazolinone could form hydrogen bonds with residues SER-191 and SRR-193 with distances of 2.1 and 2.9 Å, respectively. Moreover, the nitrogen atom of the cyano group was able to bind with residue LYS-215 via a hydrogen bond. Meanwhile, the aromantic conjugated skeleton of compound **19a** also provided π-π stacking interaction with PBP2a. These results indicated that quinazolone pyridinium **19a** could exert strong supramolecular binding with PBP2a by multiple hydrogen bonds, which could helpfully stabilize compound **19a**-PBP2a complex, inducing a conformational change of PBP2a.

The above results indicated that quinazolone pyridinium **19a** significantly enhanced the anti-MRSA activity of cefdinir by inducing PBP2a allosteric regulation of MRSA and triggering the opening of the active site, enabling cefdinir to bind to the active site to inhibit the expression of PBP2a.

## 3. Materials and Methods

### 3.1. Instruments and Chemicals

All the reagents and solvents were purchased commercially and could be used without purification. The melting points were recorded on the X-6 melting point instrument. The NMR spectra including ^1^H NMR and ^13^C NMR of target compounds were recorded on the Bruker AVANCE III 600 MHz or Bruker AV 400 spectrometer. The high-resolution mass spectra (HRMS) of molecules were measured by Bruker Impact II through using an ESI resource.

### 3.2. Synthesis of Intermediates and Target Molecules

#### 3.2.1. Synthesis of Intermediate **2**

A mixture of anthranilamide (300 mg, 2.20 mmol), cyanoacetic acid (225 mg, 2.64 mmol), 1-(3-dimethylaminopropyl)-3-ethylcarbodiimide hydrochloride (507 mg, 2.64 mmol), and 1-hydroxybenzotriazole hydrate (404.91 mg, 2.64 mmol) were stirred in DMF (5 mL) at room temperature for 6 h. The solution was poured into a separatory funnel and extracted three times with H_2_O/DCM system. The organic phases were combined and dried with anhydrous Na_2_SO_4_, which was further purified by silica gel column chromatography (300–400 mesh) (Eluent: petroleum ether/ethyl acetate = 3/1~1/1, *V*/*V*) to afford compound **2** as white solid (255 mg). Yield: 50.3%.

#### 3.2.2. Synthesis of Intermediate **3**

A mixture of 2-(2-cyanoacetamide) benzamide (255 mg, 1.26 mmol) and 0.5 mol/L aqueous NaOH (5 mL) were stirred at room temperature for 30 min. After the reaction was completed, 1 mol/L HCl was added to the solution to bring the pH to slightly acidic, and the mixture was filtered under reduced pressure and washed with water. The precipitate was collected and dried to afford compound **3** as white solid (225 mg). Yield: 88.2%.

#### 3.2.3. Synthesis of *(E)*-3-(Furan-2-yl)-2-(4-oxo-3,4-dihydroquinazolin-2-yl)acrylonitrile (**4a**)

A mixture of compound **3** (50 mg, 0.27 mmol), furan-2-formaldehyde (26 mg, 0.27 mmol) and piperidine (0.2 mL) was stirred in ethanol (5 mL) at 80 °C for 4 h. After the reaction was completed, solvent was removed. The crude product was purified by silica gel column chromatography (300–400 mesh) (eluent, dichloromethane/methanol = 30/1~10/1, *V*/*V*) to afford compound **4a** as yellow solid (62 mg). Yield: 87.3%, M.p.: 173–175 °C. ^1^H NMR (400 MHz, DMSO-*d*_6_) δ 8.39 (s, 1H, C=C*H*), 8.02 (d, *J* = 1.5 Hz, 1H, quinazolone-5-*H*), 7.98 (d, *J* = 7.9 Hz, 1H, furan-5-*H*), 7.50 (t, *J* = 6.7 Hz, 1H, quinazolone-7-*H*), 7.45 (d, *J* = 8.9 Hz, 1H, quinazolone-8-*H*), 7.29 (d, *J* = 3.5 Hz, 1H, furan-3-*H*), 7.19 (t, *J* = 6.6 Hz, 1H, quinazolone-6-*H*), 6.79–6.78 (m, 1H, furan-4-*H*) ppm. ^13^C NMR (101 MHz, DMSO-*d*_6_) δ 171.7, 159.2, 152.0, 150.4, 146.8, 131.8, 131.3, 126.3, 126.3, 123.5, 122.8, 118.5, 117.3, 113.7, 110.6 ppm. HRMS (ESI) calcd. for C_15_H_9_N_3_O_2_ [M + H]^+^, 264.0768; found, 264.0769.

#### 3.2.4. Synthesis of *(E)*-3-(5-Methylfuran-2-yl)-2-(4-oxo-3,4-dihydroquinazolin-2-yl)acrylonitrile (**4b**)

Compound **4b** was prepared according to the procedure described for compound **4a**, starting from compound **3** (50 mg, 0.27 mmol) and 5-methylfuran-2-formaldehyde (30 mg, 0.27 mmol). The target compound **4b** was obtained as yellow solid (55 mg). Yield: 73.5%, M.p.: 245–247 °C. ^1^H NMR (400 MHz, DMSO-*d*_6_) δ 12.48 (s, 1H, quinazolone-N*H*), 8.27 (s, 1H, C=C*H*), 8.12 (d, *J* = 7.8 Hz, 1H, quinazolone-5-*H*), 7.84 (t, *J* = 8.3 Hz, 1H, quinazolone-7-*H*), 7.71 (d, *J* = 8.0 Hz, 1H, quinazolone-8-*H*), 7.54 (t, *J* = 7.3 Hz, 1H, quinazolone-6-*H*), 7.33 (d, *J* = 3.4 Hz, 1H, furan-3-*H*), 6.56 (d, *J* = 3.3 Hz, 1H, furan-4-*H*), 2.45 (s, 3H, furan-C*H*_3_) ppm. ^13^C NMR (101 MHz, DMSO-*d*_6_) δ 162.1, 159.5, 149.7, 148.6, 147.8, 135.3, 134.2, 127.9, 127.6, 126.4, 123.5, 121.4, 116.4, 111.7, 99.7, 14.4 ppm. HRMS (ESI) calcd. for C_16_H_11_N_3_O_2_ [M + H]^+^, 278.0924; found, 278.0923.

#### 3.2.5. Synthesis of *(E)*-3-(5-(Hydroxymethyl)furan-2-yl)-2-(4-oxo-3,4-dihydroquinazolin-2-yl)acrylonitrile (**4c**)

Compound **4c** was prepared according to the procedure described for compound **4a**, starting from compound **3** (50 mg, 0.27 mmol) and 5-(hydroxymethyl)-furan-2-formaldehyde (34 mg, 0.27 mmol). The target compound **4c** was obtained as yellow solid (53 mg). Yield: 66.9%, M.p.: > 300 °C. ^1^H NMR (400 MHz, DMSO-*d*_6_) δ 12.51 (s, 1H, quinazolone-N*H*), 8.33 (s, 1H, C=C*H*), 8.13 (d, *J* = 6.7 Hz, 1H, quinazolone-5-*H*), 7.85 (t, *J* = 6.9 Hz, 1H, quinazolone-7-*H*), 7.71 (d, *J* = 7.8 Hz, 1H, quinazolone-8-*H*), 7.55 (t, *J* = 7.0 Hz, 1H, quinazolone-6-*H*), 7.40 (d, *J* = 3.6 Hz, 1H, furan-3-*H*), 6.71 (d, *J* = 3.5 Hz, 1H, furan-4-*H*), 5.57 (t, *J* = 5.8 Hz, 1H, CH_2_-O*H*), 4.56 (d, *J* = 5.6 Hz, 2H, C*H*_2_-OH) ppm. ^13^C NMR (101 MHz, DMSO-*d*_6_) δ 162.2, 162.1, 149.6, 148.6, 148.3, 135.3, 134.7, 127.9, 127.7, 126.4, 122.2, 121.5, 116.2, 111.5, 101.0, 56.5 ppm. HRMS (ESI) calcd. for C_16_H_11_N_3_O_3_ [M + H]^+^, 294.0873; found, 294.0873.

#### 3.2.6. Synthesis of *(E)*-2-(4-Oxo-3,4-dihydroquinazolin-2-yl)-3-(thiophen-2-yl)acrylonitrile (**5a**)

Compound **5a** was prepared according to the procedure described for compound **4a**, starting from compound **3** (50 mg, 0.27 mmol) and thiophene-2-formaldehyde (30 mg, 0.27 mmol). The target compound **5a** was obtained as yellow solid (70 mg). Yield: 92.8%, M.p.: 176–178 °C. ^1^H NMR (400 MHz, DMSO-*d*_6_) δ 12.52 (s, 1H, quinazolone-N*H*), 8.75 (s, 1H, C=C*H*), 8.19–8.12 (m, 2H, quinazolone-5-*H*, thiophene-5-*H*), 7.88–7.83 (m, 2H, quinazolone-8-*H*, quinazolone-7-*H*), 7.72 (d, *J* = 7.7 Hz, 1H, thiophene-3-*H*), 7.55 (t, *J* = 7.0 Hz, 1H, quinazolone-6-*H*), 7.38–7.36 (m, 1H, thiophene-4-*H*) ppm. ^13^C NMR (101 MHz, DMSO-*d*_6_) δ 162.1, 149.4, 148.6, 142.2, 137.7, 136.6, 135.7, 135.3, 129.2, 127.9, 127.7, 126.4, 121.5, 116.6, 102.5 ppm. HRMS (ESI) calcd. for C_15_H_9_N_3_OS [M + H]^+^, 280.0539; found, 280.0538.

#### 3.2.7. Synthesis of *(E)*-3-(3-Methylthiophen-2-yl)-2-(4-oxo-3,4-dihydroquinazolin-2-yl)acrylonitrile (**5b**)

Compound **5b** was prepared according to the procedure described for compound **4a**, starting from compound **3** (50 mg, 0.27 mmol) and 3-methylthiophene-2-formaldehyde (34 mg, 0.27 mmol). The target compound **5b** was obtained as yellow solid (53 mg). Yield: 66.9%, M.p.: 285–287 °C. ^1^H NMR (400 MHz, DMSO-*d*_6_) δ 12.73 (s, 1H, quinazolone-N*H*), 8.84 (d, *J* = 5.6 Hz, 1H, quinazolone-5-*H*,), 8.51 (s, 1H, C=C*H*), 8.18 (d, *J* = 7.9 Hz, 1H, thiophene-5-*H*), 7.89 (t, *J* = 7.7 Hz, 1H, quinazolone-7-*H*), 7.82 (d, *J* = 7.7 Hz, 1H, quinazolone-8-*H*), 7.70 (d, *J* = 8.1 Hz, 1H, thiophene-4-*H*), 7.61 (t, *J* = 7.5 Hz, 1H, quinazolone-6-*H*), 2.51 (s, 3H, thiophene-C*H*_3_) ppm. ^13^C NMR (101 MHz, DMSO-*d*_6_) δ 162.0, 151.3, 149.4, 147.4, 139.8, 135.5, 134.3, 131.8, 128.4, 126.5, 123.4, 121.9, 115.4, 111.5, 101.4, 15.1 ppm. HRMS (ESI) calcd. for C_16_H_11_N_3_OS [M + H]^+^, 294.0696; found, 294.0694

#### 3.2.8. Synthesis of *(E)*-3-(1H-Imidazol-2-yl)-2-(4-oxo-3,4-dihydroquinazolin-2-yl)acrylonitrile (**6a**)

Compound **6a** was prepared according to the procedure described for compound **4a**, starting from compound **3** (50 mg, 0.27 mmol) and imidazole-2-formaldehyde (26 mg, 0.27 mmol). The target compound **6a** was obtained as yellow solid (35 mg). Yield: 49.2%, M.p.: 243–245 °C. ^1^H NMR (400 MHz, DMSO-*d*_6_) δ 8.31 (s, 1H, C=C*H*), 8.17 (d, *J* = 7.9 Hz, 1H, quinazolone-5-*H*), 7.89 (t, *J* = 6.9 Hz, 1H, quinazolone-7-*H*), 7.76 (d, *J* = 8.1 Hz, 1H, quinazolone-8-*H*), 7.71 (m, 2H, imidazole-4-*H*, imidazole-5-*H*), 7.60 (t, *J* = 7.5 Hz, 1H, quinazolone-6-*H*) ppm. ^13^C NMR (101 MHz, DMSO-*d*_6_) δ 162.2, 149.2, 148.3, 141.1, 139.3, 135.4, 133.8, 133.0, 128.2, 128.0, 126.5, 121.7, 115.3, 108.9 ppm. HRMS (ESI) calcd. for C_14_H_9_N_5_O [M + H]^+^, 264.0880; found, 264.0880.

#### 3.2.9. Synthesis of *(E)*-2-(4-Oxo-3,4-dihydroquinazolin-2-yl)-3-(thiazol-2-yl)acrylonitrile (**6b**)

Compound **6b** was prepared according to the procedure described for compound **4a**, starting from compound **3** (50 mg, 0.27 mmol) and thiazole-2-formaldehyde (34 mg, 0.27 mmol). The target compound **6b** was obtained as yellow solid (59 mg). Yield: 78.0%, M.p.: 245–247 °C. ^1^H NMR (400 MHz, DMSO-*d*_6_) δ 12.76 (s, 1H, quinazolone-N*H*), 8.75 (s, 1H, C=C*H*), 8.30 (m, 2H, quinazolone-5-*H*, thiazole-4-*H*), 8.17 (d, *J* = 6.9 Hz, 1H, thiazole-5-*H*), 7.88 (t, *J* = 6.9 Hz, 1H, quinazolone-7-*H*), 7.77 (d, *J* = 7.9 Hz, 1H, quinazolone-8-*H*), 7.60 (t, *J* = 7.1 Hz, 1H, quinazolone-6-*H*) ppm. ^13^C NMR (101 MHz, DMSO-*d*_6_) δ 162.0, 159.7, 148.9, 148.3, 146.1, 140.4, 135.4, 128.3, 128.2, 127.4, 126.5, 121.8, 115.7, 108.5 ppm. HRMS (ESI) calcd. for C_14_H_8_N_4_OS [M + H]^+^, 281.0492; found, 281.0492.

#### 3.2.10. Synthesis of *(E)*-2-(4-Oxo-3,4-dihydroquinazolin-2-yl)-3-(1H-pyrrol-2-yl)acrylonitrile (**6c**)

Compound **6c** was prepared according to the procedure described for compound **4a**, starting from compound **3** (50 mg, 0.27 mmol) and pyrrole-2-aldehyde (26 mg, 0.27 mmol). The target compound **6c** was obtained as yellow solid (49 mg). Yield: 69.2%, M.p.: >300 °C. ^1^H NMR (400 MHz, DMSO-*d*_6_) δ 11.70 (s, 1H, pyrrole-N*H*), 8.51 (s, 1H, C=C*H*), 7.96 (d, *J* = 6.7 Hz, 1H, quinazolone-5-*H*), 7.51–7.43 (m, 1H, quinazolone-7-*H*), 7.39 (d, *J* = 7.5 Hz, 1H, quinazolone-8-*H*), 7.24 (d, *J* = 7.9 Hz, 1H, pyrrole-5-*H*), 7.15 (d, *J* = 8.0 Hz, 1H, pyrrole-3-*H*), 6.36–6.24 (m, 1H, pyrrole-4-*H*) ppm. ^13^C NMR (101 MHz, DMSO-*d*_6_) δ 171.6, 160.1, 152.2, 135.8, 131.2, 128.1, 126.3, 126.0, 123.9, 122.9, 122.5, 120.2, 113.3, 111.8, 105.4 ppm. HRMS (ESI) calcd. for C_15_H_10_N_4_O [M + H]^+^, 263.0927; found, 263.0929.

#### 3.2.11. Synthesis of *(E)*-3-(2-Methylthiazol-5-yl)-2-(4-oxo-3,4-dihydroquinazolin-2-yl)acrylonitrile (**7**)

Compound **7** was prepared according to the procedure described for compound **4a**, starting from compound **3** (50 mg, 0.27 mmol) and 2-methylthiazole-5-formaldehyde (35 mg, 0.27 mmol). The target compound **7** was obtained as yellow solid (57 mg). Yield: 71.7%, M.p.: 245–247 °C. ^1^H NMR (400 MHz, DMSO-*d*_6_) δ 12.57 (s, 1H, quinazolone-N*H*), 8.76 (s, 1H, C=C*H*), 8.25 (s, 1H, thiazole-4-*H*), 8.14 (d, *J* = 6.7 Hz, 1H, quinazolone-5-*H*), 7.86 (t, *J* = 6.9 Hz, 1H, quinazolone-7-*H*), 7.72 (d, *J* = 7.7 Hz, 1H, quinazolone-8-*H*), 7.56 (t, *J* = 7.0 Hz, 1H, quinazolone-6-*H*) ppm. ^13^C NMR (101 MHz, DMSO-*d*_6_) δ 173.7, 159.6, 152.5, 149.1, 140.6, 140.4, 135.4, 132.0, 128.0, 127.9, 126.5, 121.6, 116.4, 101.1, 20.0 ppm. HRMS (ESI) calcd. for C_15_H_10_N_4_OS [M + H]^+^, 295.0648; found, 295.0648.

#### 3.2.12. Synthesis of *(E)*-3-(1H-Imidazol-4-yl)-2-(4-oxo-3,4-dihydroquinazolin-2-yl)acrylonitrile (**8a**)

Compound **8a** was prepared according to the procedure described for compound **4a**, starting from compound **3** (50 mg, 0.27 mmol) and imidazole-4-formaldehyde (26 mg, 0.27 mmol). The target compound **8a** was obtained as yellow solid (51 mg). Yield: 71.8%, M.p.: >300 °C. ^1^H NMR (400 MHz, DMSO-*d*_6_) δ 12.66 (s, 2H, quinazolone-N*H*, imidazole-N*H*), 8.40 (s, 1H, C=C*H*), 8.13 (d, *J* = 7.9 Hz, 1H, quinazolone-5-*H*), 8.04 (s, 1H, imidazole-2-*H*), 8.00 (s, 1H, imidazole-5-*H*), 7.84 (t, *J* = 6.9 Hz, 1H, quinazolone-7-*H*), 7.70 (d, *J* = 7.8 Hz, 1H, quinazolone-8-*H*), 7.53 (t, *J* = 7.0 Hz, 1H, quinazolone-6-*H*) ppm. ^13^C NMR (101 MHz, DMSO-*d*_6_) δ 162.2, 150.1, 148.7, 139.3, 138.9, 135.2, 135.0, 129.1, 127.7, 127.4, 126.4, 121.4, 116.9, 101.2 ppm. HRMS (ESI) calcd. for C_14_H_9_N_5_O [M + H]^+^, 264.0880; found, 264.0879.

#### 3.2.13. Synthesis of *(E)*-3-(2-Butyl-5-chloro-1H-imidazol-4-yl)-2-(4-oxo-3,4-dihydroquinazolin-2-yl)acrylonitrile (**8b**)

Compound **8b** was prepared according to the procedure described for compound **4a**, starting from compound **3** (50 mg, 0.27 mmol) and 2-butyl-5-chloro-1*H*-imidazole-4-aldehyde (50 mg, 0.27 mmol). The target compound **8b** was obtained as yellow solid (89 mg). Yield: 93.2%, M.p.: 173–175 °C. ^1^H NMR (400 MHz, DMSO-*d*_6_) δ 8.07 (d, *J* = 7.8 Hz, 1H, quinazolone-5-*H*), 7.74 (t, *J* = 7.6 Hz, 1H, quinazolone-7-*H*), 7.58 (d, *J* = 8.2 Hz, 1H, quinazolone-8-*H*), 7.41 (t, *J* = 7.4 Hz, 1H, quinazolone-6-*H*), 7.21 (s, 1H, C=C*H*), 2.70–2.59 (m, 2H, C*H*_2_CH_2_CH_2_CH_3_), 1.80 (q, *J* = 7.5 Hz, 2H, C*H*_2_CH_2_CH_2_CH_3_), 1.35 (q, *J* = 7.4 Hz, 2H, CH_2_CH_2_C*H*_2_CH_3_), 0.93 (t, *J* = 7.3 Hz, 3H, CH_2_CH_2_CH_2_C*H*_3_) ppm. ^13^C NMR (101 MHz, DMSO-*d*_6_) δ 163.3, 159.6, 152.8, 149.8, 141.1, 135.1, 134.3, 129.0, 127.1, 126.2, 125.7, 121.9, 121.0, 31.2, 30.0, 22.1, 14.3 ppm. HRMS (ESI) calcd. for C_18_H_16_ClN_5_O [M + H]^+^, 354.1116; found, 354.1115.

#### 3.2.14. Synthesis of *(E)*-2-(4-Oxo-3,4-dihydroquinazolin-2-yl)-3-(pyridin-2-yl)acrylonitrile (**9a**)

Compound **9a** was prepared according to the procedure described for compound **4a**, starting from compound **3** (50 mg, 0.27 mmol) and pyridine-2-formaldehyde (29 mg, 0.27 mmol). The target compound **9a** was obtained as yellow solid (24 mg). Yield: 32.4%, M.p.: 237–239 °C. ^1^H NMR (400 MHz, DMSO-*d*_6_) δ 12.69 (s, 1H, quinazolone-N*H*), 8.83 (d, *J* = 5.5 Hz, 1H, pyridine-6-*H*), 8.46 (s, 1H, C=C*H*), 8.18 (d, *J* = 7.9 Hz, 1H, quinazolone-5-*H*), 8.04 (t, *J* = 7.9 Hz, 1H, quinazolone-7-*H*), 7.89 (t, *J* = 6.9 Hz, 1H, pyridine-5-*H*), 7.82 (d, *J* = 7.8 Hz, 1H, quinazolone-8-*H*), 7.78 (d, *J* = 7.7 Hz, 1H, pyridine-3-*H*), 7.62–7.60 (m, 1H, quinazolone-6-*H*), 7.58–7.56 (m, 1H, pyridine-4-*H*) ppm. ^13^C NMR (101 MHz, DMSO-*d*_6_) δ 162.1, 150.8, 150.6, 149.5, 148.4, 148.1, 138.0, 135.4, 128.2, 127.7, 126.6, 126.5, 121.9, 115.4, 109.5 ppm. HRMS (ESI) calcd. for C_16_H_10_N_4_O [M + H]^+^, 275.0927; found, 275.0930.

#### 3.2.15. Synthesis of *(E)*-2-(4-Oxo-3,4-dihydroquinazolin-2-yl)-3-(pyridin-3-yl)acrylonitrile (**9b**)

Compound **9b** was prepared according to the procedure described for compound **4a**, starting from compound **3** (50 mg, 0.27 mmol) and pyridine-3-formaldehyde (29 mg, 0.27 mmol). The target compound **9b** was obtained as yellow solid (55 mg). Yield: 74.3%, M.p.: 263–265 °C. ^1^H NMR (400 MHz, DMSO-*d*_6_) δ 12.72 (s, 1H, quinazolone-N*H*), 8.99 (s, 1H. pyridine-2-*H*), 8.76 (d, *J* = 3.5 Hz, 1H, pyridine-6-*H*), 8.55 (s, 1H, C=C*H*), 8.46 (d, *J* = 8.1 Hz, 1H, pyridine-4-*H*), 8.17 (d, *J* = 7.8 Hz, 1H, quinazolone-5-*H*), 7.88 (t, *J* = 7.6 Hz, 1H, quinazolone-7-*H*), 7.76 (d, *J* = 8.0 Hz, 1H, quinazolone-8-*H*), 7.67 (m, 1H, pyridine-5-*H*), 7.59 (t, *J* = 7.5 Hz, 1H, quinazolone-6-*H*) ppm. ^13^C NMR (101 MHz, DMSO-*d*_6_) δ 162.1, 152.8, 151.6, 149.1, 148.3, 146.7, 136.3, 135.5, 128.9, 128.2, 128.1, 126.5, 124.7, 121.8, 116.0, 109.1 ppm. HRMS (ESI) calcd. for C_16_H_10_N_4_O [M + H]^+^, 275.0927; found, 275.0928.

#### 3.2.16. Synthesis of *(E)*-2-(4-Oxo-3,4-dihydroquinazolin-2-yl)-3-(pyridin-4-yl)acrylonitrile (**9c**)

Compound **9c** was prepared according to the procedure described for compound **4a**, starting from compound **3** (50 mg, 0.27 mmol) and pyridine-4-formaldehyde (29 mg, 0.27 mmol). The target compound **9c** was obtained as yellow solid (41 mg). Yield: 55.4%, M.p.: 272–274 °C. ^1^H NMR (400 MHz, DMSO-*d*_6_) δ 12.73 (s, 1H, quinazolone-N*H*), 8.88–8.82 (m, 2H, pyridine-2-*H*, pyridine-6-*H*), 8.51 (s, 1H, C=C*H*), 8.18 (d, *J* = 6.7 Hz, 1H, quinazolone-5-*H*), 7.89 (t, *J* = 6.9 Hz, 1H, quinazolone-7-*H*), 7.84–7.81 (m, 2H, pyridine-3-*H*, pyridine-5-*H*), 7.78 (d, *J* = 7.7 Hz, 1H, quinazolone-8-*H*), 7.61 (t, *J* = 7.0 Hz, 1H, quinazolone-6-*H*) ppm. ^13^C NMR (101 MHz, DMSO-*d*_6_) δ 162.0, 151.3, 148.8, 148.2, 147.4, 139.8, 135.5, 128.4, 128.2, 126.5, 123.4, 121.9, 115.4, 111.5 ppm. HRMS (ESI) calcd. for C_16_H_10_N_4_O [M + H]^+^, 275.0927; found, 275.0927.

#### 3.2.17. Synthesis of *(E)*-3-(2-Methoxypyrimidin-5-yl)-2-(4-oxo-3,4-dihydroquinazolin-2-yl)acrylonitrile (**10**)

Compound **10** was prepared according to the procedure described for compound **4a**, starting from compound **3** (50 mg, 0.27 mmol) and 2-methoxypyrimidin-5-formaldehyde (37 mg, 0.27 mmol). The target compound **10** was obtained as yellow solid (78 mg). Yield: 94.7%, M.p.: 235–237 °C. ^1^H NMR (600 MHz, DMSO-*d*_6_) δ 9.18 (s, 2H, pyrimidine-4-*H*, pyrimidine-6-*H*), 8.52 (s, 1H, C=C*H*), 8.02–8.01 (m, 1H, quinazolone-5-*H*), 7.61–7.55 (m, 1H, quinazolone-7-*H*), 7.53 (d, *J* = 8.1 Hz, 1H, quinazolone-8-*H*), 7.31–7.23 (m, 1H, quinazolone-6-*H*), 4.02 (s, 3H, pyrimidine-C*H*_3_) ppm. ^13^C NMR (151 MHz, DMSO-*d*_6_) δ 170.6, 165.7, 160.6, 157.5, 151.3, 140.0, 132.1, 126.7, 126.3, 124.5, 122.4, 122.4, 118.5, 114.5, 55.7 ppm. HRMS (ESI) calcd. for C_16_H_11_N_5_O_2_ [M + Na]^+^, 328.0805; found, 328.0791.

#### 3.2.18. Synthesis of *(E)*-3-(1H-Benzo[d]imidazol-2-yl)-2-(4-oxo-3,4-dihydroquinazolin-2-yl)acrylonitrile (**11a**)

Compound **11a** was prepared according to the procedure described for compound **4a**, starting from compound **3** (50 mg, 0.27 mmol) and benzimidazole-2-formaldehyde (34 mg, 0.27 mmol). The target compound **11a** was obtained as yellow solid (62 mg). Yield: 73.3%, M.p.: > 300 °C. ^1^H NMR (400 MHz, DMSO-*d*_6_) δ 8.61 (s, 1H, C=C*H*), 8.03 (d, *J* = 7.7 Hz, 1H, quinazolone-5-*H*), 7.74–7.71 (m, 1H, quinazolone-7-*H*), 7.70–7.66 (m, 2H, quinazolone-6-*H*, quinazolone-8-*H*), 7.56–7.49 (m, 2H, benzimidazole-4-*H*, benzimidazole-7-*H*), 7.29–7.27 (m, 2H, benzimidazole-5-*H*, benzimidazole-6-*H*) ppm. ^13^C NMR (101 MHz, DMSO-*d*_6_) δ 171.6, 167.3, 159.1, 155.4, 151.9, 150.3, 147.7, 139.2, 133.2, 131.5, 127.2, 126.4, 124.0, 123.6, 123.0, 120.5, 117.5, 114.1 ppm. HRMS (ESI) calcd. for C_18_H_11_N_5_O [M + H]^+^, 314.1036; found, 314.1039.

#### 3.2.19. Synthesis of *(E)*-3-(Benzo[d]thiazol-2-yl)-2-(4-oxo-3,4-dihydroquinazolin-2-yl)acrylonitrile (**11b**)

Compound **11b** was prepared according to the procedure described for compound **4a**, starting from compound **3** (50 mg, 0.27 mmol) and benzothiazol-2-aldehyde (40 mg, 0.27 mmol). The target compound **11b** was obtained as yellow solid (64 mg). Yield: 71.5%, M.p.: >300 °C. ^1^H NMR (400 MHz, DMSO-*d*_6_) δ 12.83 (s, 1H, quinazolone-N*H*), 8.83 (s, 1H, C=C*H*), 8.33 (d, *J* = 7.6 Hz, 1H, benzothiazole-4-*H*), 8.23 (d, *J* = 8.4 Hz, 1H, benzothiazole-7-*H*), 8.19 (d, *J* = 6.6 Hz, 1H, quinazolone-5-*H*), 7.90 (t, *J* = 6.9 Hz, 1H, quinazolone-7-*H*), 7.81 (d, *J* = 7.9 Hz, 1H, quinazolone-8-*H*), 7.71–7.66 (m, 1H, benzothiazole-5-*H*), 7.66 -7.64 (m, 1H, benzothiazole-6-*H*), 7.62–7.60 (m, 1H, quinazolone-6-*H*) ppm. ^13^C NMR (101 MHz, DMSO-*d*_6_) δ 171.8, 165.9, 160.1, 159.2, 153.1, 151.8, 148.2, 141.0, 136.4, 135.5, 128.6, 128.4, 128.2, 126.5, 124.7, 123.4, 118.6, 115.4 ppm. HRMS (ESI) calcd. for C_18_H_11_N_5_O [M + H]^+^, 331.0648; found, 331.0650.

#### 3.2.20. Synthesis of *(E)*-3-(1H-Indol-3-yl)-2-(4-oxo-3,4-dihydroquinazolin-2-yl)acrylonitrile (**12a**)

Compound **12a** was prepared according to the procedure described for compound **4a**, starting from compound **3** (50 mg, 0.27 mmol) and indole-3-carboxaldehyde (47 mg, 0.27 mmol). The target compound **12a** was obtained as yellow solid (68 mg). Yield: 80.6%, M.p.: >300 °C. ^1^H NMR (400 MHz, DMSO-*d*_6_) δ 12.50 (s, indole-N*H*), 8.88 (s, 1H, indole-2-*H*), 8.60 (s, 1H, C=C*H*), 8.18–8.07 (m, 2H, quinazolone-5-*H*, indole-4-*H*), 7.83 (t, *J* = 7.0 Hz, 1H, quinazolone-7-*H*), 7.69 (d, *J* = 8.0 Hz, 1H, indole-7-*H*), 7.59–7.55 (m, 1H, quinazolone-8-*H*), 7.51 (t, *J* = 7.5 Hz, 1H, indole-6-*H*), 7.32–7.24 (m, 2H, indole-5-*H*, quinazolone-6-*H*) ppm. ^13^C NMR (101 MHz, DMSO-*d*_6_) δ 162.6, 150.6, 149.0, 140.8, 136.3, 135.2, 130.6, 128.2, 127.6, 127.0, 126.4, 123.8, 122.0, 121.2, 119.3, 118.8, 113.2, 110.7, 98.2 ppm. HRMS (ESI) calcd. for C_19_H_12_N_4_O [M + H]^+^, 313.1084; found, 313.1085.

#### 3.2.21. Synthesis of *(E)*-3-(Benzo[b]thiophen-3-yl)-2-(4-oxo-3,4-dihydroquinazolin-2-yl)acrylonitrile (**12b**)

Compound **12b** was prepared according to the procedure described for compound **4a**, starting from compound **3** (50 mg, 0.27 mmol) and benzothiophene-3-formaldehyde (53 mg, 0.27 mmol). The target compound **12b** was obtained as yellow solid (83 mg). Yield: 93.3%, M.p.: 273–275 °C. ^1^H NMR (400 MHz, DMSO-*d*_6_) δ 12.90 (s, 1H, quinazolone-N*H*), 8.98 (s, 1H, benzothiophene-2-*H*), 8.77 (s, 1H, C=C*H*), 8.45 (d, *J* = 8.0 Hz, 1H, benzothiophene-4-*H*), 8.21–8.11 (m, 2H, benzothiophene-7-*H*, quinazolone-5-*H*), 7.87 (t, *J* = 7.6 Hz, 1H, quinazolone-7-*H*), 7.76 (d, *J* = 8.1 Hz, 1H, quinazolone-8-*H*), 7.63–7.57 (m, 2H, benzothiophene-5-*H*, benzothiophene-6-*H*), 7.57–7.51 (m, 1H, quinazolone-6-*H*) ppm. ^13^C NMR (101 MHz, DMSO-*d*_6_) δ 162.3, 150.3, 149.5, 139.3, 139.0, 138.6, 135.4, 133.5, 128.7, 128.1, 127.9, 126.5, 126.2, 125.6, 123.6, 122.7, 117.1, 113.3, 107.1 ppm. HRMS (ESI) calcd. for C_19_H_11_N_3_OS [M + H]^+^, 330.0696; found, 330.0711.

#### 3.2.22. Synthesis of *(E)*-3-(Benzofuran-3-yl)-2-(4-oxo-3,4-dihydroquinazolin-2-yl)acrylonitrile (**12c**)

Compound **12c** was prepared according to the procedure described for compound **4a**, starting from compound **3** (50 mg, 0.27 mmol) and benzofuran--3-formaldehyde (47 mg, 0.27 mmol). The target compound **12c** was obtained as yellow solid (73 mg). Yield: 86.3%, M.p.: 272–274 °C. ^1^H NMR (400 MHz, DMSO-*d*_6_) δ 10.24 (s, 1H, quinazolone-N*H*), 9.24 (s, 1H, benzofuran-2-*H*), 8.77 (s, 1H, C=C*H*), 8.34 (d, *J* = 6.9 Hz, 1H, quinazolone-5-*H*), 7.99 (t, *J* = 8.4 Hz, 1H, quinazolone-7-*H*), 7.84 (d, *J* = 8.2 Hz, 1H, benzofuran-4-*H*), 7.61 (t, *J* = 8.0 Hz, 1H, quinazolone-6-*H*), 7.55 (d, *J* = 6.2 Hz, 1H, quinazolone-8-*H*), 7.29 (t, *J* = 6.9 Hz, 1H, benzofuran-6-*H*), 7.03 (d, *J* = 7.3 Hz, 1H, benzofuran-7-*H*), 6.97 (t, *J* = 8.0 Hz, 1H, benzofuran-5-*H*) ppm. ^13^C NMR (101 MHz, DMSO-*d*_6_) δ 158.4, 155.2, 147.7, 146.4, 144.7, 136.2, 130.7, 130.0, 129.8, 127.5, 127.4, 126.6, 122.4, 121.1, 120.4, 116.8, 116.8, 115.9, 109.4 ppm. HRMS (ESI) calcd. for C_19_H_11_N_3_O_2_ [M + H]^+^, 314.0924; found, 314.0926.

#### 3.2.23. Synthesis of *(E)*-1-Benzyl-4-(2-cyano-2-(4-oxo-3,4-dihydroquinazolin-2-yl)vinyl)pyridin-1-ium (**13a**)

A mixture of compound **9c** (50 mg, 0.18 mmol) and benzyl bromide (62 mg, 0.36 mmol) was stirred in acetonitrile (5 mL) at 80 °C for 12 h. After the reaction was completed, the solvent was removed to give a crude product, which was purified by silica gel column chromatography (300–400 mesh) (eluent, dichloromethane/methanol = 30/1~10/1, *V*/*V*) to afford compound **13a** as yellow solid (74 mg). Yield: 91.2%, M.p.: 239–241 °C. ^1^H NMR (400 MHz, DMSO-*d*_6_) δ 12.86 (s, 1H, quinazolone-N*H*), 9.39–9.37 (m, 2H, pyridine-2-*H*, pyridine-6-*H*), 8.68 (s, 1H, C=C*H*), 8.50–8.48 (m, 2H, pyridine-3-*H*, pyridine-5-*H*), 8.20 (d, *J* = 9.1 Hz, 1H, quinazolone-5-*H*), 7.92 (t, *J* = 7.7 Hz, 1H, quinazolone-7-*H*), 7.81 (d, *J* = 7.9 Hz, 1H, quinazolone-8-*H*), 7.66 (t, *J* = 7.5 Hz, 1H, quinazolone-6-*H*), 7.61 (d, *J* = 6.0 Hz, 2H, Ph-2-*H*, Ph-6-*H*), 7.49 (m, 3H, Ph-3-*H*, Ph-4-*H*, Ph-5-*H*), 5.95 (s, 2H, C*H*_2_-Ph) ppm. ^13^C NMR (101 MHz, DMSO-*d*_6_) δ 161.9, 148.4, 148.2, 148.0, 146.1, 143.7, 135.7, 134.3, 130.0, 129.8, 129.6, 129.0, 128.5, 128.1, 126.6, 122.0, 116.0, 114.6, 64.0 ppm. HRMS (ESI) calcd. for C_23_H_17_N_4_O^+^Br^−^ [M − Br^−^]^+^, 365.1397; found, 365.1397.

#### 3.2.24. Synthesis of *(E)*-4-(2-Cyano-2-(4-oxo-3,4-dihydroquinazolin-2-yl)vinyl)-1-(4-methylbenzyl)pyridin-1-ium (**13b**)

Compound **13b** was prepared according to the procedure described for compound **13a**, starting from compound **9c** (50 mg, 0.18 mmol) and *p*-methylbenzyl bromide (67 mg, 0.36 mmol). The target compound **13b** was obtained as yellow solid (80 mg). Yield: 95.5%, M.p.: 253–255 °C. ^1^H NMR (400 MHz, DMSO-*d*_6_) δ 12.86 (s, 1H, quinazolone-N*H*), 9.39–9.37 (m, 2H, pyridine-2-*H*, pyridine-6-*H*), 8.69 (s, 1H, C=C*H*), 8.50–8.48 (m, 2H, pyridine-3-*H*, pyridine-5-*H*), 8.20 (d, *J* = 6.9 Hz, 1H, quinazolone-5-*H*), 7.92 (t, *J* = 7.0 Hz, 1H, quinazolone-7-*H*), 7.81 (d, *J* = 8.0 Hz, 1H, quinazolone-8-*H*), 7.65 (t, *J* = 7.5 Hz, 1H, quinazolone-6-*H*), 7.52 (d, *J* = 8.0 Hz, 2H, Ph-2-*H*, Ph-6-*H*), 7.30 (d, *J* = 7.9 Hz, 2H, Ph-3-*H*, Ph-5-*H*,), 5.91 (s, 2H, C*H*_2_-Ph), 2.33 (s, 3H, Ph-C*H*_3_) ppm. ^13^C NMR (101 MHz, DMSO-*d*_6_) δ 161.9, 148.3, 148.2, 148.0, 146.0, 143.7, 139.7, 135.7, 131.4, 130.3, 129.7, 129.0, 128.5, 128.0, 126.6, 122.0, 115.9, 114.6, 63.8, 21.3 ppm. HRMS (ESI) calcd. for C_24_H_19_N_4_O^+^Br^−^ [M − Br^−^]^+^, 379.1553; found, 379.1552.

#### 3.2.25. Synthesis of *(E)*-1-(4-(tert-Butyl)benzyl)-4-(2-cyano-2-(4-oxo-3,4-dihydroquinazolin-2-yl)vinyl)pyridine -1-ium (**13c**)

Compound **13c** was prepared according to the procedure described for compound **13a**, starting from compound **9c** (50 mg, 0.18 mmol) and *p*-tert butyl benzyl bromide (85 mg, 0.36 mmol). The target compound **13c** was obtained as yellow solid (80 mg). Yield: 93.0%, M.p.: 238–240 °C. ^1^H NMR (400 MHz, DMSO-*d*_6_) δ 12.87 (s, 1H, quinazolone-N*H*), 9.39–9.37 (m, 2H, pyridine-2-*H*, pyridine-6-*H*) 8.69 (s, 1H, C=C*H*), 8.50–8.48 (m, 2H, pyridine-3-*H*, pyridine-5-*H*), 8.20 (d, *J* = 9.1 Hz, 1H, quinazolone-5-*H*), 7.92 (t, *J* = 6.9 Hz, 1H, quinazolone-7-*H*), 7.81 (d, *J* = 7.6 Hz, 1H, quinazolone-8-*H*), 7.65 (t, *J* = 7.0 Hz, 1H, quinazolone-6-*H*), 7.55 (d, *J* = 8.5 Hz, 2H, Ph-2-*H*, Ph-6-*H*), 7.50 (d, *J* = 8.6 Hz, 2H, Ph-3-*H*, Ph-5-*H*), 5.91 (s, 2H, C*H*_2_-Ph), 1.28 [s, 9H, C(C*H*_3_)_3_] ppm. ^13^C NMR (101 MHz, DMSO-*d*_6_) δ 161.9, 152.7, 148.3, 148.2, 148.0, 146.0, 143.7, 135.7, 131.5, 129.4, 129.0, 128.4, 128.1, 126.7, 126.6, 122.0, 115.9, 114.6, 63.7, 34.9, 31.5 ppm. HRMS (ESI) calcd. for C_27_H_25_N_4_O^+^Br^−^ [M − Br^−^]^+^, 421.2023; found, 421.2022.

#### 3.2.26. Synthesis of *(E)*-4-(2-Cyano-2-(4-oxo-3,4-dihydroquinazolin-2-yl)vinyl)-1-(4-fluorobenzyl)pyridin-1-ium (**13d**)

Compound **13d** was prepared according to the procedure described for compound **13a**, starting from compound **9c** (50 mg, 0.18 mmol) and *p*-fluorobenzyl bromide (70 mg, 0.36 mmol). The target compound **13d** was obtained as yellow solid (80 mg). Yield: 94.1%, M.p.: 231–243 °C. ^1^H NMR (400 MHz, DMSO-*d*_6_) δ 12.88 (s, 1H, quinazolone-N*H*), 9.38 (d, *J* = 6.6 Hz, 2H, pyridine-2-*H*, pyridine-6-*H*), 8.69 (s, 1H, C=C*H*), 8.49 (d, *J* = 6.6 Hz, 2H, pyridine-3-*H*, pyridine-5-*H*), 8.20 (d, *J* = 7.1 Hz, 1H, quinazolone-5-*H*), 7.93 (t, *J* = 8.3 Hz, 1H, quinazolone-7-*H*), 7.81 (d, *J* = 8.1 Hz, 1H, quinazolone-8-*H*), 7.75 -7.71 (m, 2H, Ph-3-*H*, Ph-5-*H*), 7.66 (t, *J* = 7.7 Hz, 1H, quinazolone-6-*H*), 7.37–7.33 (m, 2H, Ph-2-*H*, Ph-6-*H*), 5.95 (s, 2H, C*H*_2_-Ph) ppm. ^13^C NMR (101 MHz, DMSO-*d*_6_) δ 164.4, 162.0, 148.4, 148.2, 148.0, 146.0, 143.7, 135.7, 132.4, 132.3, 130.6, 130.5, 129.0, 128.5, 128.1, 126.6, 122.0, 116.8, 116.6, 116.0, 114.6, 63.0 ppm. HRMS (ESI) calcd. for C_23_H_16_FN_4_O^+^Br^−^ [M − Br^−^]^+^, 383.1303; found, 383.1302.

#### 3.2.27. Synthesis of *(E)*-1-(4-Chlorobenzyl)-4-(2-cyano-2-(4-oxo-3,4-dihydroquinazolin-2-yl)vinyl)pyridin-1-ium (**13e**)

Compound **13e** was prepared according to the procedure described for compound **13a**, starting from compound **9c** (50 mg, 0.18 mmol) and *p*-chlorobenzyl bromide (75 mg, 0.36 mmol). The target compound **13e** was obtained as yellow solid (80 mg). Yield: 91.5%, M.p.: 226–228 °C. ^1^H NMR (400 MHz, DMSO-*d*_6_) δ 12.88 (s, 1H, quinazolone-N*H*), 9.37 (d, *J* = 6.7 Hz, 2H, pyridine-2-*H*, pyridine-6-*H*), 8.69 (s, 1H, C=C*H*), 8.49 (d, *J* = 6.4 Hz, 2H, pyridine-3-*H*, pyridine-5-*H*), 8.20 (d, *J* = 8.9 Hz, 1H, quinazolone-5-*H*), 7.93 (t, *J* = 7.0 Hz, 1H, quinazolone-7-*H*), 7.82 (d, *J* = 7.9 Hz, 1H, quinazolone-8-*H*), 7.70–7.63 (m, 3H, Ph-3-*H*, Ph-5-*H*, quinazolone-6-*H*), 7.58 (d, *J* = 8.5 Hz, 2H, Ph-2-*H*, Ph-6-*H*), 5.96 (s, 2H, C*H*_2_-Ph) ppm. ^13^C NMR (101 MHz, DMSO-*d*_6_) δ 161.9, 148.4, 148.2, 148.0, 146.2, 143.7, 135.7, 134.9, 133.2, 131.8, 129.8, 129.0, 128.5, 128.1, 126.6, 122.0, 116.0, 114.6, 63.0 ppm. HRMS (ESI) calcd. for C_23_H_16_ClN_4_O^+^Br^−^ [M − Br^−^]^+^, 399.1007; found, 399.1004.

#### 3.2.28. Synthesis of *(E)*-4-(2-Cyano-2-(4-oxo-3,4-dihydroquinazolin-2-yl)vinyl)-1-(4-(trifluoromethyl)benzyl) pyridin-1-ium (**13f**)

Compound **13f** was prepared according to the procedure described for compound **13a**, starting from compound **9c** (50 mg, 0.18 mmol) and *p*-trifluoromethylbenzyl bromide (85 mg, 0.36 mmol). The target compound **13f** was obtained as yellow solid (77 mg). Yield: 82.3%, M.p.: 221–223 °C. ^1^H NMR (400 MHz, DMSO-*d*_6_) δ 12.87 (s, 1H, quinazolone-N*H*), 9.39–9.38 (m, 2H, pyridine-2-*H*, pyridine-6-*H*), 8.69 (s, 1H, C=C*H*), 8.51 (d, *J* = 6.8 Hz, 2H, pyridine-3-*H*, pyridine-5-*H*), 8.20 (d, *J* = 9.2 Hz, 1H, quinazolone-5-*H*), 7.95–7.90 (m, 1H, quinazolone-7-*H*), 7.88 (d, *J* = 8.4 Hz, 2H, Ph-3-*H*, Ph-5-*H*), 7.82 (m, 3H, quinazolone-8-*H*, Ph-2-*H*, Ph-6-*H*)), 7.66 (t, *J* = 8.1 Hz, 1H, quinazolone-6-*H*), 6.07 (s, 2H, C*H*_2_-Ph) ppm. ^13^C NMR (101 MHz, DMSO-*d*_6_) δ 161.9, 148.6, 148.2, 148.0, 146.4, 143.6, 138.7, 135.7, 134.5, 130.5, 130.2, 129.0, 128.5, 128.2, 126.6, 125.8, 122.1, 116.1, 114.6, 63.1 ppm. HRMS (ESI) calcd. for C_24_H_16_F_3_N_4_O^+^Br^−^ [M − Br^−^]^+^, 433.1271; found, 433.1271.

#### 3.2.29. Synthesis of *(E)*-4-(2-Cyano-2-(4-oxo-3,4-dihydroquinazolin-2-yl)vinyl)-1-(4-nitrobenzyl)pyridin-1-ium (**13g**)

Compound **13g** was prepared according to the procedure described for compound **13a**, starting from compound **9c** (50 mg, 0.18 mmol) and *p*-nitrobenzyl bromide (80 mg, 0.36 mmol). The target compound **13g** was obtained as yellow solid (88 mg). Yield: 98.5%, M.p.: 237–239 °C. ^1^H NMR (400 MHz, DMSO-*d*_6_) δ 12.88 (s, 1H, quinazolone-N*H*), 9.42–9.40 (m, 2H, pyridine-2-*H*, pyridine-6-*H*), 8.70 (s, 1H, C=C*H*), 8.52 (d, *J* = 6.7 Hz, 2H, pyridine-3-*H*, pyridine-5-*H*), 8.38–8.30 (m, 2H, Ph-3-*H*, Ph-5-*H*), 8.21 (d, *J* = 9.0 Hz, 1H, quinazolone-5-*H*), 7.96–7.90 (m, 1H, quinazolone-7-*H*), 7.87 (d, *J* = 8.8 Hz, 2H, Ph-2-*H*, Ph-6-*H*), 7.82 (d, *J* = 7.8 Hz, 1H, quinazolone-8-*H*), 7.66 (t, *J* = 7.1 Hz, 1H, quinazolone-6-*H*), 6.14 (s, 2H, C*H*_2_-Ph) ppm. ^13^C NMR (101 MHz, DMSO-*d*_6_) δ 161.9, 148.7, 148.5, 148.0, 146.5, 143.6, 141.2, 135.7, 131.0, 130.6, 129.0, 128.5, 128.2, 126.6, 126.0, 124.7, 122.0, 116.1, 114.6, 62.8 ppm. HRMS (ESI) calcd. for C_23_H_16_N_5_O_3_^+^Br^−^ [M − Br^−^]^+^, 410.1248; found, 410.1248.

#### 3.2.30. Synthesis of *(E)*-4-(2-Cyano-2-(4-oxo-3,4-dihydroquinazolin-2-yl)vinyl)-1-(4-cyanobenzyl)pyridin-1-ium (**13h**)

Compound **13h** was prepared according to the procedure described for compound **13a**, starting from compound **9c** (50 mg, 0.18 mmol) and *p*-cyanobenzyl bromide (71 mg, 0.36 mmol). The target compound **13h** was obtained as yellow solid (76 mg). Yield: 88.6%, M.p.: 236–238 °C. ^1^H NMR (400 MHz, DMSO-*d*_6_) δ 12.88 (s, 1H, quinazolone-N*H*), 9.40–9.38 (m, 2H, pyridine-2-*H*, pyridine-6-*H*), 8.70 (s, 1H, C=C*H*), 8.51 (d, *J* = 6.8 Hz, 2H, pyridine-3-*H*, pyridine-5-*H*), 8.20 (d, *J* = 8.0 Hz, 1H, quinazolone-5-*H*), 7.99 (d, *J* = 8.4 Hz, 2H, Ph-3-*H*, Ph-5-*H*), 7.95–7.91 (m, 1H, quinazolone-7-*H*), 7.83–7.80 (m, 3H, quinazolone-8-*H*, Ph-2-*H*, Ph-6-*H*), 7.66 (t, *J* = 7.6 Hz, 1H, quinazolone-6-*H*), 6.07 (s, 2H, C*H*_2_-Ph) ppm. ^13^C NMR (101 MHz, DMSO-*d*_6_) δ 161.9, 148.6, 148.2, 148.0, 146.4, 143.6, 139.4, 135.7, 133.6, 130.5, 129.0, 128.4, 128.2, 126.6, 122.0, 118.8, 116.1, 114.6, 112.7, 63.1 ppm. HRMS (ESI) calcd. for C_24_H_16_N_5_O ^+^Br^−^ [M − Br^−^]^+^, 390.1349; found, 390.1349.

#### 3.2.31. Synthesis of *(E)*-4-(2-Cyano-2-(4-oxo-3,4-dihydroquinazolin-2-yl)vinyl)-1-(2,4- difluorobenzyl)pyridin-1-ium (**13i**)

Compound **13i** was prepared according to the procedure described for compound **13a**, starting from compound **9c** (50 mg, 0.18 mmol) and 2,4-difluorobenzyl bromide (76 mg, 0.36 mmol). The target compound **13i** was obtained as yellow solid (84 mg). Yield: 95.6%, M.p.: 238–240 °C. ^1^H NMR (400 MHz, DMSO-*d*_6_) δ 12.87 (s, 1H, quinazolone-N*H*), 9.32 (d, *J* = 6.3 Hz, 2H, pyridine-2-*H*, pyridine-6-*H*), 8.70 (s, 1H, C=C*H*), 8.50 (d, *J* = 6.4 Hz, 2H, pyridine-3-*H*, pyridine-5-*H*), 8.20 (d, *J* = 7.8 Hz, 1H, quinazolone-5-*H*), 7.93 (t, *J* = 7.6 Hz, 1H, quinazolone-7-*H*), 7.86–7.81 (m, 2H, quinazolone-8-*H*, Ph-6-*H*), 7.66 (t, *J* = 7.5 Hz, 1H, quinazolone-6-*H*), 7.46 (t, *J* = 8.7 Hz, 1H, Ph-5-*H*), 7.29 (t, *J* = 7.2 Hz, 1H, Ph-3-*H*), 6.04 (s, 2H, C*H*_2_-Ph) ppm. ^13^C NMR (101 MHz, DMSO-*d*_6_) δ 165.1, 165.0, 162.9, 162.8, 162.7, 162.5, 161.6, 160.4, 160.3, 148.6, 148.2, 148.0, 146.3, 143.6, 135.7, 134.1, 134.0, 134.0, 133.9, 129.0, 128.5, 128.1, 127.3, 126.6, 122.1, 117.9, 117.8, 117.7, 117.7, 116.0, 114.6, 113.1, 113.1, 112.9, 112.9, 105.6, 105.3, 105.1, 57.9 ppm. HRMS (ESI) calcd. for C_23_H_15_F_2_N_4_O^+^Br^−^ [M − Br^−^]^+^, 401.1208; found, 401.1208.

#### 3.2.32. Synthesis of Intermediates **15a**–**e**

Intermediate **15** was prepared through the reported methods described in reference [77].

#### 3.2.33. Synthesis of *(E)*-4-(2-cyano-2-(7-fluoro-4-oxo-3,4-dihydroquinazolin-2-yl)vinyl)-1-(4-methylbenzyl) pyridin-1-ium (**19a**)

Compound **19a** was prepared according to the procedure described for compound **13a**, starting from compound **18a** (50 mg, 0.17 mmol) and *p*-methylbenzyl bromide (60 mg, 0.34 mmol). The target compound **19a** was obtained as yellow solid (50 mg). Yield: 55.0%, M.p.: 260–262 °C. ^1^H NMR (600 MHz, DMSO) δ 12.95 (s, 1H, quinazolone-N*H*), 9.38 (d, *J* = 6.4 Hz, 2H, pyridine-2-*H*, pyridine-6-*H*), 8.70 (s, 1H, C=C*H*), 8.48 (d, *J* = 6.3 Hz, 2H, pyridine-3-*H*, pyridine-5-*H*), 8.27–8.25 (m, 1H, quinazolone-5-*H*), 7.62 (d, *J* = 8.9Hz, 1H, quinazolone-8-*H*), 7.52 (m, 3H, quinazolone-6-*H*, Ph-2-*H*, Ph-6-*H*), 7.30 (d, *J* = 7.8 Hz, 2H, Ph-3-*H*, Ph-5-*H*), 5.92 (s, 2H, C*H*_2_-Ph), 2.33(s, 3H, Ph-C*H*_3_) ppm. ^13^C NMR (151 MHz, DMSO-*d*_6_) δ 166.4 (d, *J* = 252 Hz, ^1^*J*_CF_, quinazolone-7-*C*), 161.2 (quinazolone-4-*C*O), 150.1 (quinazolone-2-*C*), 149.6 (quinazolone-9-*C*), 148.2 (pyridine-4-*C*), 146.0 (pyridine-2,6-*C*), 144. 5 (CN-C=*C*H), 139.7 (Ph-4-*C*), 131.4 (Ph-1-*C*), 130.3 (Ph-3,5-2*C*), 129.7 (d, *J* = 11 Hz, ^3^*J*_CF_, quinazolone-5-*C*), 129.7 (Ph-2,6-2*C*), 128.1 (pyridine-3,5-*C*), 119.1 (quinazolone-10-*C*), 117.4 (d, *J* = 23 Hz, ^2^*J*_CF_, quinazolone-6-*C*), 115.7 (*C*N-C=CH), 114.5 (CN-*C*=CH), 113.7 (d, *J* = 23 Hz, ^2^*J*_CF_, quinazolone-8-*C*), 63.7 (*C*H_2_-Ph), 21.3 (*C*H_3_) ppm. HRMS (ESI) calcd. for C_24_H_18_FN_4_O^+^Br^−^ [M − Br^−^]^+^, 397.1459; found, 397.1460.

#### 3.2.34. Synthesis of *(E)*-4-(2-(7-Chloro-4-oxo-3,4-dihydroquinazolin-2-yl)-2-cyanovinyl)-1-(4- methylbenzyl)pyridin-1-ium (**19b**)

Compound **19b** was prepared according to the procedure described for compound **13a**, starting from compound **18b** (50 mg, 0.16 mmol) and *p*-methylbenzyl bromide (60 mg, 0.32 mmol). The target compound **19a** was obtained as yellow solid (54 mg). Yield: 60.9%, M.p.: 270–272 °C. ^1^H NMR (600 MHz, DMSO-*d*_6_) δ 12.87 (s, 1H, quinazolone-N*H*), 9.34 (d, *J* = 5.6 Hz, 2H, pyridine-2-*H*, pyridine-6-*H*), 8.67 (s, 1H, C=C*H*), 8.47 (d, *J* = 5.6 Hz, 2H, pyridine-3-*H*, pyridine-5-*H*), 8.17–8.14 (m, 1H, quinazolone-5-*H*), 7.68 (d, *J* = 7.9 Hz, 1H, quinazolone-8-*H*), 7.63 (d, *J* = 7.8 Hz, 1H, quinazolone-6-*H*), 7.50 (d, *J* = 7.4 Hz, 2H, Ph-3-*H*, Ph-5-*H*), 7.30 (d, *J* = 7.1 Hz, 2H, Ph-3-*H*, Ph-5-*H*), 5.89 (s, 2H, C*H*_2_-Ph), 2.33 (s, 3H, Ph-C*H*_3_) ppm.

#### 3.2.35. Synthesis of *(E)*-4-(2-Cyano-2-(6,8-dichloro-4-oxo-3,4-dihydroquinazolin-2-yl)vinyl)-1-(4- methylbenzyl)pyridin-1-ium (**19c**)

Compound **19c** was prepared according to the procedure described for compound **13a**, starting from compound **18c** (50 mg, 0.15 mmol) and *p*-methylbenzyl bromide (54 mg, 0.30 mmol). The target compound **19c** was obtained as yellow solid (58 mg). Yield: 68.9%, M.p.: 264–266 °C. ^1^H NMR (600 MHz, DMSO-*d*_6_) δ 13.23 (s, 1H), 9.34 (d, *J* = 5.9 Hz, 2H, pyridine-2-*H*, pyridine-6-*H*), 8.68 (s, 1H, C=C*H*), 8.47 (d, *J* = 5.9 Hz, 2H, pyridine-3-*H*, pyridine-5-*H*), 8.24 (s, 1H, quinazolone-7-*H*), 8.10 (s, 1H, quinazolone-5-*H*), 7.50 (d, *J* = 7.4 Hz, 2H, Ph-2-*H*, Ph-6-*H*), 7.30 (d, *J* = 7.3 Hz, 2H, Ph-3-*H*, Ph-5-*H*), 5.89 (s, 2H, C*H*_2_-Ph), 2.33 (s, 3H, Ph-C*H*_3_) ppm.

#### 3.2.36. Synthesis of *(E)*-4-(2-Cyano-2-(6-methyl-4-oxo-3,4-dihydroquinazolin-2-yl)vinyl)-1-(4- methylbenzyl)pyridin-1-ium (**19d**)

Compound **19d** was prepared according to the procedure described for compound **13a**, starting from compound **18d** (50 mg, 0.17 mmol) and *p*-methylbenzyl bromide (64 mg, 0.34 mmol). The target compound **19d** was obtained as yellow solid (88 mg). Yield: 96.2%, M.p.: 248–250 °C. ^1^H NMR (600 MHz, DMSO-*d*_6_) δ 12.83 (s, 1H, quinazolone-N*H*), 9.39 (d, *J* = 6.2 Hz, 2H, pyridine-2-*H*, pyridine-6-*H*), 8.71 (s, 1H, C=C*H*), 8.49 (d, *J* = 6.2 Hz, 2H, pyridine-3-*H*, pyridine-5-*H*), 8.02 (d, *J* = 7.9 Hz, 1H, quinazolone-5-*H*), 7.77 (d, *J* = 7.2 Hz, 1H, quinazolone-7-*H*), 7.56–7.47 (m, 3H, quinazolone-8-*H*, Ph-3-*H*, Ph-5-*H*), 7.30 (d, *J* = 7.6 Hz, 2H, Ph-3-*H*, Ph-5-*H*), 5.93 (s, 2H, C*H*_2_-Ph), 2.61 (s, 3H, quinazolone-C*H*_3_), 2.33 (s, 3H, Ph-C*H*_3_) ppm. ^13^C NMR (151 MHz, DMSO-*d*_6_) δ 162.1, 151.0, 148.3, 146.0, 143.0, 139.7, 136.9, 136.0, 131.4, 130.3, 129.7, 128.0, 124.2, 123.5, 122.0, 116.4, 114.5, 63.7, 21.3, 17.2 ppm.

#### 3.2.37. Synthesis of *(E)*-4-(2-Cyano-2-(8-methyl-4-oxo-3,4-dihydroquinazolin-2-yl)vinyl)-1-(4- methylbenzyl)pyridin-1-ium (**19e**)

Compound **19e** was prepared according to the procedure described for compound **13a**, starting from compound **18e** (50 mg, 0.17 mmol) and *p*-methylbenzyl bromide (64 mg, 0.34 mmol). The target compound **19e** was obtained as yellow solid (62 mg). Yield: 72.9%, M.p.: 252–254 °C. ^1^H NMR (600 MHz, DMSO-*d*_6_) δ 12.83 (s, 1H, quinazolone-N*H*), 9.40 (d, *J* = 6.4 Hz, 2H, pyridine-2-*H*, pyridine-6-*H*), 8.71 (s, 1H, C=C*H*), 8.49 (d, *J* = 6.4 Hz, 2H, pyridine-3-*H*, pyridine-5-*H*), 8.02 (d, *J* = 7.7 Hz, 1H, quinazolone-5-*H*), 7.77 (d, *J* = 7.2 Hz, 1H, quinazolone-7-*H*), 7.53 (t, *J* = 7.5 Hz, 3H, quinazolone-6-*H*, Ph-3-*H*, Ph-5-*H*), 7.30 (d, *J* = 7.8 Hz, 2H, Ph-3-*H*, Ph-5-*H*), 5.93 (s, 2H, C*H*_2_-Ph), 2.61 (s, 3H, quinazolone-C*H*_3_), 2.33 (s, 3H, Ph-C*H*_3_) ppm. ^13^C NMR (151 MHz, DMSO-*d*_6_) δ 162.1, 151.1, 148.3, 146.0, 143.0, 139.7, 136.9, 136.0, 131.4, 130.3, 129.7, 128.5, 128.0, 124.2, 123.4, 122.0, 116.4, 114.5, 63.7, 21.3, 17.2 ppm.

### 3.3. Biological Assay

#### 3.3.1. Antibacterial Evaluation

Minimal inhibitory concentration (MIC, μg/mL) is defined as the lowest concentration of target compounds that completely inhibit the growth of bacteria, by means of the standard twofold serial dilution method in 96-well microtest plates according to the Clinical and Laboratory Standards Institute (CLSI). Norfloxacin was used as a reference drug. No compound in DMSO with bacteria inoculation was used as a negative control to ensure that the solvent had no effect on bacteria growth. All the bacteria growth was monitored visually, and the experiments were performed in triplicate.

The target molecules were evaluated for their antibacterial activities against five Gram-positive bacteria (Methicillin-resistant *Staphylococcus aureus* N315, *Staphylococcus aureus*, *Staphylococcus aureus* ATCC 25923, *Staphylococcus aureus* ATCC 29213, *Enterococcus faecalis*) and six Gram-negative bacteria (*Klebsiella pneumonia*, *Escherichia coli*, *Escherichia coli* ATCC 25922, *Pseudomonas aeruginosa*, *Pseudomonas aeruginosa* ATCC 27853, *Acinetobacter baumannii*). The compounds were dissolved in DMSO to prepare the stock solutions. The compounds and reference drugs were prepared in Mueller–Hinton broth (Guangdong huaikai microbial sci.& tech Co., Ltd., Guangzhou, Guangdong, China) by twofold serial dilution to obtain the required concentrations of 256, 128, 64, 32, 16, 8, 4, 2, 1, 0.5, and 0.25 μg/mL. These dilutions were inoculated and incubated at 37 °C for 24 h.

#### 3.3.2. Hemolytic Toxicity

The human red blood cells (RBCs) were collected and washed three times with saline. Subsequently, the cells were reallocated in saline to provide 5% *V*/*V* red blood cells suspension. The suspension (100 μL) was added to a 96-well plate containing 100 μL of fourfold serially diluted quinazolone pyridinium **19a** solution and incubated for 1 h at 37 °C. The mixture was centrifuged at 2000 rpm for 5 min, and the supernatant (100 μL) was removed to a new 96-well plate. The absorbance of the supernatant was measured at 540 nm using a microplate reader. Distilled water and Triton X-100 were used as positive and negative controls, respectively. The hemolytic activity was calculated by the following equation: hemolysis (%) = [(OD_sample_−OD_saline_)/(OD _Triton X-100_−OD_saline_)] × 100%.

#### 3.3.3. Drug-Resistance Assay

The propensity of drug resistance induced by quinazolone pyridinium **19a** for MRSA and *E. coli* was evaluated by the sequential passaging method, and the MIC values were tested as described in biological assays procedures. After the MIC value was determined in the first generation, half-MIC suspension of resistant *E. coli* cells was taken for subculture, and every MIC value was obtained after 12 h incubation with quinazolone pyridinium **19a**. The process lasted for 20 days.

#### 3.3.4. Inhibition of Bacterial Growth

MRSA and *E. coli* culture (10^7^ CFU/mL) was respectively treated with quinazolone pyridinium **19a** (1× and 4 × MIC), norfloxacin (1 × MIC) in a 96-well plate at 37 °C. At different intervals (0, 1, 2, 3, 4, 5 and 6 h), 20 μL bacteria solution were separately removed and 10-fold serially diluted in sterile phosphate-buffered saline (PBS). Each dilution (100 μL) was plated onto the Mueller–Hinton agar plates and incubated at 37 °C for 24 h. Finally, the number of bacterial colony growth on plates was counted, and CFU per mL was calculated.

#### 3.3.5. Inhibition of Biofilm

Aliquots of 100 μL MRSA and *E. coli* bacteria suspensions (an OD_600_ around 0.1) and quinazolone pyridinium **19a** at the different concentrations of 0, 0.5, 1. 2, 4, and 8 × MIC were co-incubated in a 96-well plate for 24 h at 37 °C. The suspension was removed from the wells, and the biofilms were washed twice with PBS carefully to remove planktonic cells. The 0.1% crystal violet was added to each well for 30 min to stain the biofilm. After crystal violet was discarded, the biofilm samples were washed with phosphate buffer saline and dried, the absorbance at 600 nm was measured using a microplate reader (Tecan Infinite M200 Pro). The % viability of the treated biofilms concerning the control was calculated and plotted. Dimethyl sulfoxide was used as a negative control, and all samples were conducted in triplicate.

#### 3.3.6. Membrane Depolarization 

The pre-cultured MRSA and *E. coli* strain were harvested (6000 rpm, 5 min), washed, and resuspended with a mixed buffer (5 mM HEPES/5 mM glucose/100 mM KCl solution, 1/1/1, V/V/V). The bacterial suspensions (~10^5^ CFU/mL, 100 μL) were added to the wells and followed by diSC_3_5 dye (10 μM, 50 μL) and EDTA (200 μM, 50 μL), and the mixture were preincubated for about 30 min in dark. After cultivation, bacterial suspensions were removed, and wells were washed with PBS. Then the bacteria were dosed with active quinazolone pyridinium **19a** (0.5, 1. 2, 4, and 8 × MIC), and the fluorescence spectrum was recorded on fluorescence photometer set to an excitation wavelength of 622 nm and an emission wavelength of 670 nm. DMSO was used as a negative control, and all samples were conducted in triplicate.

#### 3.3.7. Assay for Outer Membrane Permeability

The MRSA and *E. coli* train was treated similarly as mentioned in earlier experiments, followed by the addition of *N*-phenylnaphthylamine dye (10 μM, 50 μL) and pre-incubation for about 30 min. After cultivation, the bacterial suspensions were removed, and wells were washed with PBS. Then the bacteria were dosed with active quinazolone pyridinium **19a** (0.5, 1. 2, 4, and 8 × MIC), and the fluorescence spectrum was recorded on fluorescence photometer set to an excitation wavelength of 350 nm and an emission wavelength of 420 nm. DMSO was used as a negative control, and all samples were conducted in triplicate

#### 3.3.8. Assay for Inner Membrane Damage

The MRSA and *E. coli* strain was treated similarly as mentioned in earlier experiments, followed by the addition of PI (10 μM, 50 μL) and pre-incubation for 30 min at 37 °C. After cultivation, the bacterial suspensions were removed, and wells were washed with PBS. Then the bacteria were dosed with active quinazolone pyridinium **19a** (0.5, 1. 2, 4, and 8 × MIC), and the fluorescence spectrum was recorded on fluorescence photometer set toan excitation wavelength of 535 nm and an emission wavelength of 617 nm. DMSO was used as a negative control, and all samples were conducted in triplicate.

#### 3.3.9. Leakage of Cellular Protein

A range of concentrations of quinazolone pyridinium **19a** (0.5, 1, 2, 4, and 8 × MIC) were incubated with MRSA and *E. coli* (~10^5^ CFU/mL). The bacterial suspension was added to an equal volume of 0.01 M phosphate buffer saline as a control. After the cultivation at 37 °C for 18 h, the bacterial supernatant (6000 rpm, 5 min)were collected. The concentration of leaked proteins in the supernatant was measured using a standard Bradford assay. DMSO was used as a negative control, and all samples were conducted in triplicate.

#### 3.3.10. Assessment of Metabolic Inactivation

The MRSA and *E. coli* suspensions (~10^5^ CFU/mL) were treated with a range of concentrations of quinazolone pyridinium **19a** for 6 h at 37 °C. Alamar blue (50 μg/mL, 25 μL) was added to incubate with both control and treated cells for 1 h at 37 °C, then well contents were measured at 570 nm on a Thermo Scientific Microplate Reader (Thermo Fisher Scientific, Waltham, MA, USA). The average % reduction was used to determine metabolic activity. Dimethyl sulfoxide was used as a negative control, and all samples were conducted in triplicate.

#### 3.3.11. Reactive Oxygen Species (ROS) Production

The MRSA and *E. coli* cells (~10^5^ CFU/mL) were treated with quinazolone pyridinium **19a** (0.5, 1, 2, 4, and 8 × MIC) at 37 °C for 18 h, then DCFH-DA dye (10 μM, 30 μL) was added to incubate with bacterial suspensions at 37 °C for 30 min in dark. After cultivation, the bacterial suspensions were removed, and wells were washed with PBS. Fluorescence was measured at an excitation wavelength of 488 nm and an emission wavelength of 522 nm. DMSO was used as a negative control, and all samples were conducted in triplicate.

#### 3.3.12. Absorption Spectra of DNA

UV spectra were recorded at room temperature on a TU-2450 spectrophotometer (Puxi Analytic Instrument Ltd. of Beijing, China) equipped with 1.0 cm quartz cells. The stock solution of quinazolone pyridinium **19a** was prepared in DMF. Tris-HCl buffer solution (pH = 7.4) was prepared by mixing and diluting Tris (tris(hydroxymethyl)aminomethane) solution with HCl solution. Tris and HCl were analytical purity. DNA intercalation assay was carried out using calf thymus DNA as a substrate. Quinazolone pyridinium **19a** kept adding to the calf thymus DNA solution (c(DNA) = 1.56 × 10^−4^ mol/L) to make the final concentration of quinazolone pyridinium **19a** ranging 0 to 2.67 × 10^−5^ mol/L

#### 3.3.13. AO Binding Assay

AO was added to the calf thymus DNA solution (c(DNA) = 1.56 × 10^−4^ mol/L). Then, quinazolone pyridinium**19a** kept adding to the above solution to make the final concentration of quinazolone pyridinium **19a** ranging 0–2.67 × 10^−5^ mol/L. The fluorescence spectral was recorded under each gradient.

#### 3.3.14. Measurement of PBP2a Contents

The 10^6^ CFU/mL of MRSA were treated with corresponding compounds for 6 h at 37 °C and 200 rpm. Following treatment, the cells were centrifuged at 5000 rpm for 5 min, and the supernatant was discarded. The PBP2a contents in control and treated cells were measured using the PBP2a Elisa Assay Kit (Nanjing Herb Source Bio-Technology Co., Ltd., Nanjing, China).

To investigate the binding of quinazolone pyridinium **19a** to the allosteric site of PBP2a, the transpeptidase active site of purified PBP2a was irreversibly acylated by incubation with an excess amount of oxacillin for 45 min at room temperature. Unbound oxacillin was re-moved with a protein desalting column (Pierce), and the acylated protein was used in subsequent experiments. Binding of quinazolone pyridinium **19a** to the allosteric site was determined by monitoring the intrinsic fluorescence of tryptophan and tyrosine residues of PBP2a. Using the fluorescence spectrofluorometer, PBP2a (500 nM) was incubated at room temperature in buffer containing HEPES (pH 7, 25 mM), NaCl (1 M) for 10 min. Upon excitation at 280 nm, the emission spectra of protein alone were taken. Quinazolone pyridinium **19a** was titrated into the reaction, and the emission spectra were measured. The decrease in fluorescent emission at 330 nm for each concentration of quinazolone pyridinium **19a** was quantified. The normalized difference in fluorescence was plotted versus concentration. All the experiments were done at least in triplicate.

#### 3.3.15. Interaction with PBP2a

Crystal structure of PBP2a (PDB code: 4CJN) in PBD format was downloaded from the Protein Data Bank. AutoDock 4.2.6 was used to perform docking of the highly active quinazolone pyridinium **19a** with the ligand molecule. Discovery Studio 4.5 Client (2D) was used to visualize the molecular interactions.

## 4. Conclusions

A series of novel structural heteroarylcyanovinyl quinazolones and quinazolone pyridiniums were designed and synthesized, and most of the target molecules exhibited potent antibacterial activity against Gram-positive and Gram-negative bacteria. SAR studies demonstrated that the introduction of pyridiniums was beneficial to broaden the antibacterial spectrum, and the position, number, and type of substituents on quinazolone also played important roles in affecting the antibacterial activity. Quinazolone pyridinium **19a** showed stronger antibacterial activity than norfloxacin against MRSA and *E. coli* with extremely low MICs of 0.5 μg/mL, as well as no obvious resistance and hemolytic toxicity and good inhibition of bacterial biofilm. Further mechanistic studies revealed that the highly active compound **19a** with a unique quaternary ammonium exerted its antibacterial activity through the multitargeting actions of destroying bacterial cell membranes, intercalating into the bacterial DNA and decreasing the metabolic activity. Moreover, active molecule **19a** could enhance the anti-MRSA activity of *β*-lactam drug as an allosteric modulator of PBP2a. These significant findings indicated that quinazolone pyridinium **19a** can be exploited as a promising candidate for the treatment of infections with multidrug-resistant bacteria, specifically MRSA infections.

## Figures and Tables

**Table 1:** In vitro antibacterial data (MIC, μg/mL) for cyanovinyl quinazolone derivatives **3**–**19**.

Compds.	Gram-Positive Bacteria ^a^						Gram-Negative Bacteria ^b^				
	MRSA	*S. a.*	*S. a.* 25923	*S. a.* 29213	*E. f.*	*K. p.*	*E. c.*	*E. c.* 25922	*P. a.*	*P. a.* 27853	*A. b.*
**4a**	4	16	8	32	64	64	2	16	32	16	64
**4b**	8	16	16	16	4	128	2	2	4	128	64
**4c**	8	32	32	32	16	128	4	4	8	32	128
**5a**	16	4	1	32	64	8	8	2	1	64	16
**5b**	16	32	4	16	8	32	32	4	8	8	8
**6a**	8	64	32	16	32	32	4	16	8	16	16
**6b**	16	64	8	16	128	128	1	16	1	32	16
**6c**	32	8	1	16	128	64	4	16	4	32	8
**7**	64	64	32	16	128	128	128	8	16	128	32
**8a**	16	32	32	8	64	32	2	4	8	128	32
**8b**	8	8	4	8	2	8	4	4	2	4	128
**9a**	8	4	32	8	4	128	16	8	16	256	64
**9b**	2	16	32	16	2	256	8	16	16	256	64
**9c**	4	4	32	1	4	256	1	4	1	64	64
**10**	256	128	2	16	16	8	8	16	2	16	16
**11a**	32	16	4	2	64	32	16	32	1	128	32
**11b**	8	64	1	32	64	16	32	16	32	16	32
**12a**	8	32	16	128	2	32	32	32	4	16	16
**12b**	16	16	64	16	128	8	64	4	64	32	16
**12c**	4	32	32	32	64	16	128	16	16	64	32
**13a**	2	8	64	64	128	32	64	32	16	64	128
**13b**	1	4	32	16	8	8	0.5	32	2	16	8
**13c**	8	8	16	16	8	4	16	1	16	16	64
**13d**	64	8	64	2	4	16	32	128	32	8	128
**13e**	32	16	64	32	64	64	8	8	64	32	128
**13f**	64	4	128	64	32	32	32	32	4	32	64
**13g**	32	8	8	16	8	32	4	32	2	16	8
**13h**	64	128	64	16	4	32	4	16	8	64	32
**13i**	128	16	64	128	8	64	8	128	16	32	64
**19a**	0.5	1	1	2	2	2	0.5	8	2	4	2
**19b**	4	4	8	16	16	4	16	16	4	8	4
**19c**	8	8	4	16	4	16	8	16	4	16	2
**19d**	8	4	4	8	4	2	4	16	0.5	4	4
**19e**	16	8	8	4	8	8	8	8	4	16	8
Norfloxacin	4	8	4	2	2	4	4	2	4	8	2

^a^ MRSA, Methicillin-resistant *Staphylococcus aureus* (N315); *S. a.*, *Staphylococcus* aureus; S. a. 25923, *Staphylococcus aureus* ATCC 25923; *S. a.* 29213, *Staphylococcus aureus* ATCC 29213; *E. f.*, *Enterococcus faecalis*; ^b^ *K. p., Klebsiella pneumonia*; *E. c., Escherichia coli*; *E. c*. 25922, *Escherichia coli* ATCC 25922; *P. a., Pseudomonas aeruginosa*; *P. a*. 27853, *Pseudomonas aeruginosa* ATCC 27853; *A. b., Acinetobacter baumanii*.

**Table 2:** Contents of PBP2a in MRSA of quinazolone pyridinium **19a** and cefdinir on MRSA.

Compounds	PBP2a Contents in Treated MRSA (ng/mL)
Control	17.19 ± 0.76
Compound **19a**	8.46 ± 0.18
Cefdinir	10.48 ± 0.12
Compound **19a** + Cefdinir	3.79 ± 0.59

## Data Availability

The original contributions presented in this study are included in the article/supplementary material. Further inquiries can be directed to the corresponding author.

## Supplementary Materials

The following supporting information can be downloaded at: https://www.mdpi.com/article/10.3390/molecules30020243/s1, Figure S1: Picture of hemolytic rates of compound **19a**; Figure S2: The plot of *A*^0^/(*A-A*^0^) versus 1/[compound **19a**], yielding the binding constant, *K* = 1.01 × 10^5^ L/mol, R = 0.996, SD = 0.039 (R is the correlation coefficient; SD is standard deviation), spectra of compounds.
