# Supplementary material for: Discovery of Quinazolone Pyridiniums as Potential Broad-Spectrum Antibacterial Agents
Source: Molecules. 2025 Jan 9;30(2):243. doi: 10.3390/molecules30020243 (PMC11767251; doi:10.3390/molecules30020243)

Supplementary Information

Discovery of quinazolone pyridiniums as potential broad-spectrum antibacterial agents

Author Names and Affiliations:

Jie Dai [1,†], Qian-Yue Li [1,†], Zi-Yi Li [1,†], Zhong-Lin Zang [1,*], Yan Luo [2,*] and Cheng-He Zhou [1,*]

[1] *Institute of Bioorganic & Medicinal Chemistry, Key Laboratory of Applied Chemistry of Chongqing Municipality, School of Chemistry and Chemical Engineering, Southwest University, Chongqing 400715, China.*

[2] *College of Pharmacy, National & Local Joint Engineering Research Center of Targeted and Innovative Therapeutics, Chongqing Key Laboratory of Kinase Modulators as Innovative Medicine, Chongqing University of Arts and Sciences, Chongqing, 402160, China*

[†]These authors contributed equally to this work

*Corresponding authors:

Tel.: +86-23-68254967; Fax: +86-23-68254967, E-mail: zhouch@swu.edu.cn (Cheng-He Zhou); lygytha456@163.com (Yan Luo)

1. Hemolytic activity

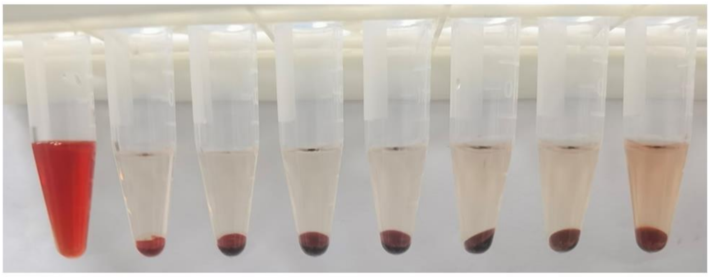

Trition X-100 4× 8× 16× 32× 64× 128× 256×MIC

Fig. S1. Picture of hemolytic rates of compound **19a**

2. Interactions of compound 19a with calf thymus DNA

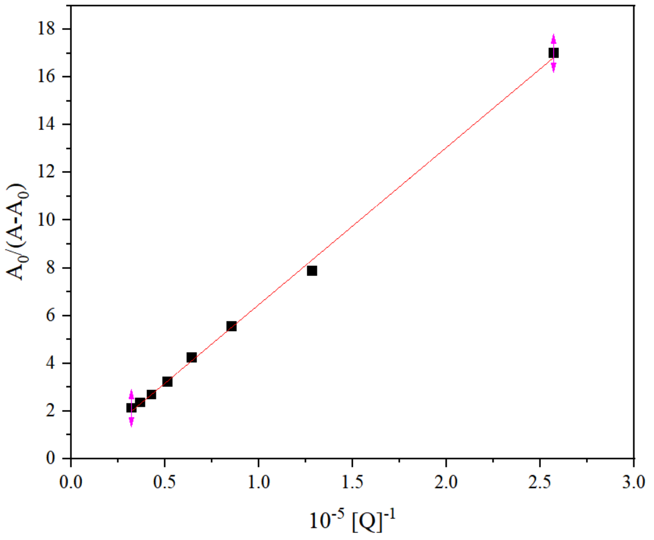

Fig. S2. The plot of $A^0 / (A\text{-}A^0)$ versus 1/[compound **19a**], yielding the binding constant, $K = 1.01 \times 10^5$ L/mol, R = 0.996, SD = 0.039 (R

is the correlation coefficient. SD is standard deviation).

Equation (1) is utilized to calculate the binding constant (K). Where A^0 and A are the absorbance of DNA in the absence and presence of compound **19a** at 260 nm, ξ_C and $\xi_{D\text{-}C}$ represent the absorption coefficients of compound **19a** and compound **19a** -DNA complex respectively (**Fig. S2**).

$$\frac{A^0}{A-A^0}=\frac{\xi_C}{\xi_{D-C}-\xi_C}+\frac{\xi_C}{\xi_{D-C}-\xi_C}\times\frac{1}{K[\mathrm{Q}]} \quad (1)$$

3. Characterizations of some representative compounds

12.1 Spectra of compound **4a**

^{1}H NMR spectrum

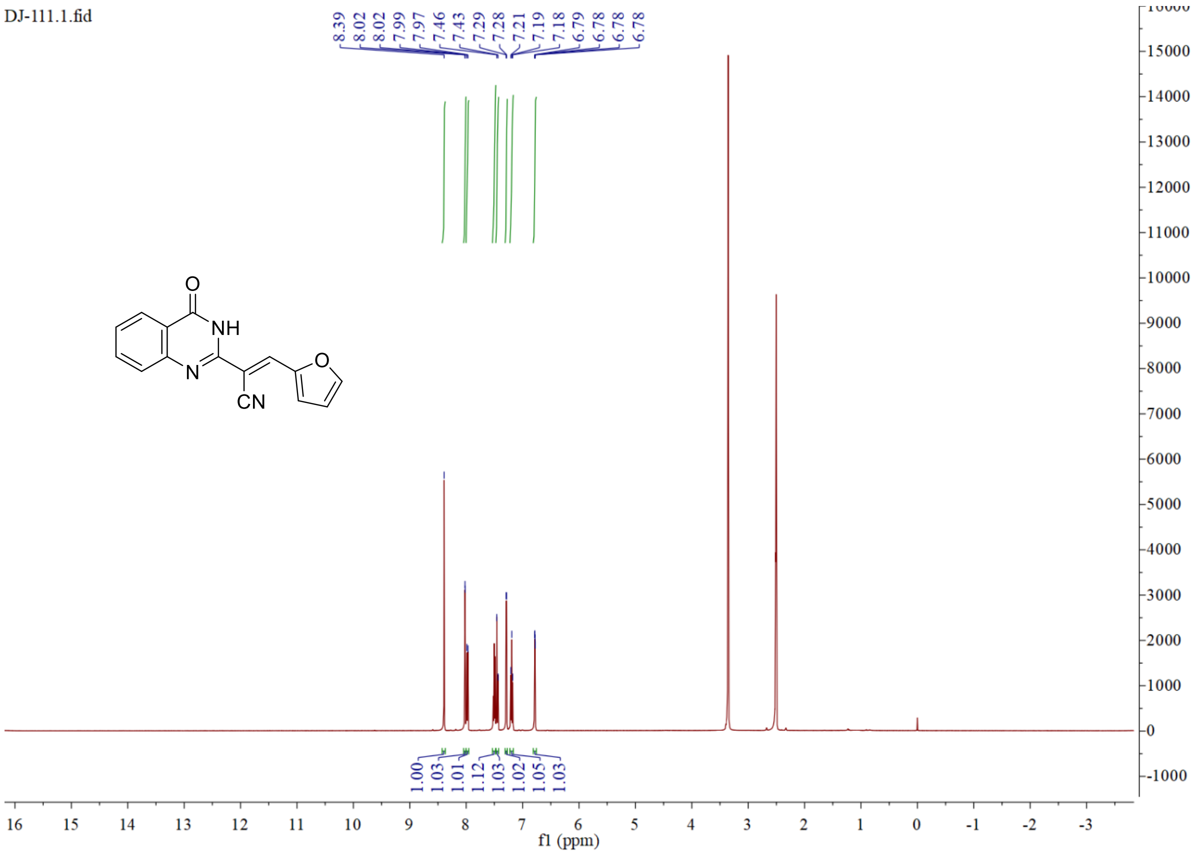

^{13}C NMR spectrum

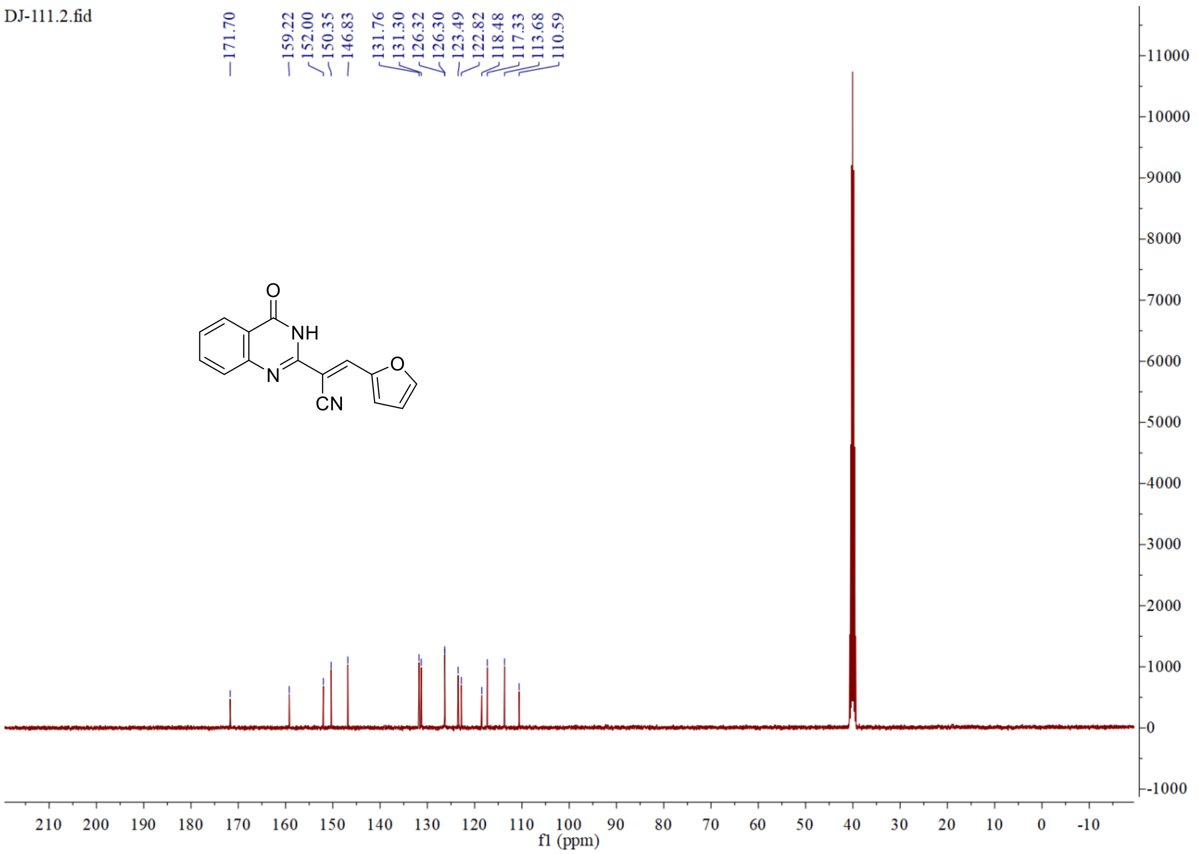

HRMS spectrum

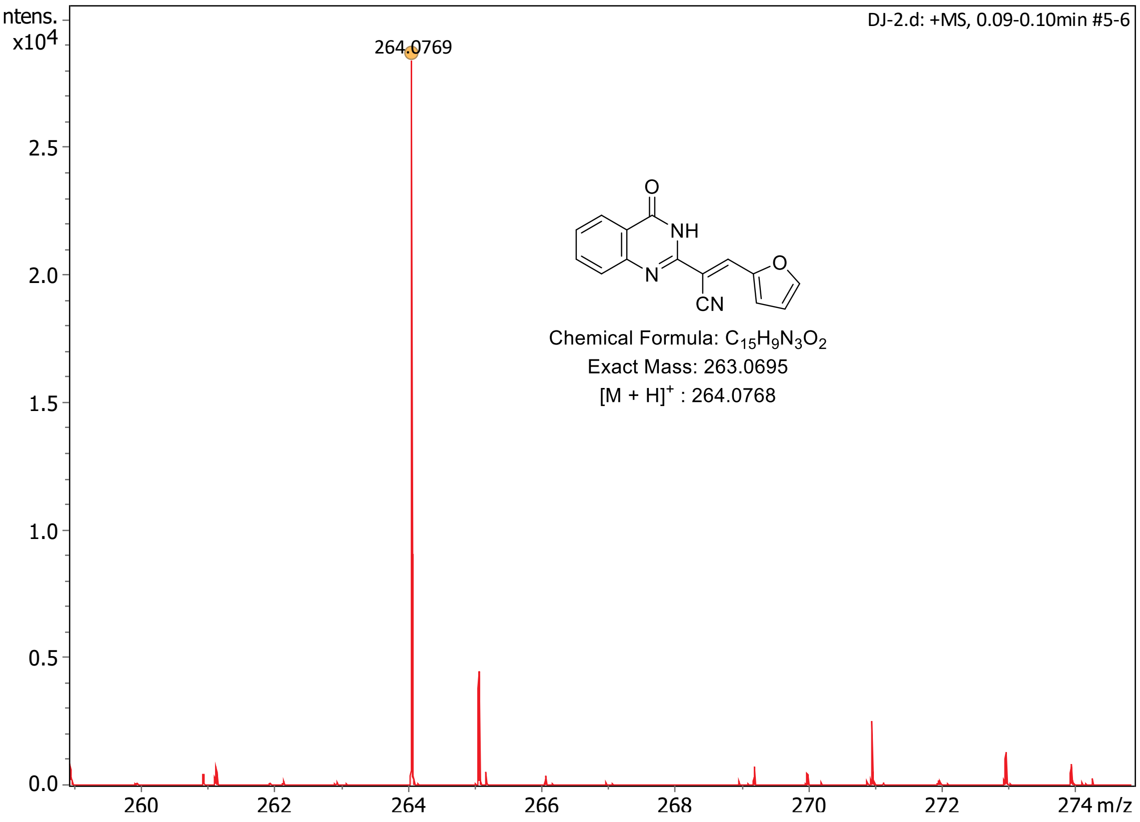

12.2 Spectra of compound **4b**

^{1}H NMR spectrum

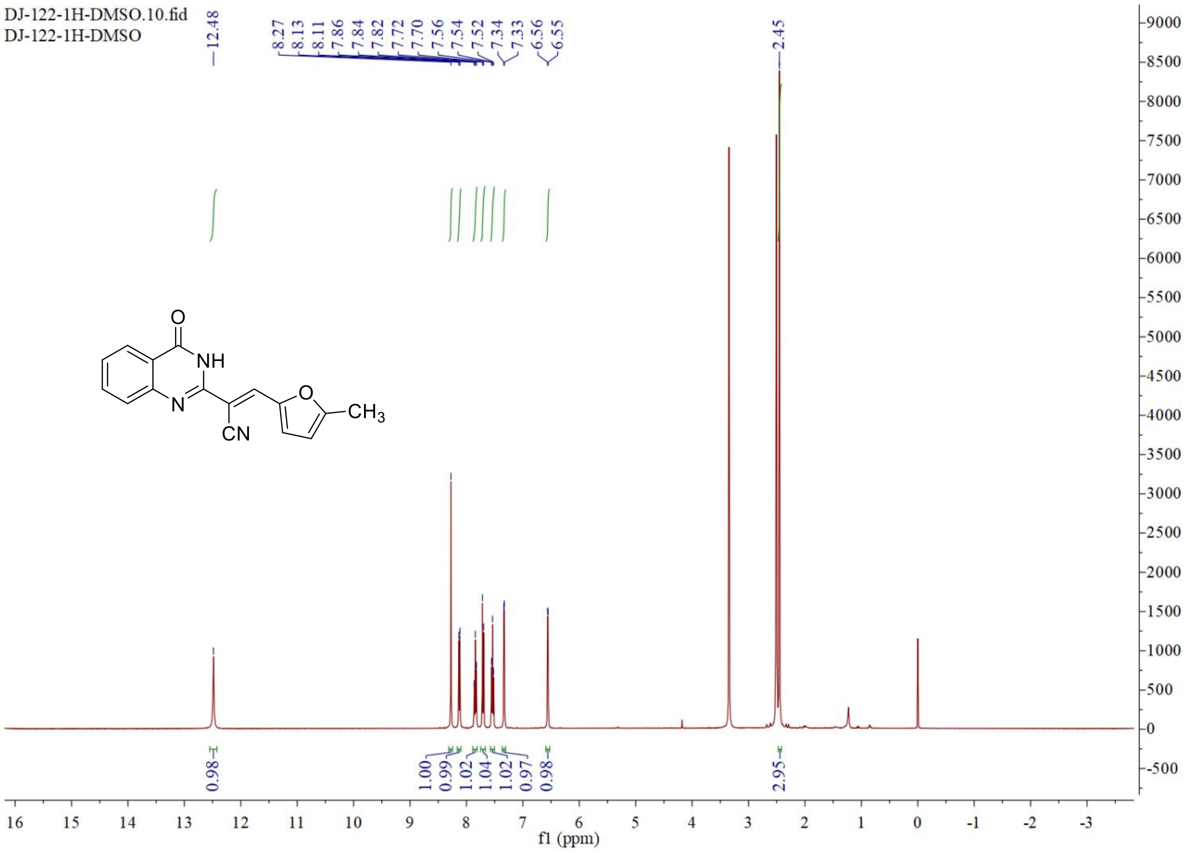

^{13}C NMR spectrum

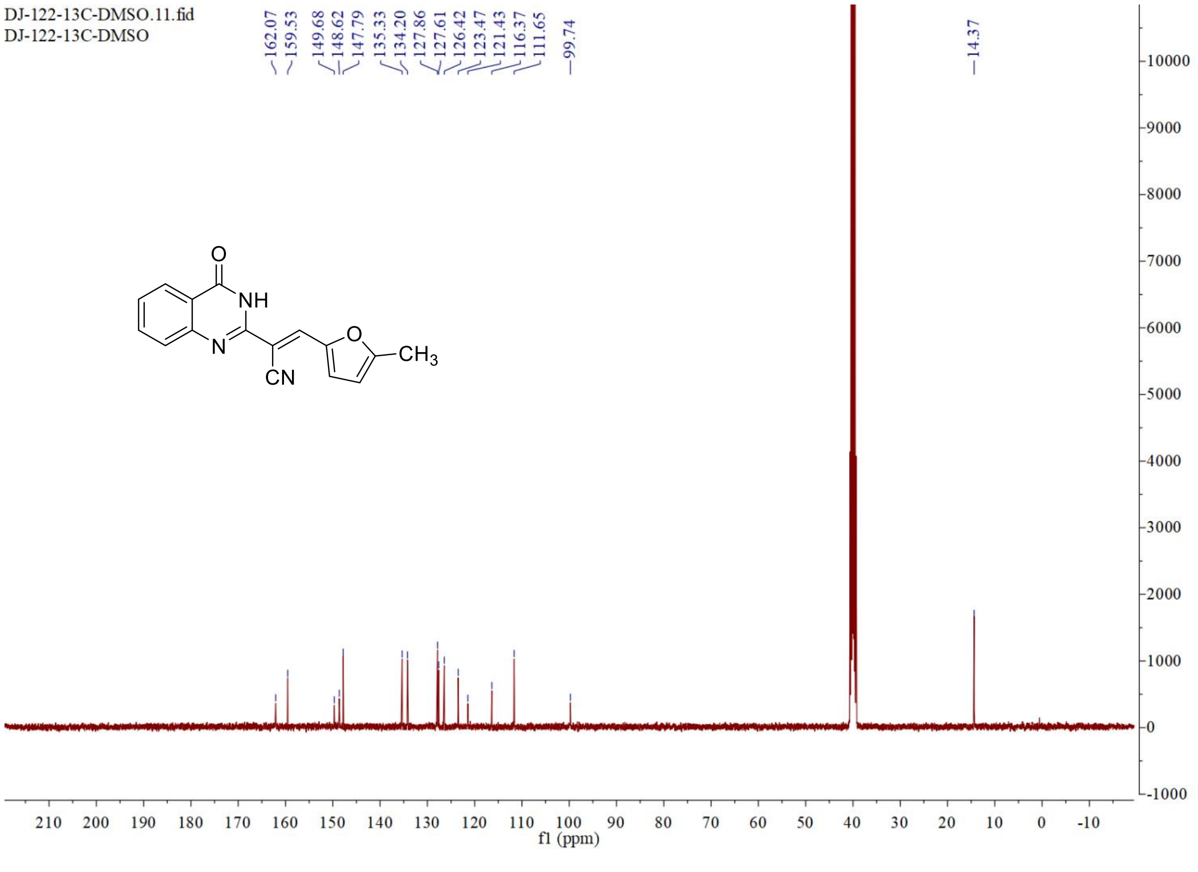

HRMS spectrum

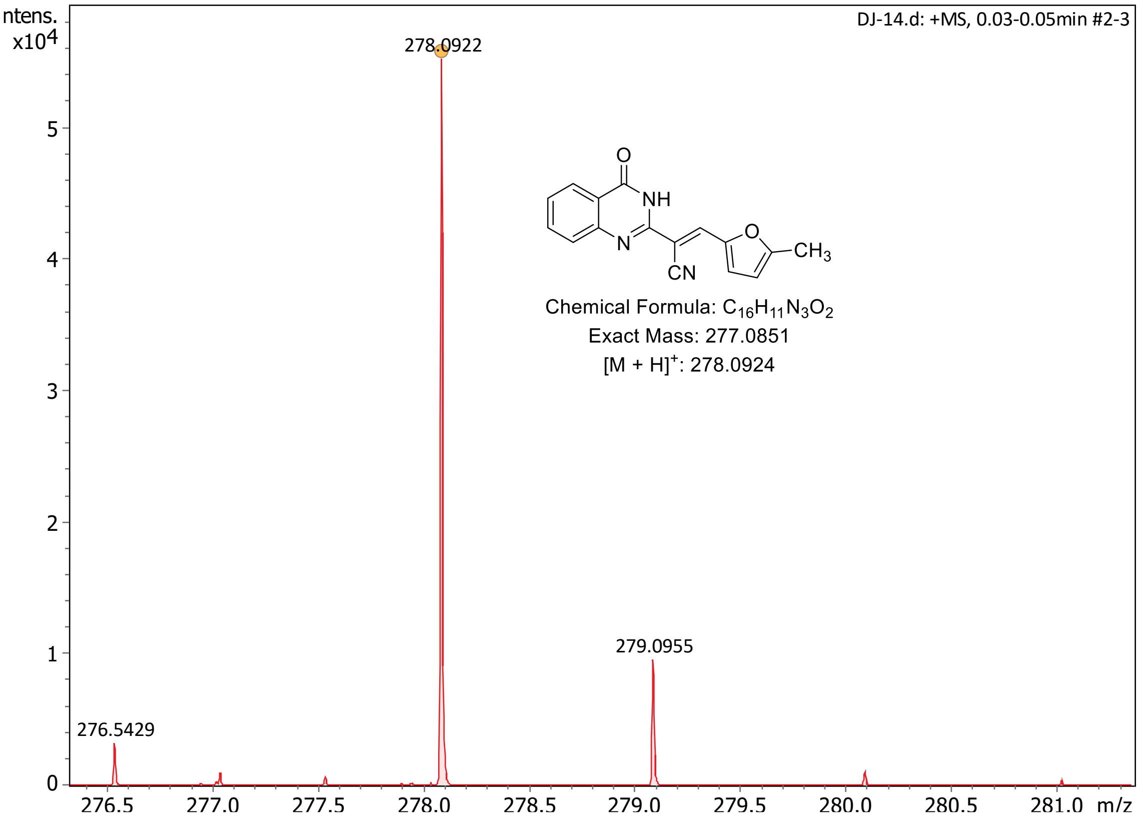

12.3 Spectra of compound **4c**

^{1}H NMR spectrum

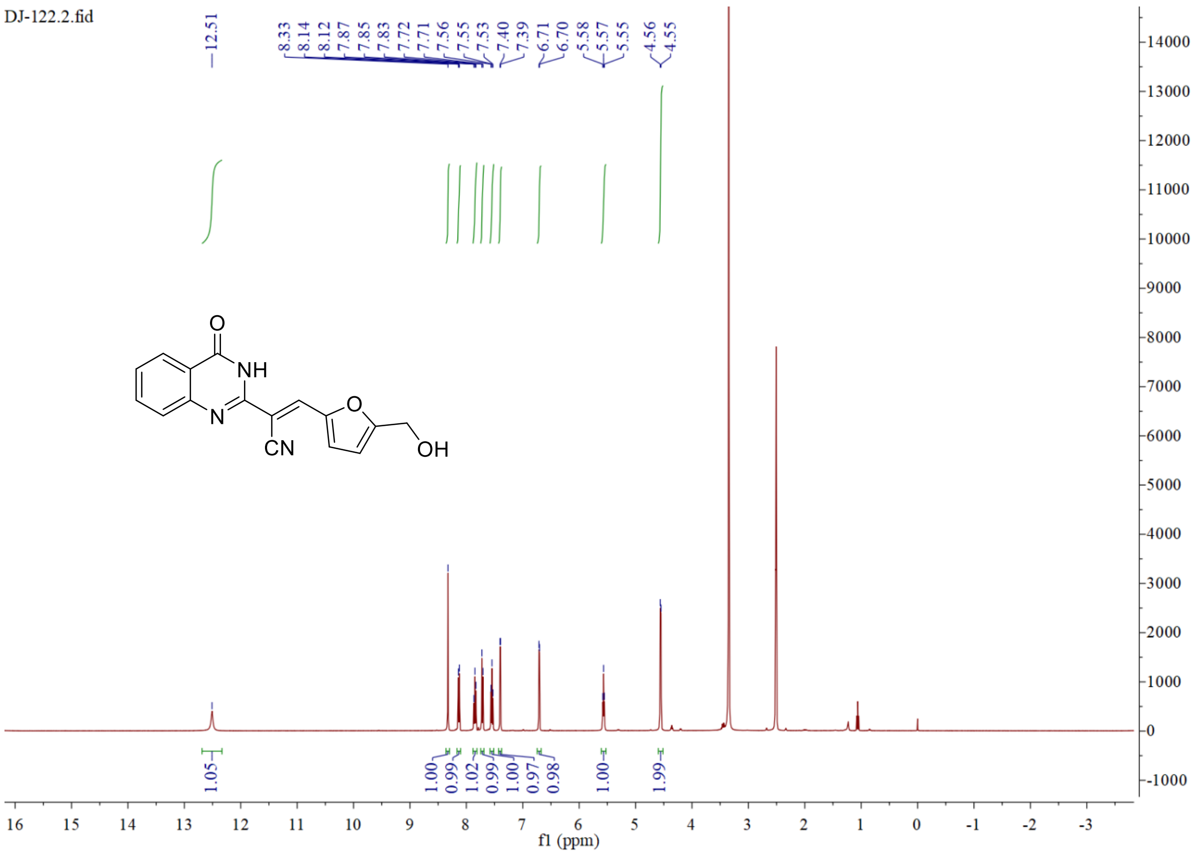

^{13}C NMR spectrum

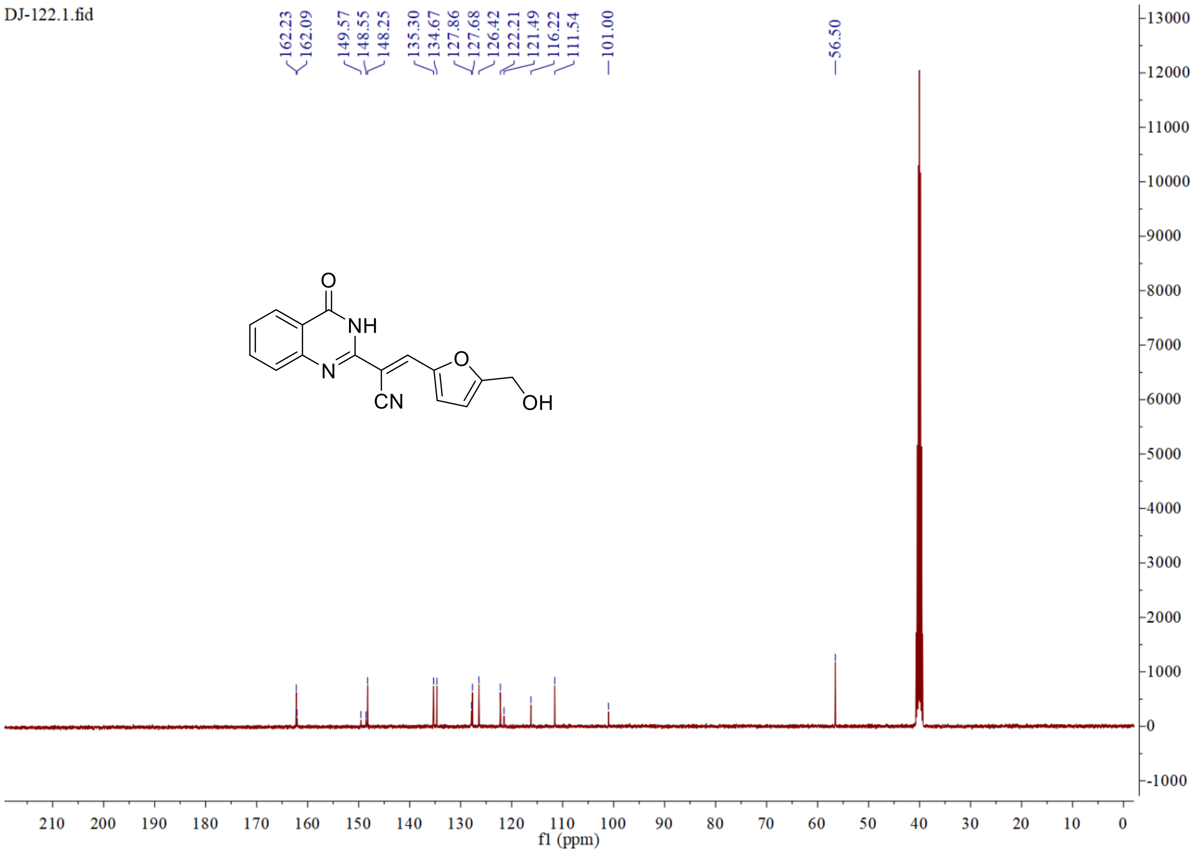

HRMS spectrum

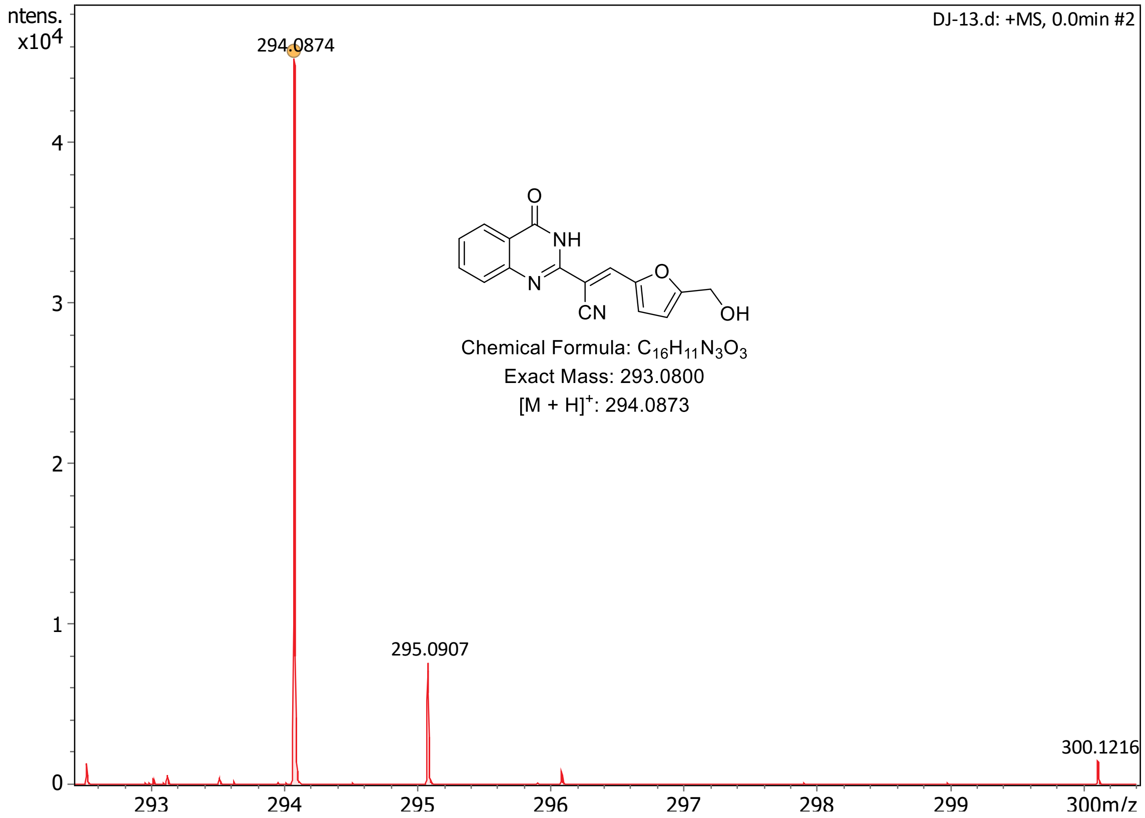

12.4 Spectra of compound **5a**

^{1}H NMR spectrum

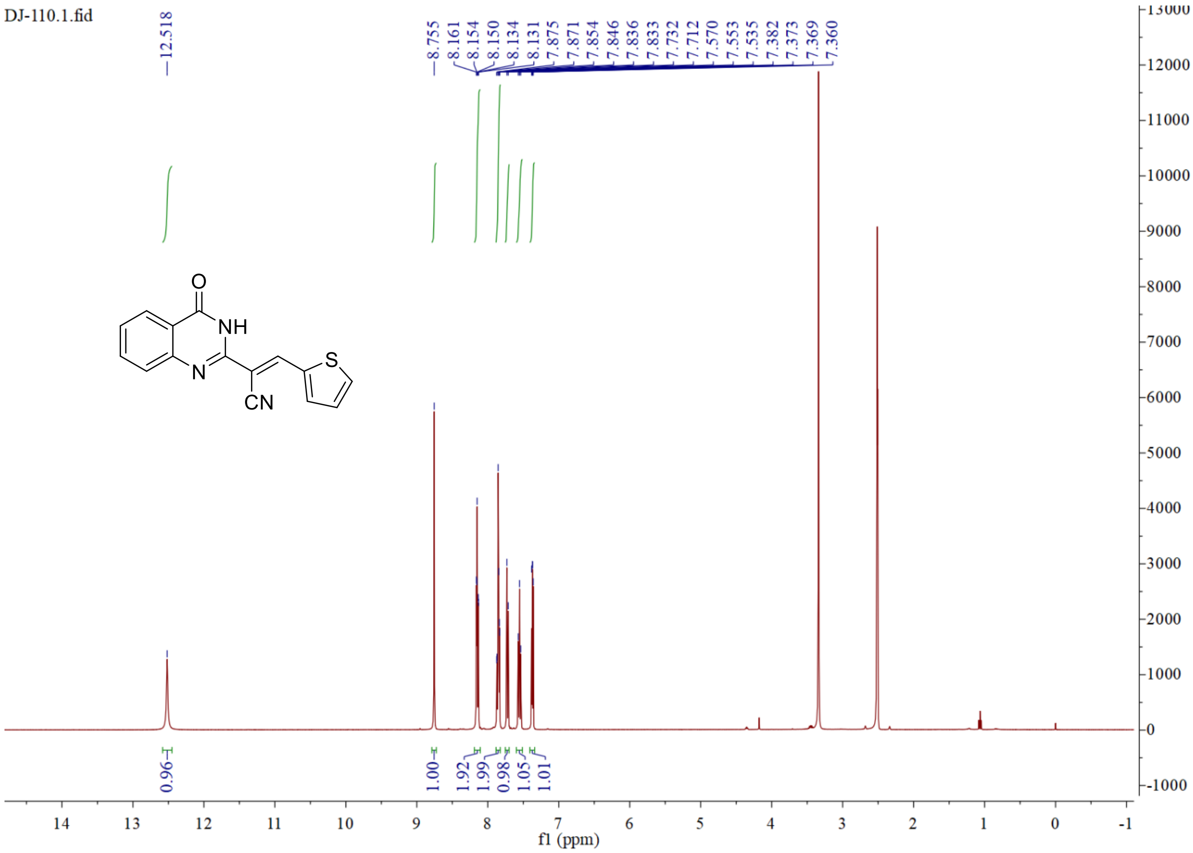

^{13}C NMR spectrum

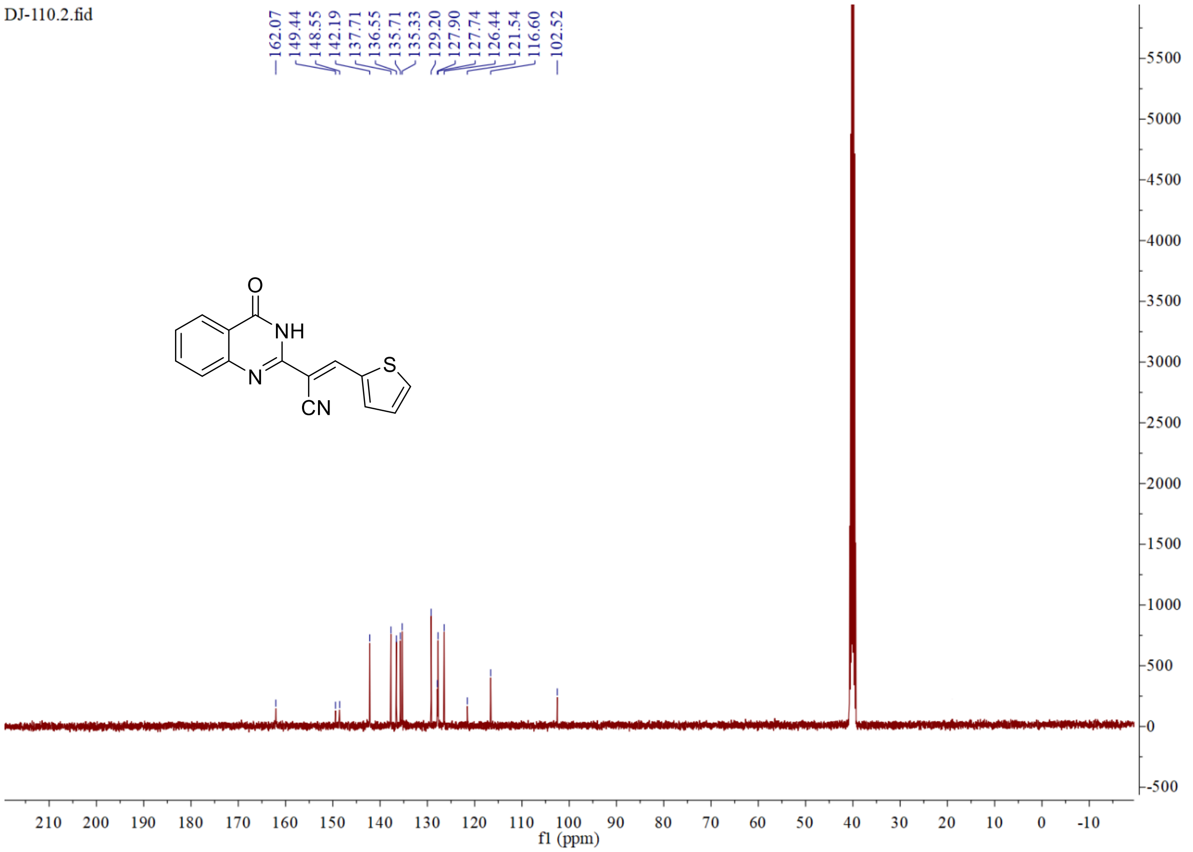

HRMS spectrum

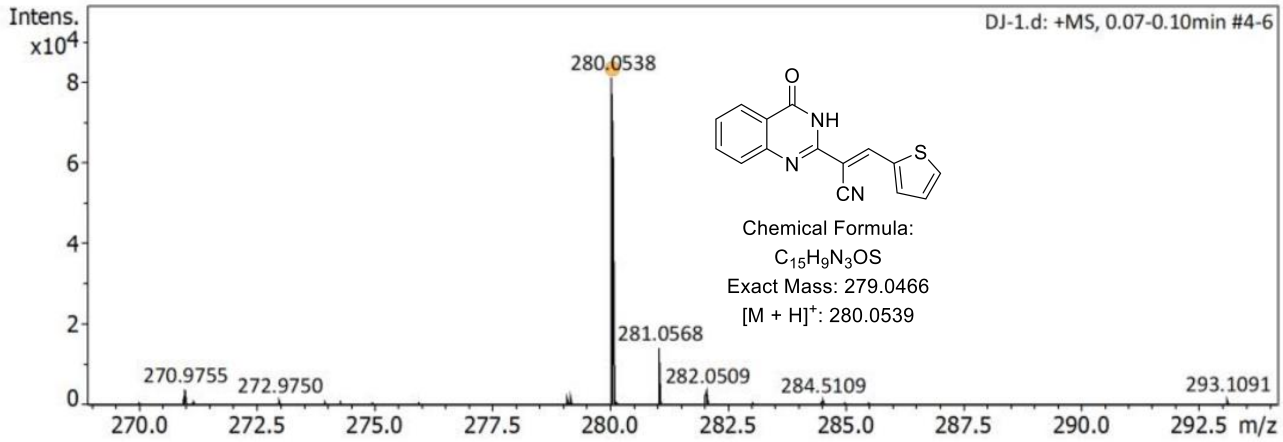

eas. m/z	#	Ion Formula	m/z	err [ppm]	mSigma	# mSigma	Score	rdb	e¡¥ Conf	N-Rule
280.0538	1	C15H10N3OS	280.0539	0.5	10.0	1	100.00	15.0	even	ok

12.5 Spectra of compound **6b**

^{1}H NMR spectrum

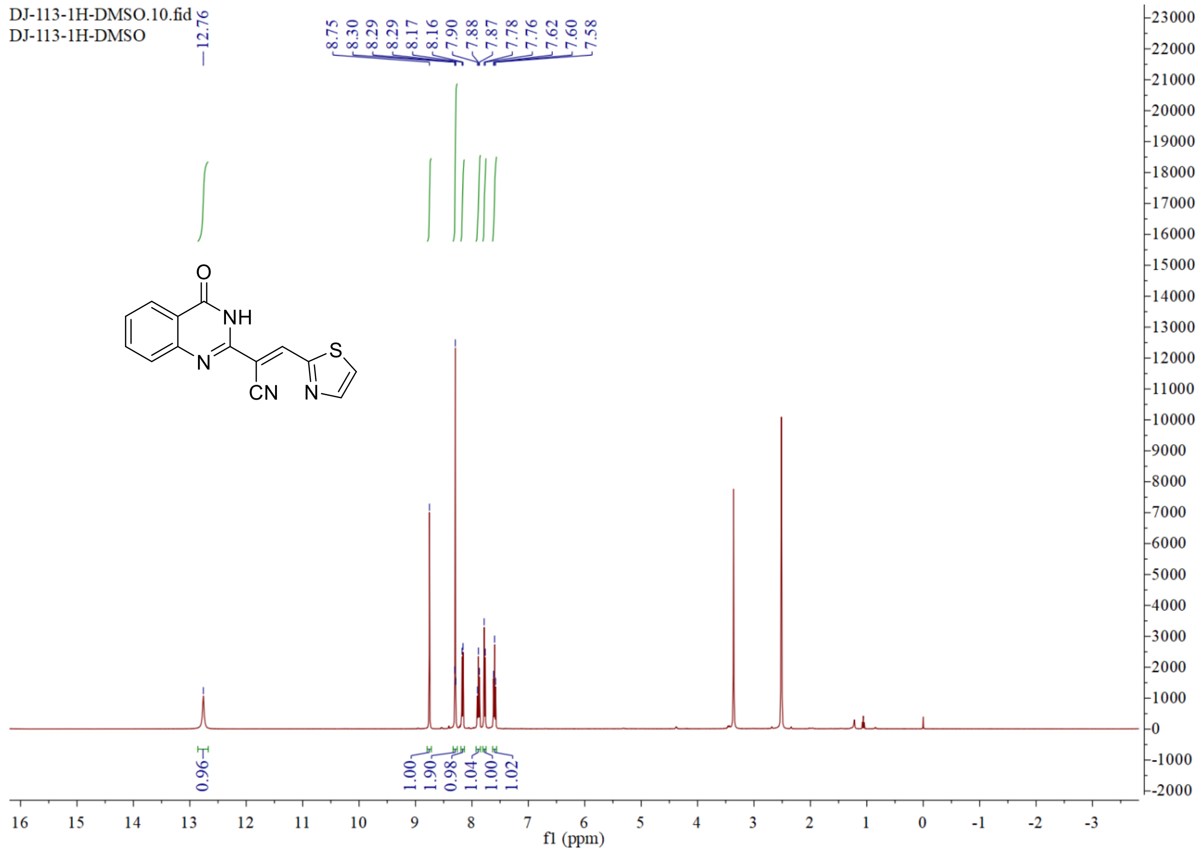

^{13}C NMR spectrum

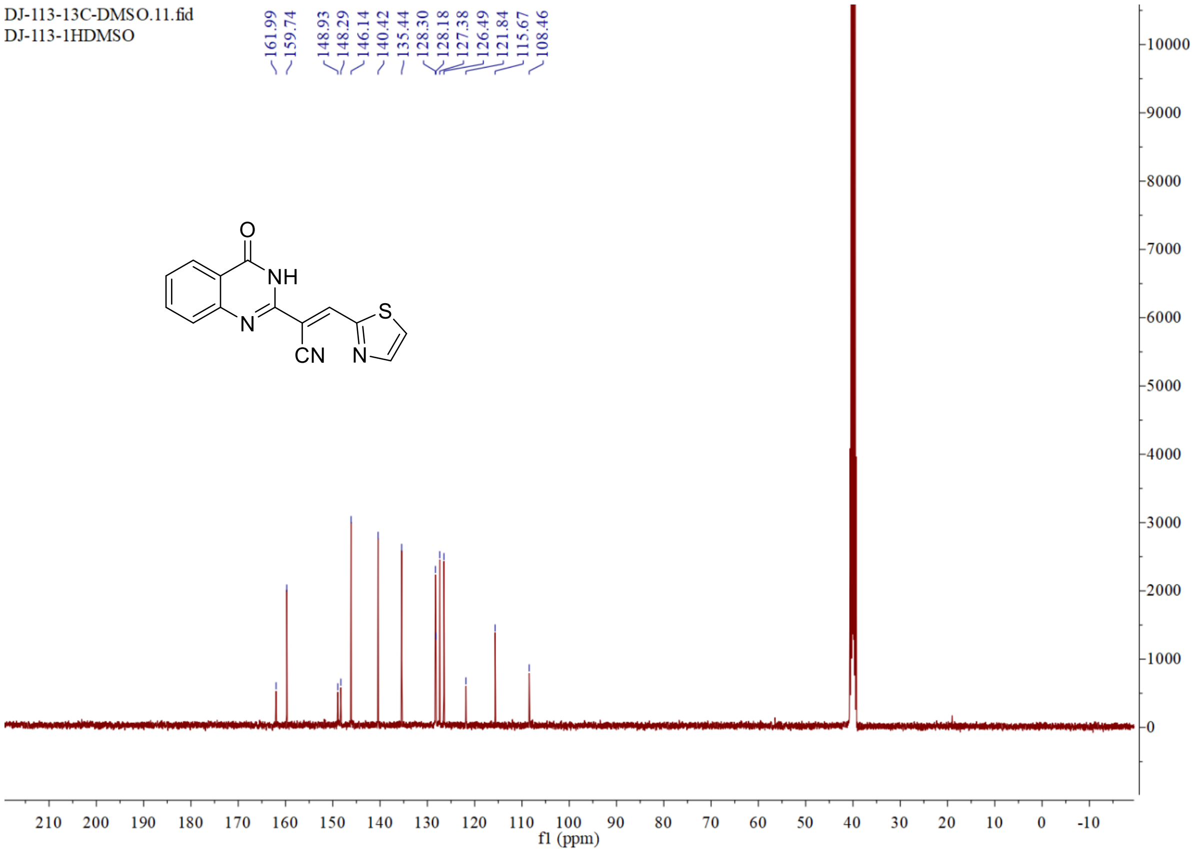

HRMS spectrum

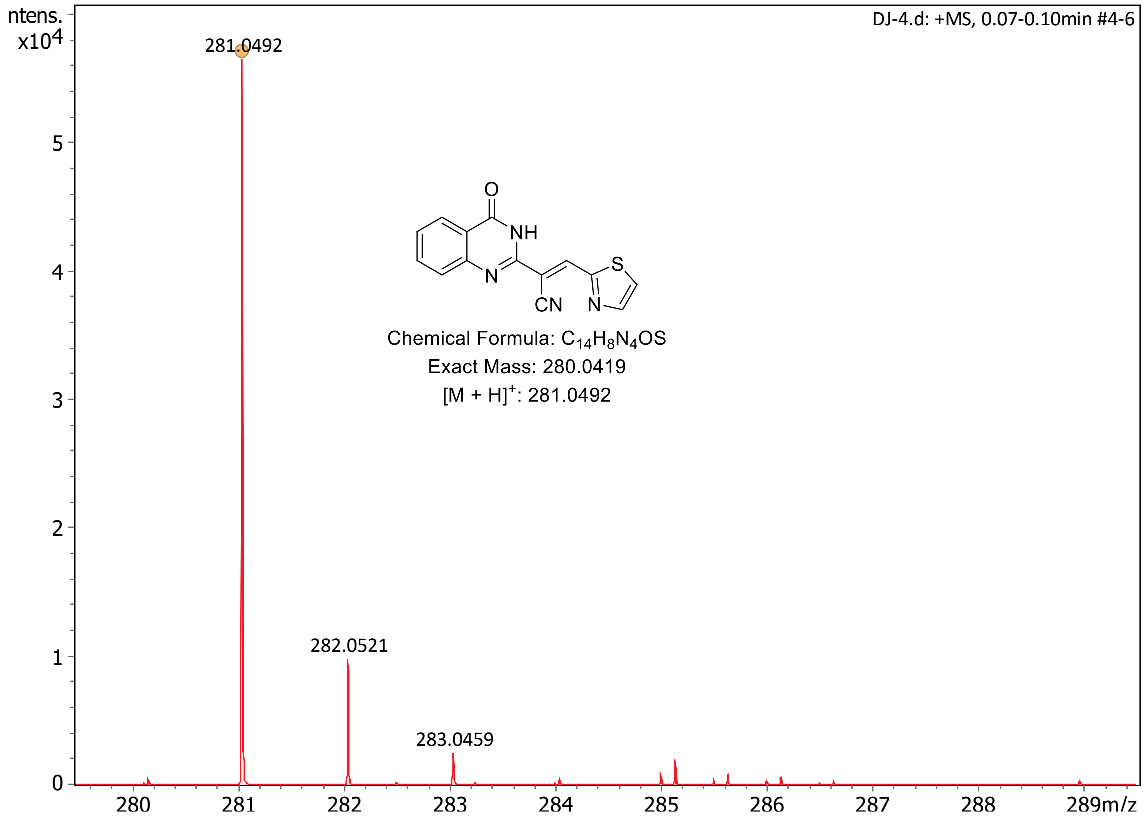

12.6 Spectra of compound **6c**

^{1}H NMR spectrum

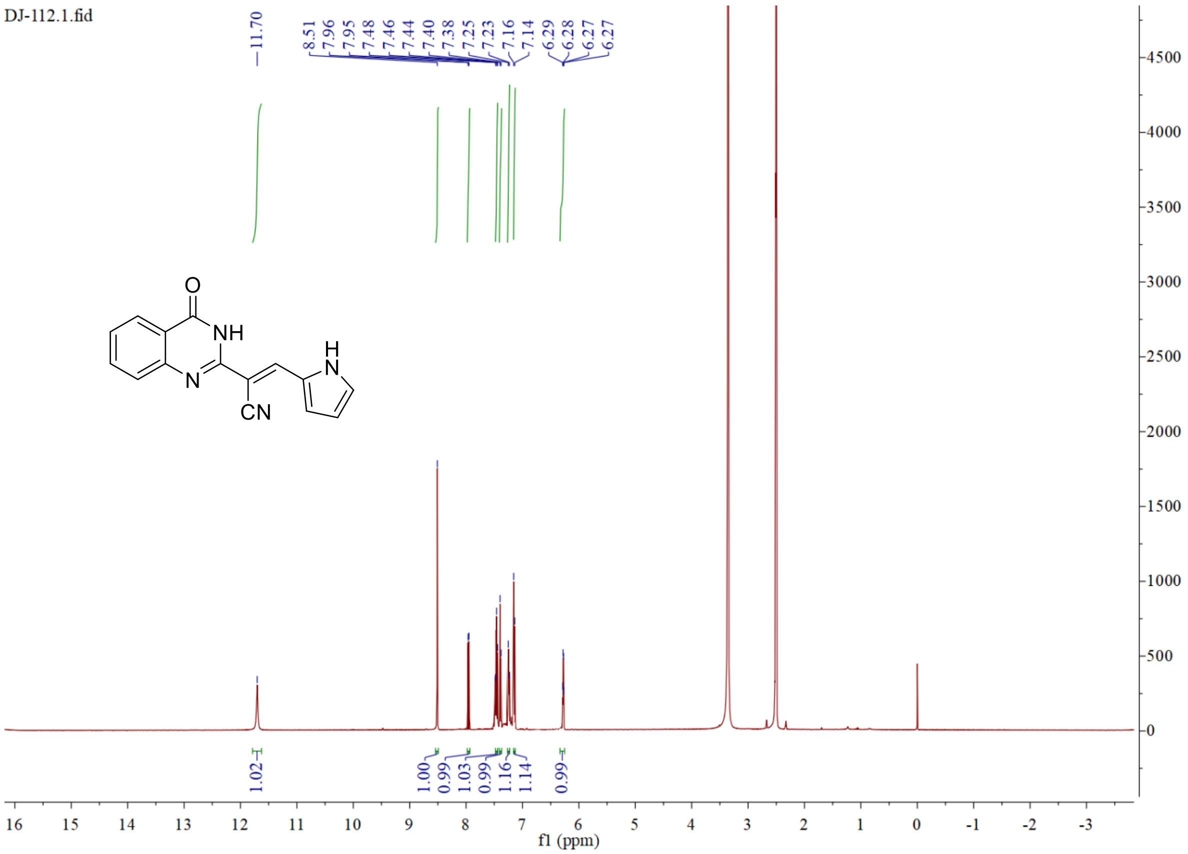

^{13}C NMR spectrum

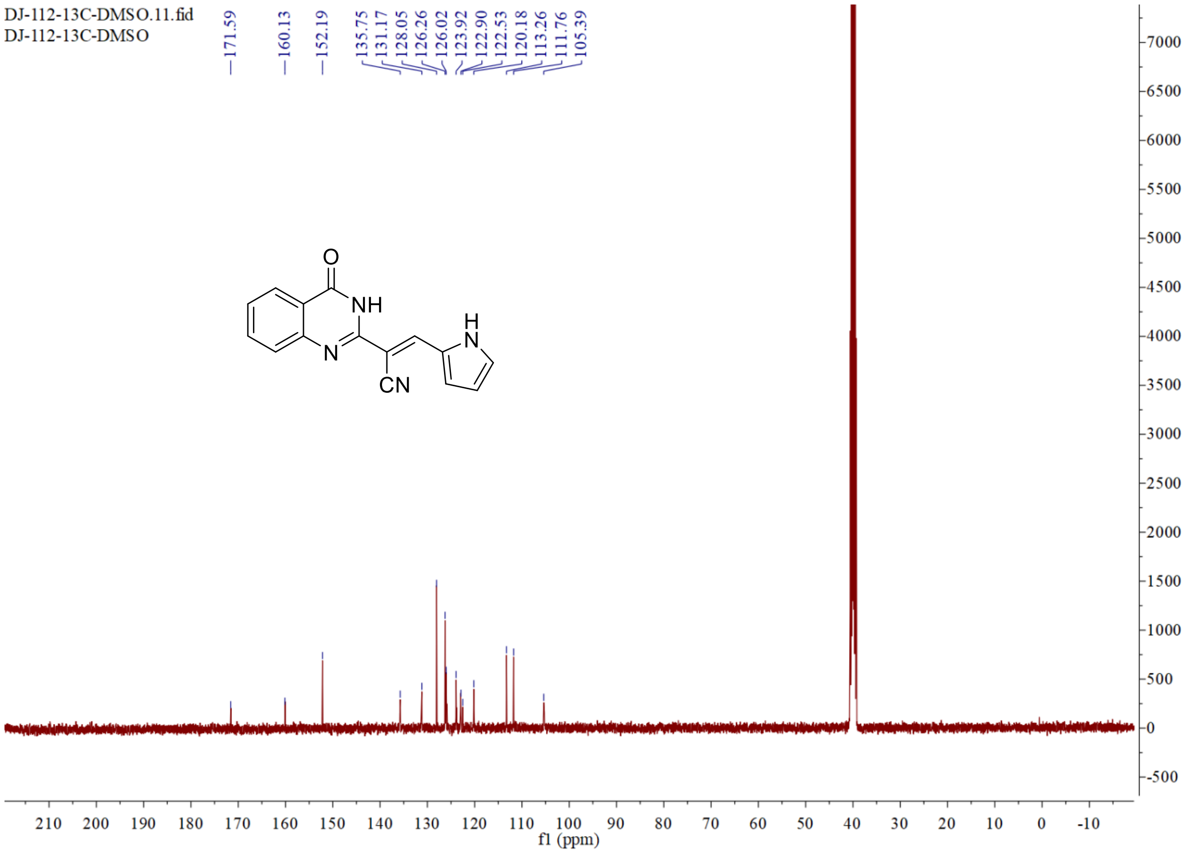

HRMS spectrum

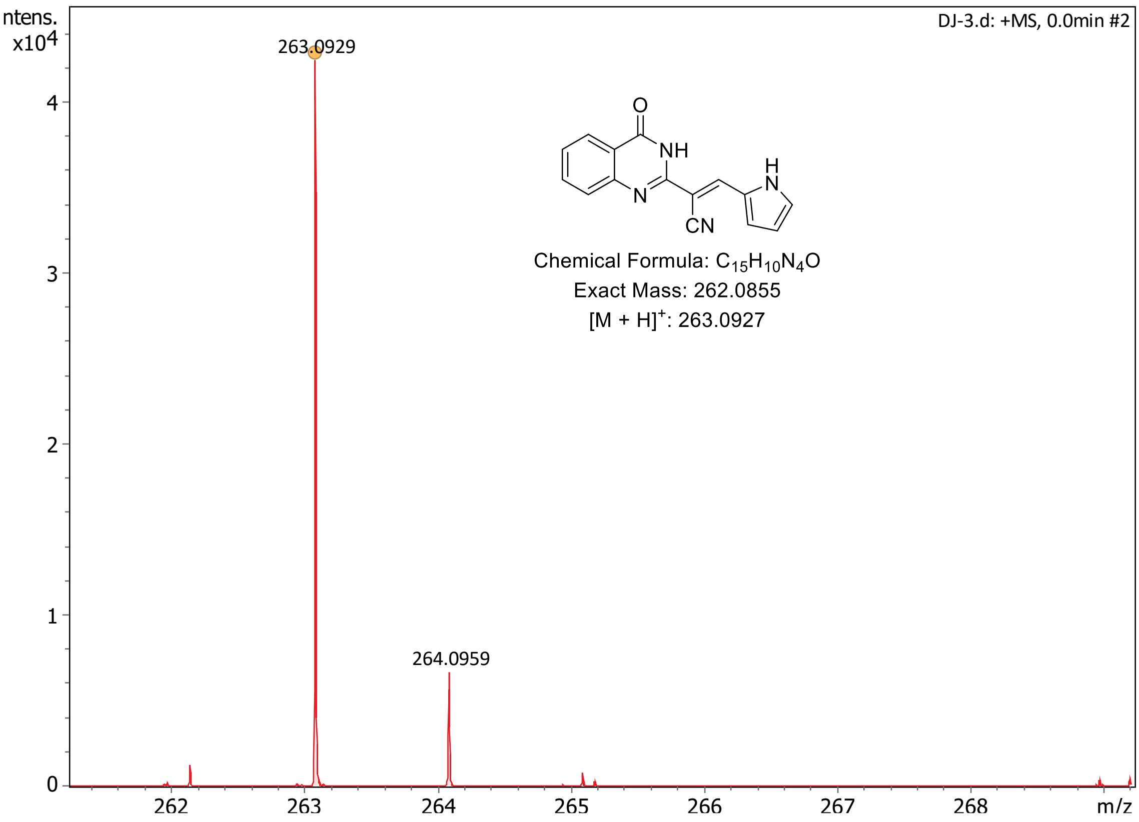

12.7 Spectra of compound **7**

^{1}H NMR spectrum

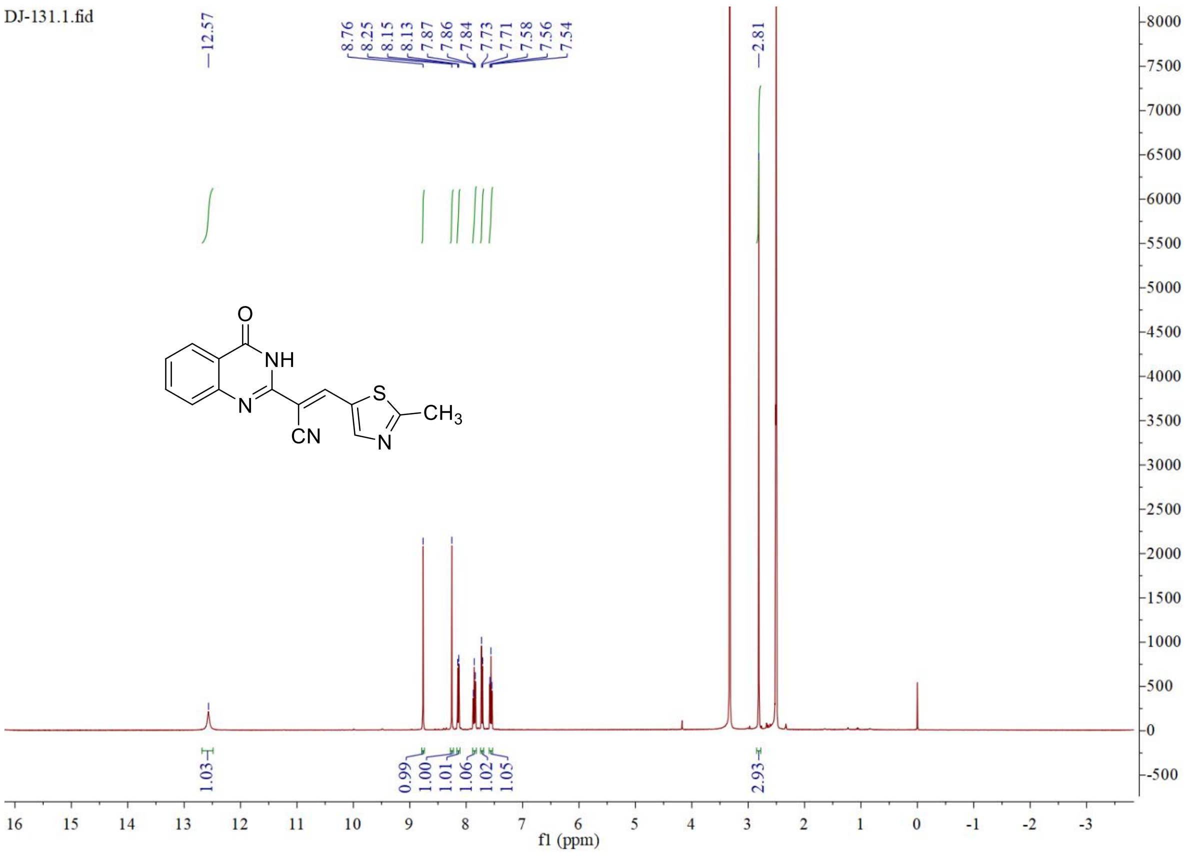

HRMS spectrum

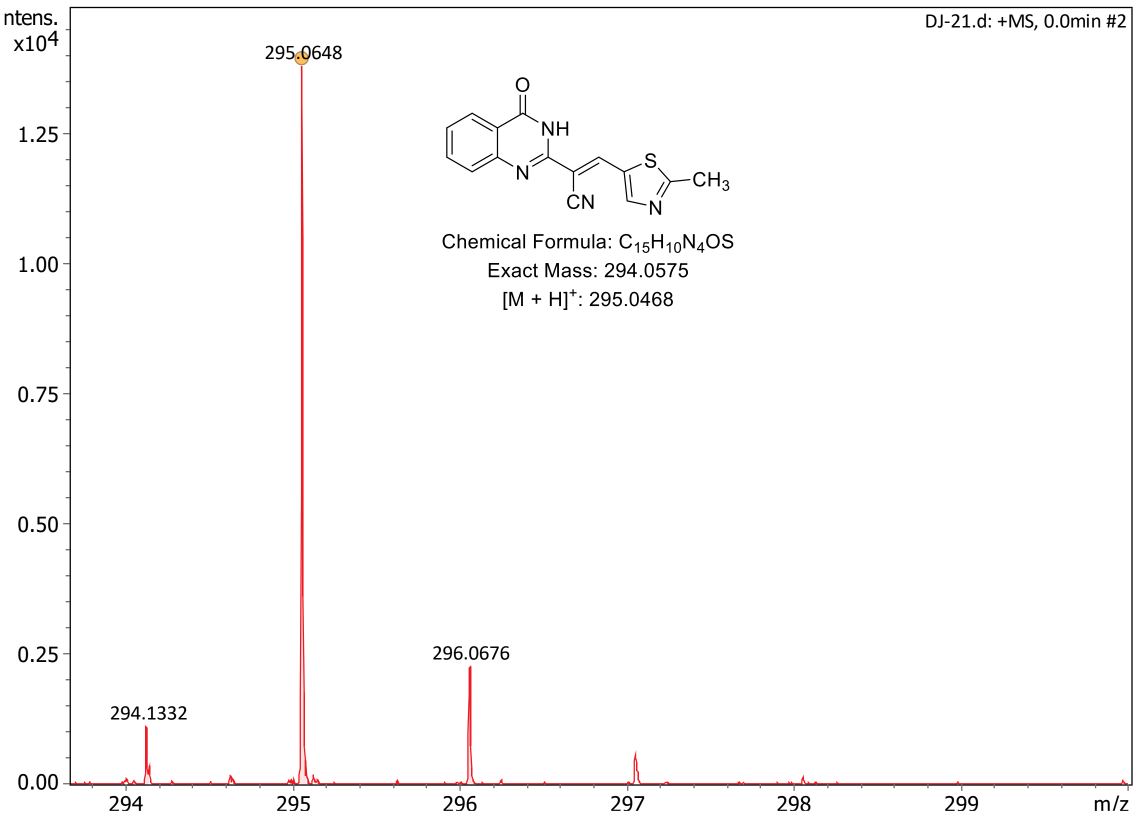

12.8 Spectra of compound **8a**

^{1}H NMR spectrum

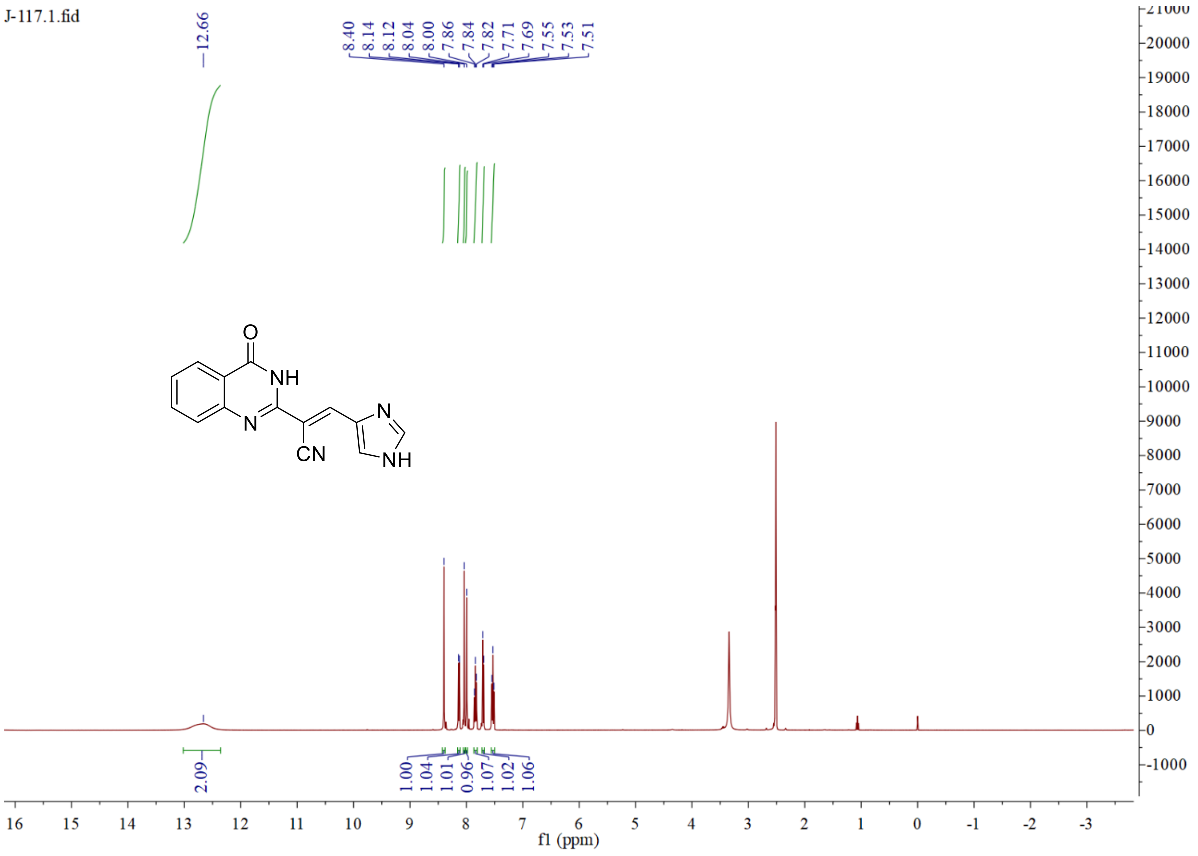

^{13}C NMR spectrum

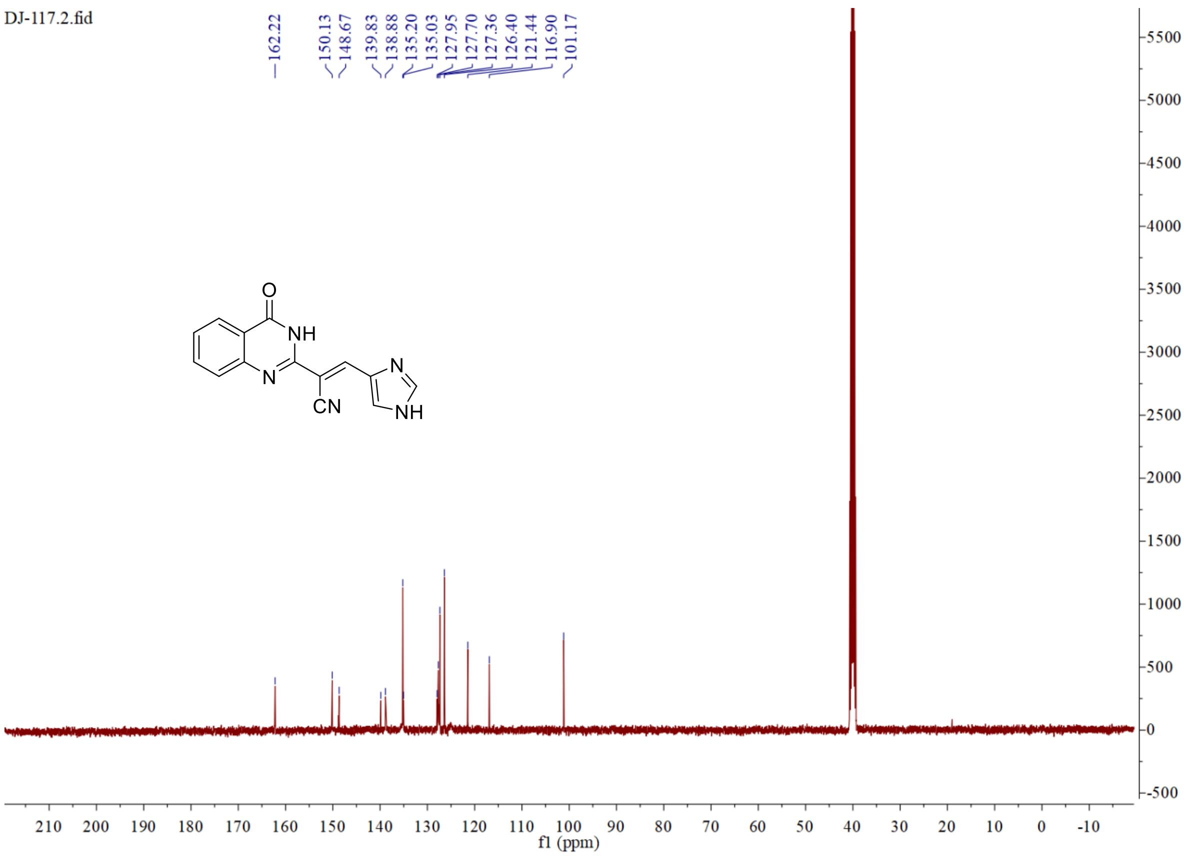

HRMS spectrum

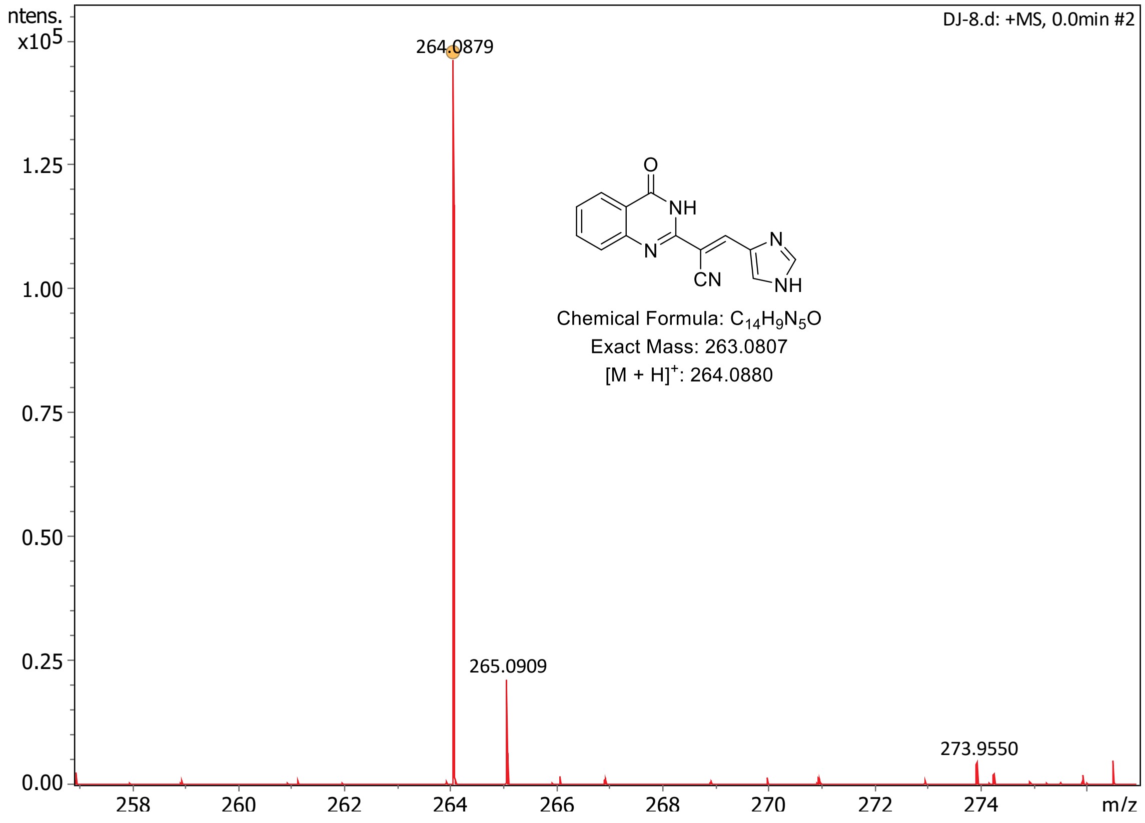

12.9 Spectra of compound **8b**

^{1}H NMR spectrum

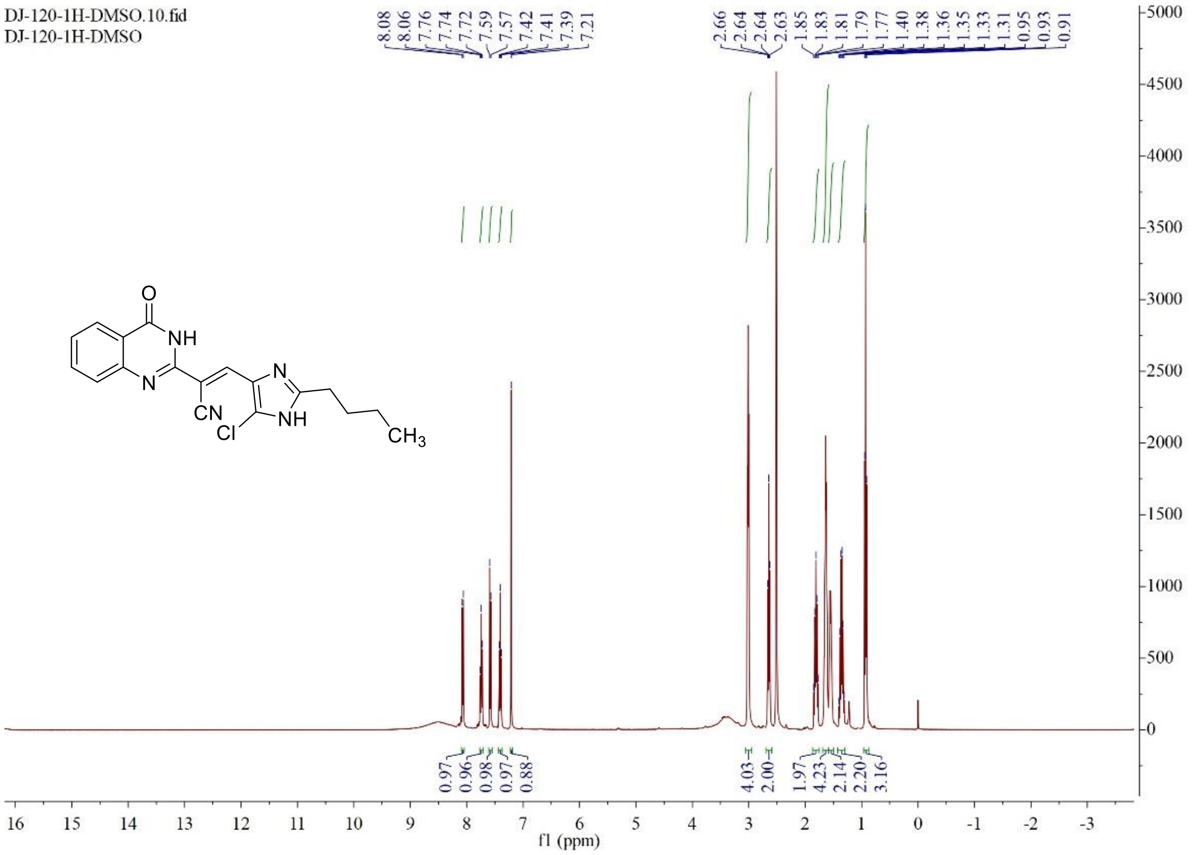

^{13}C NMR spectrum

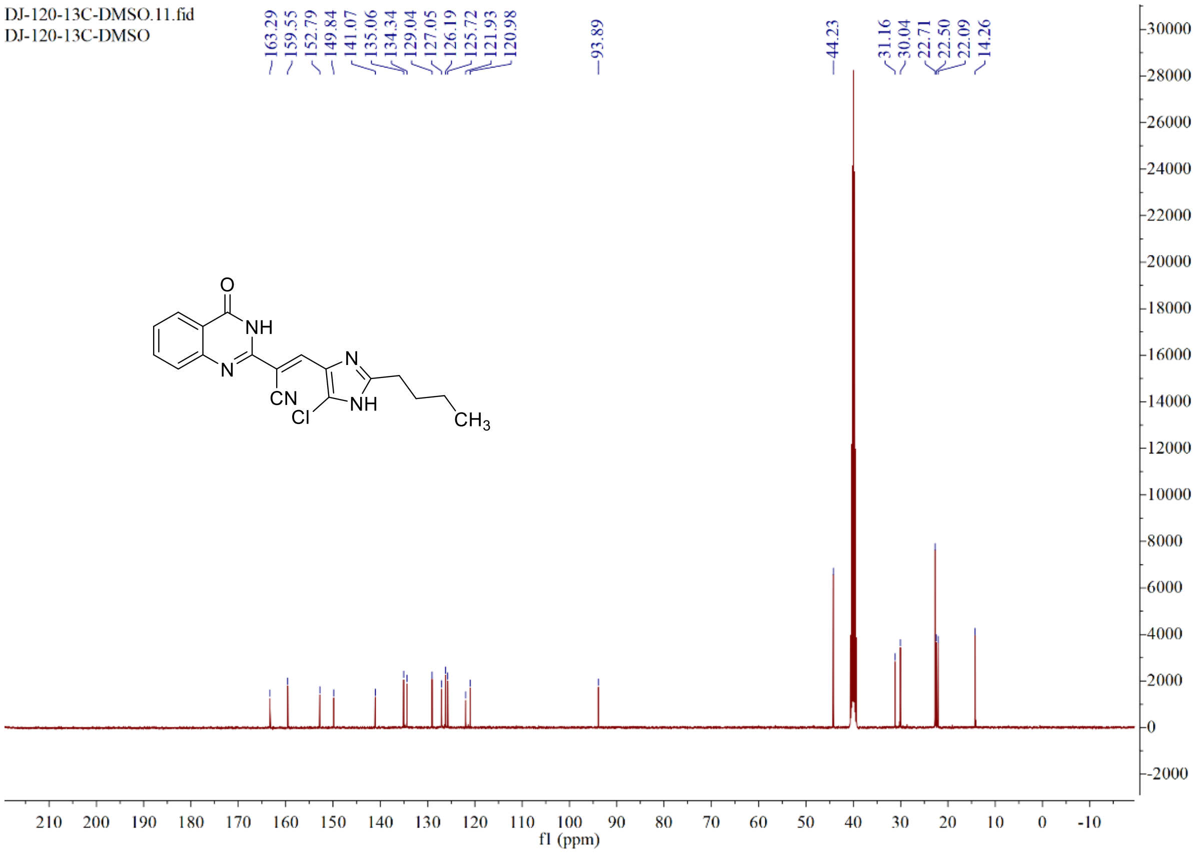

HRMS spectrum

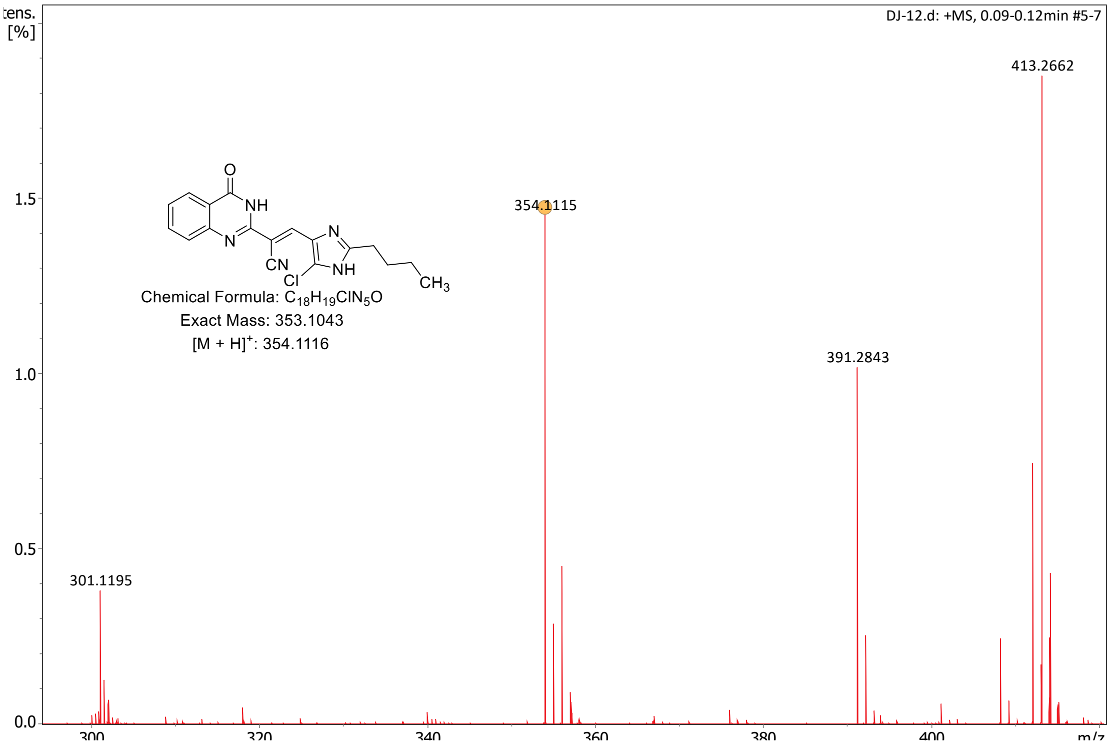

12.10 Spectra of compound **9a**

^{1}H NMR spectrum

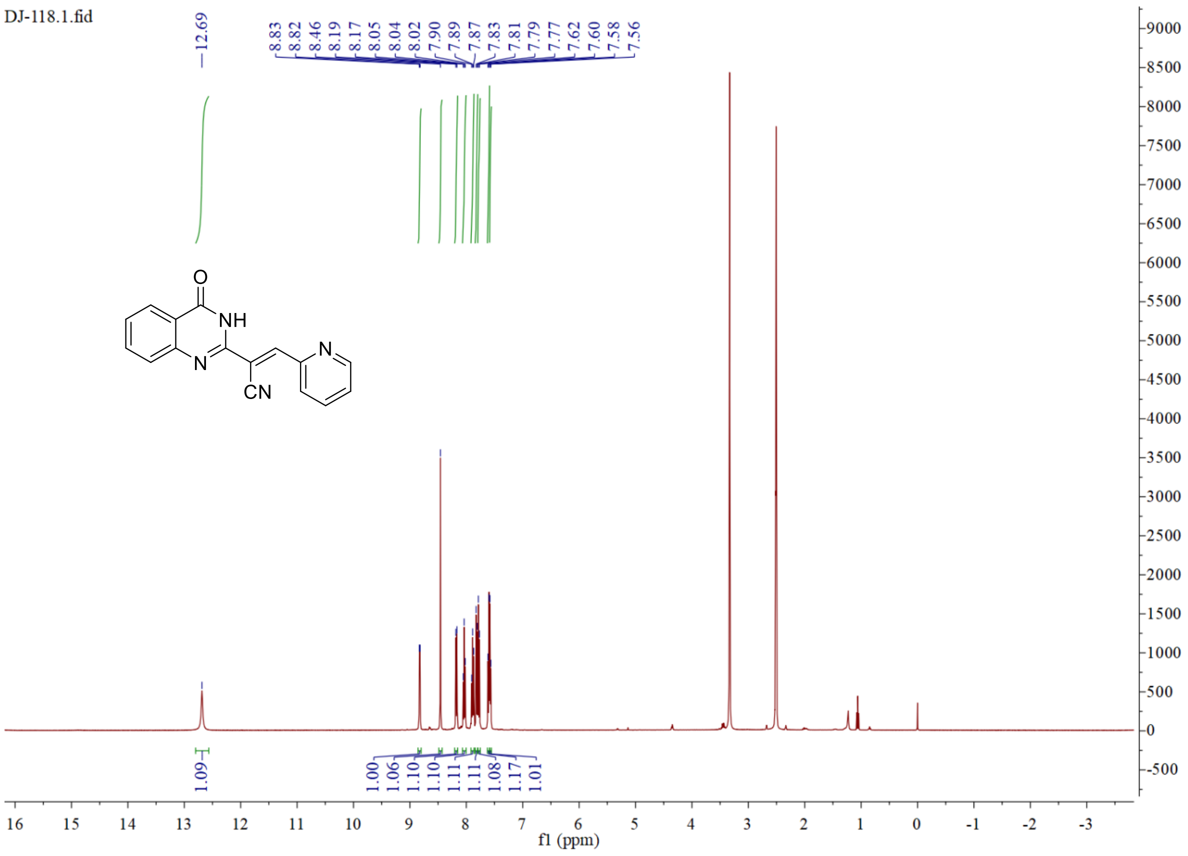

^{13}C NMR spectrum

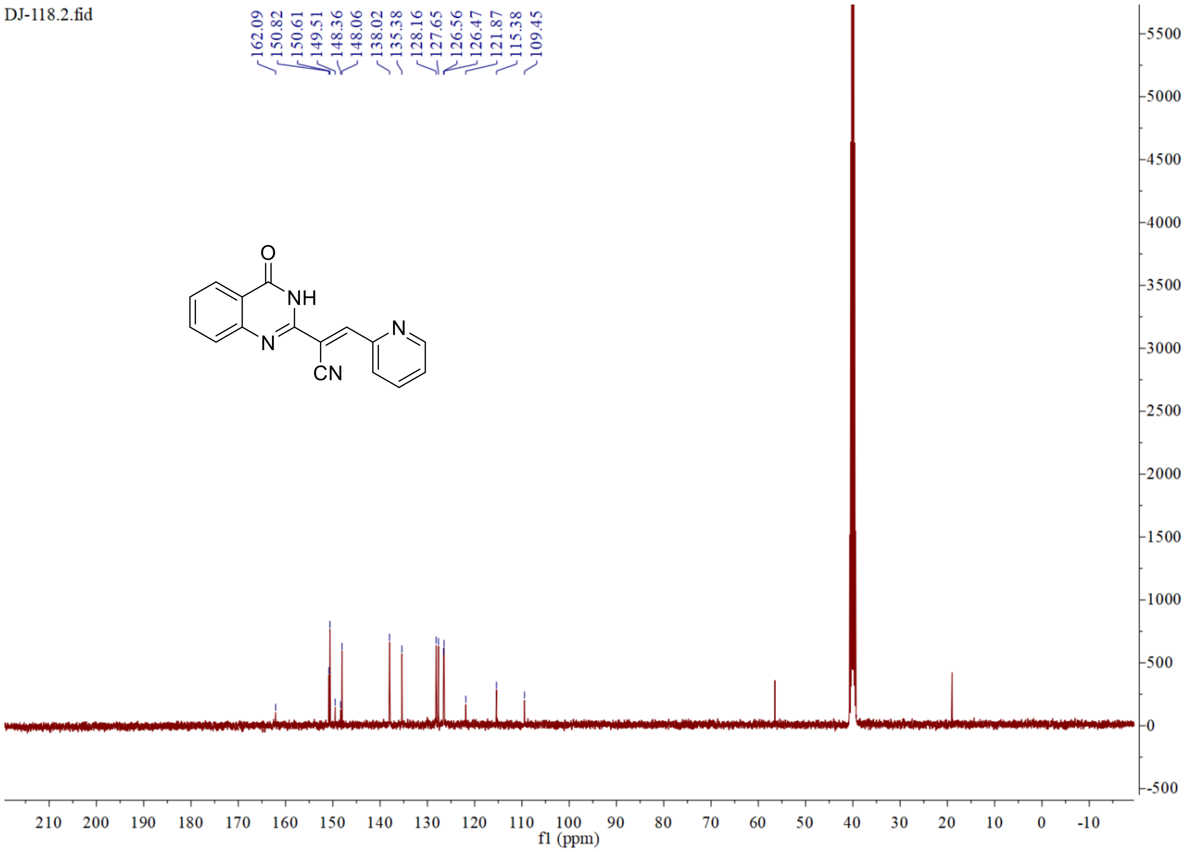

HRMS spectrum

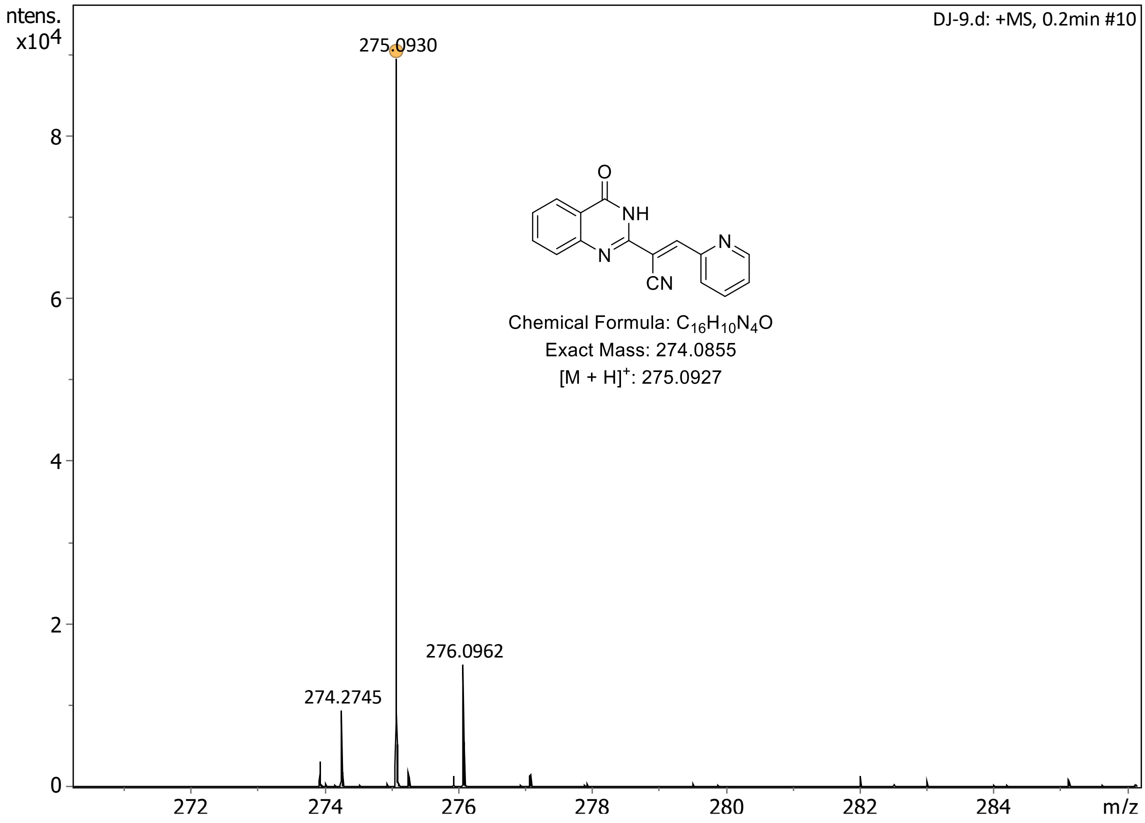

12.11 Spectra of compound **9b**

^{1}H NMR spectrum

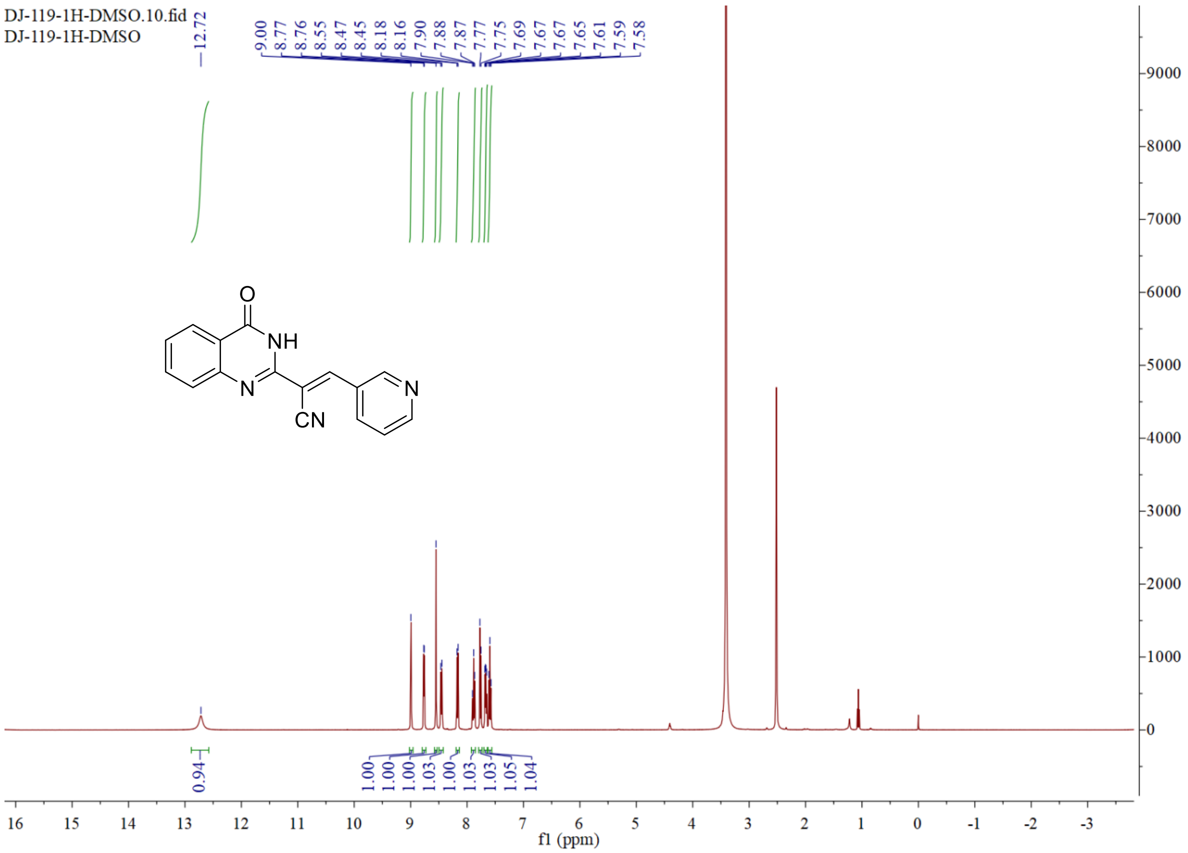

^{13}C NMR spectrum

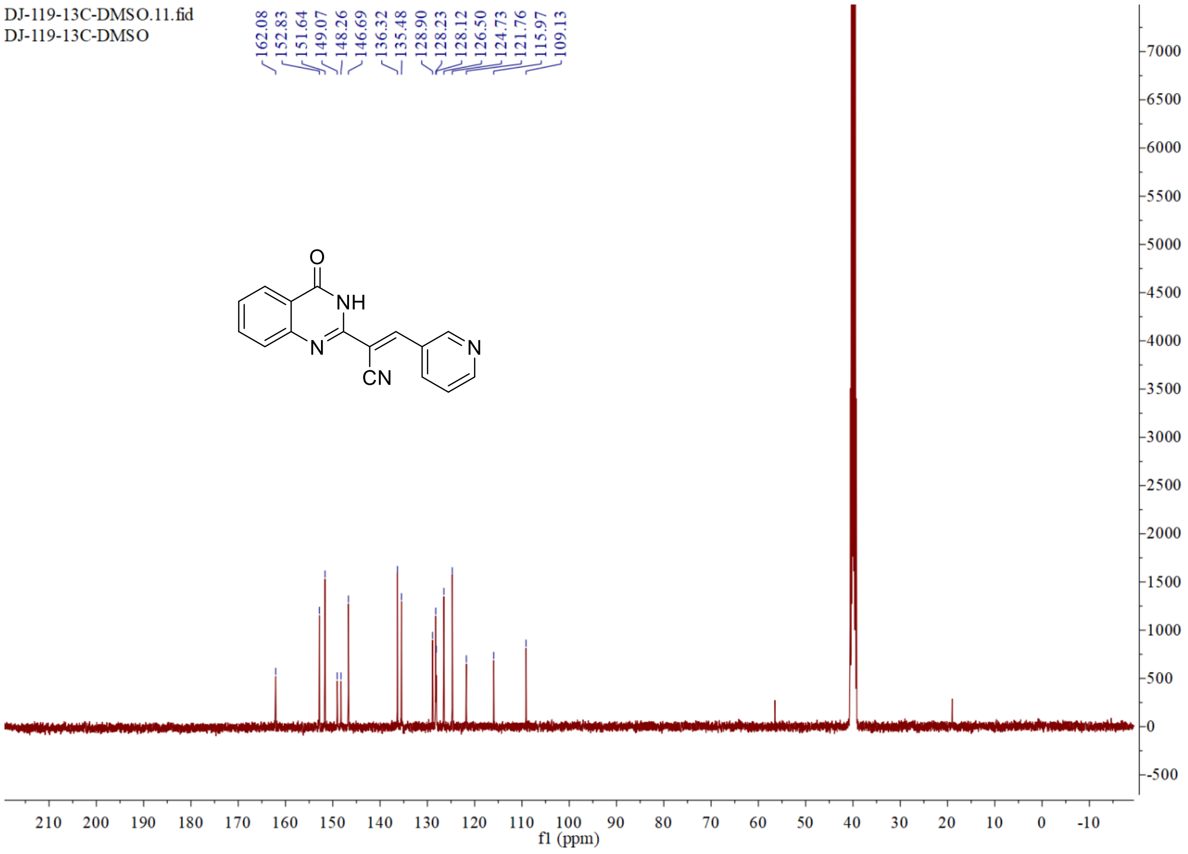

HRMS spectrum

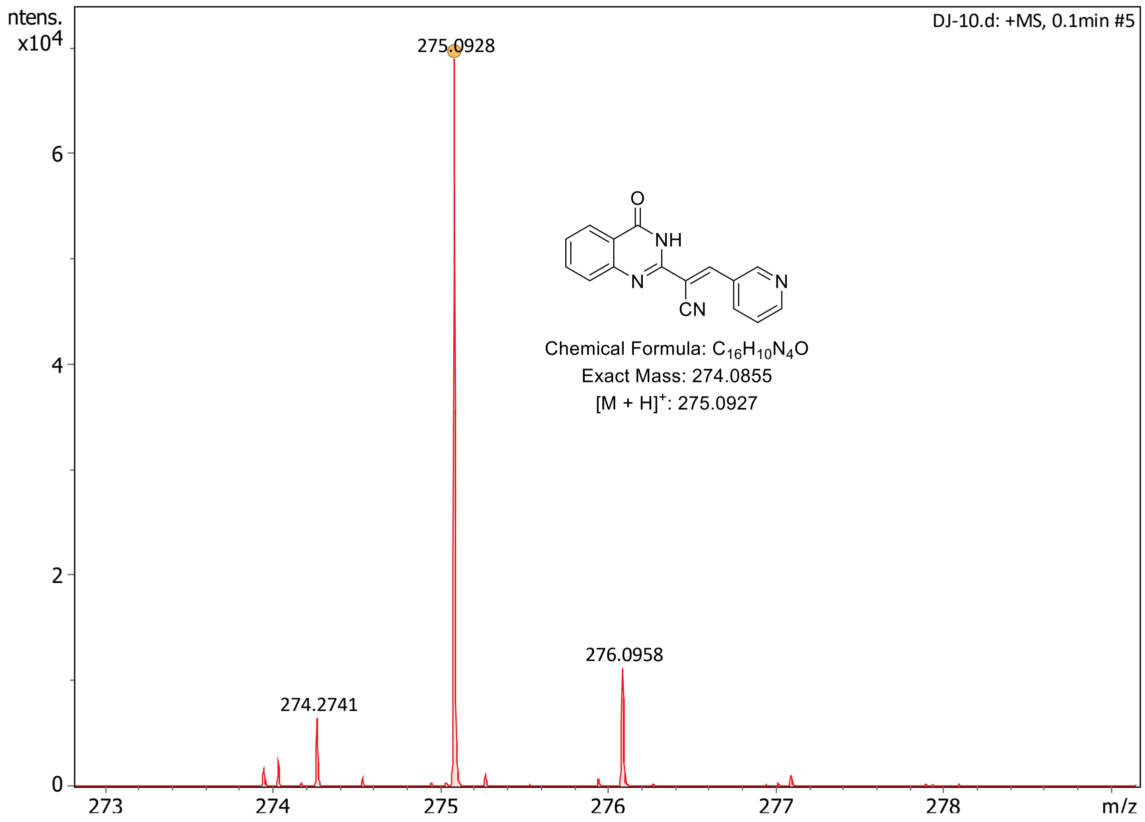

12.12 Spectra of compound **9c**

^{1}H NMR spectrum

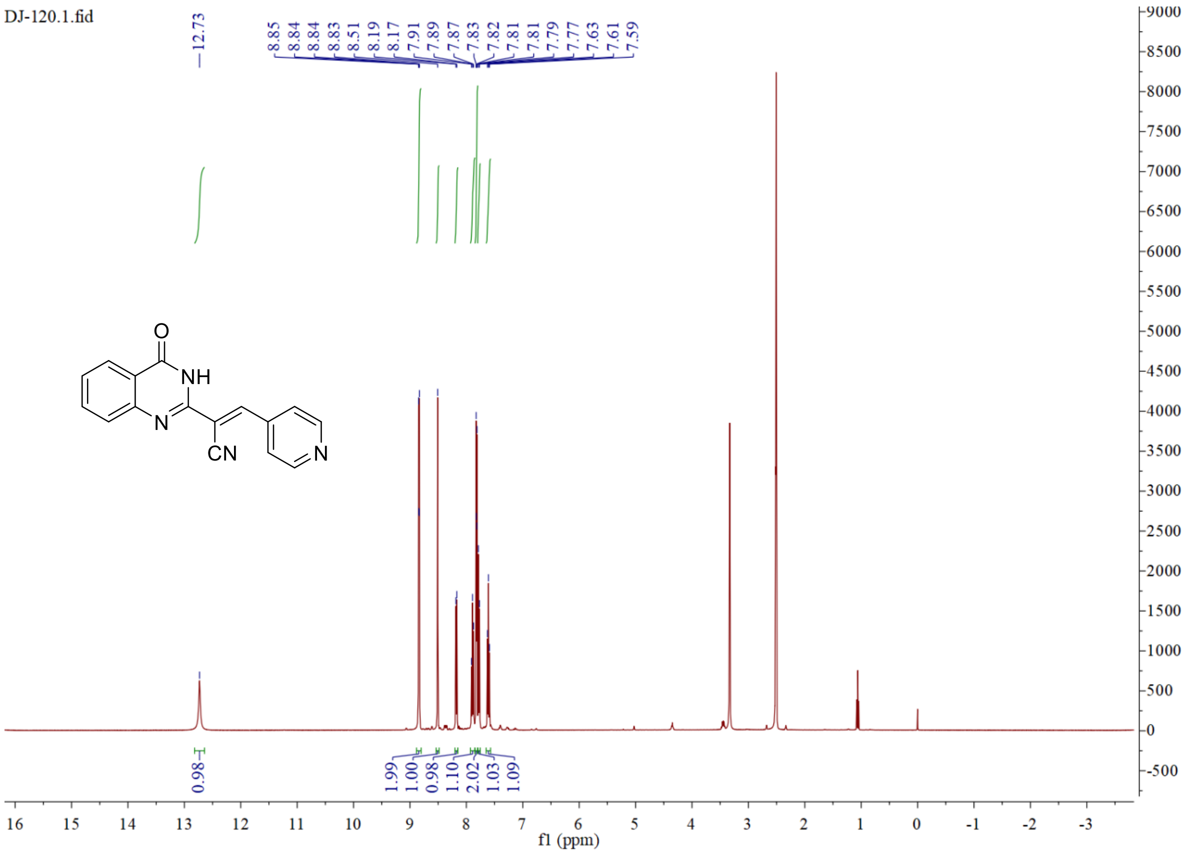

^{13}C NMR spectrum

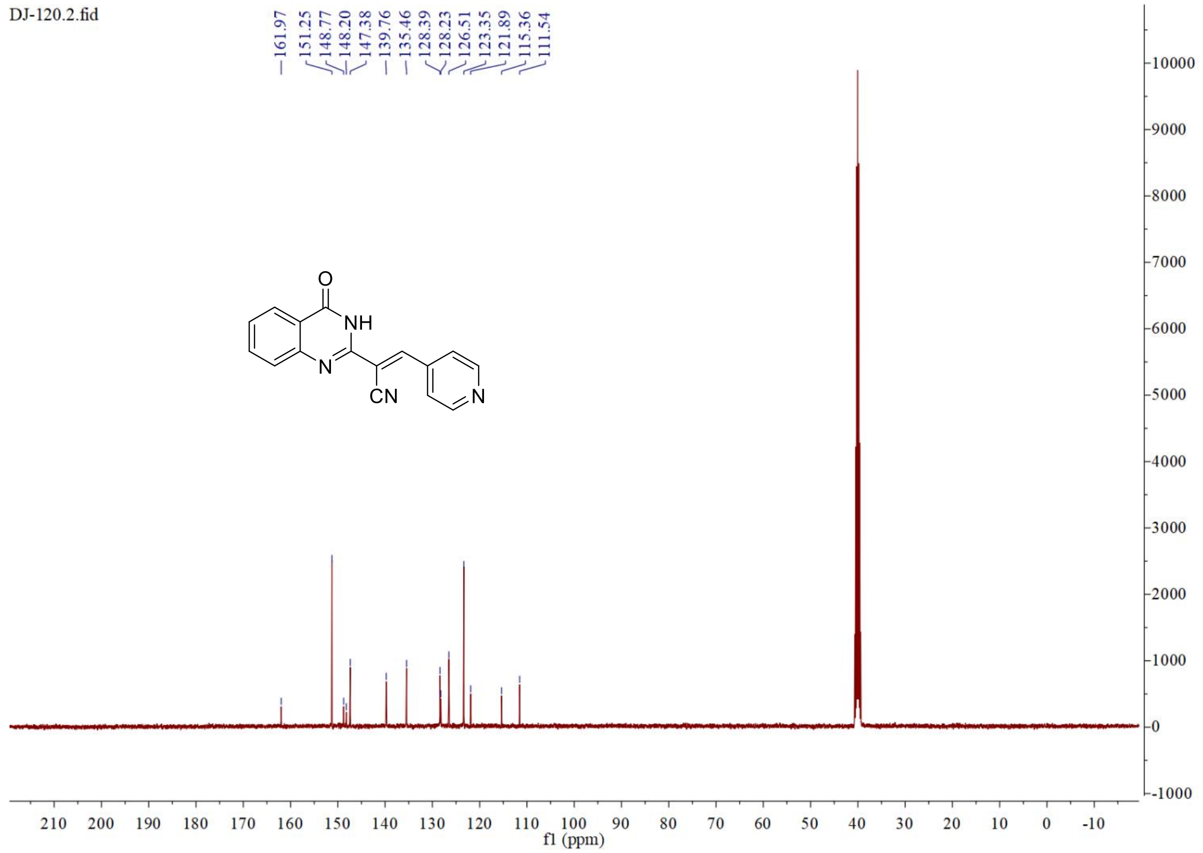

HRMS spectrum

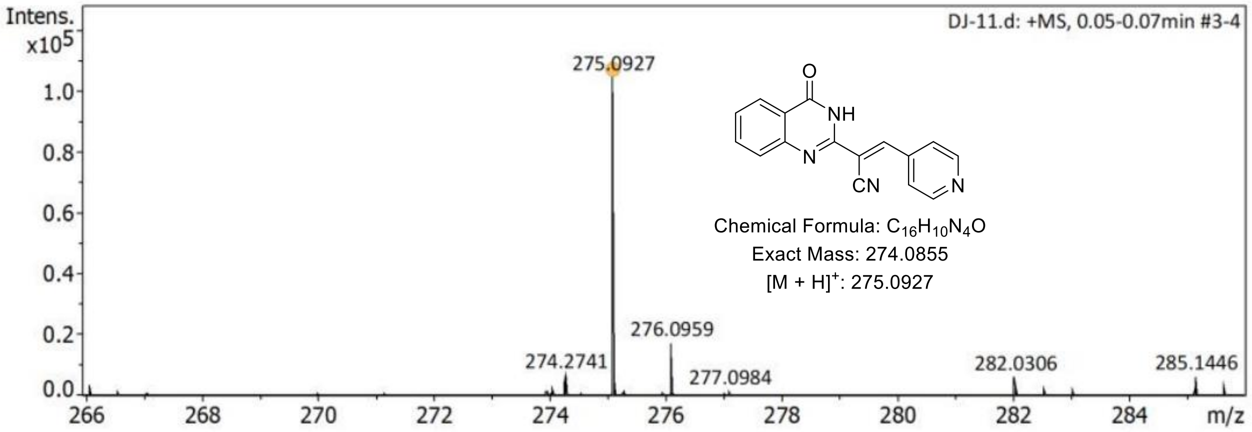

Meas. m/z	#	Ion Formula	m/z	err [ppm]	mSigma	# mSigma	Score	rdb	e¡¥ Conf	N-Rule
275.0927	1	C16H11N4O	275.0927	0.0	14.3	1	100.00	14.0	even	ok

12.13 Spectra of compound **10**

^{1}H NMR spectrum

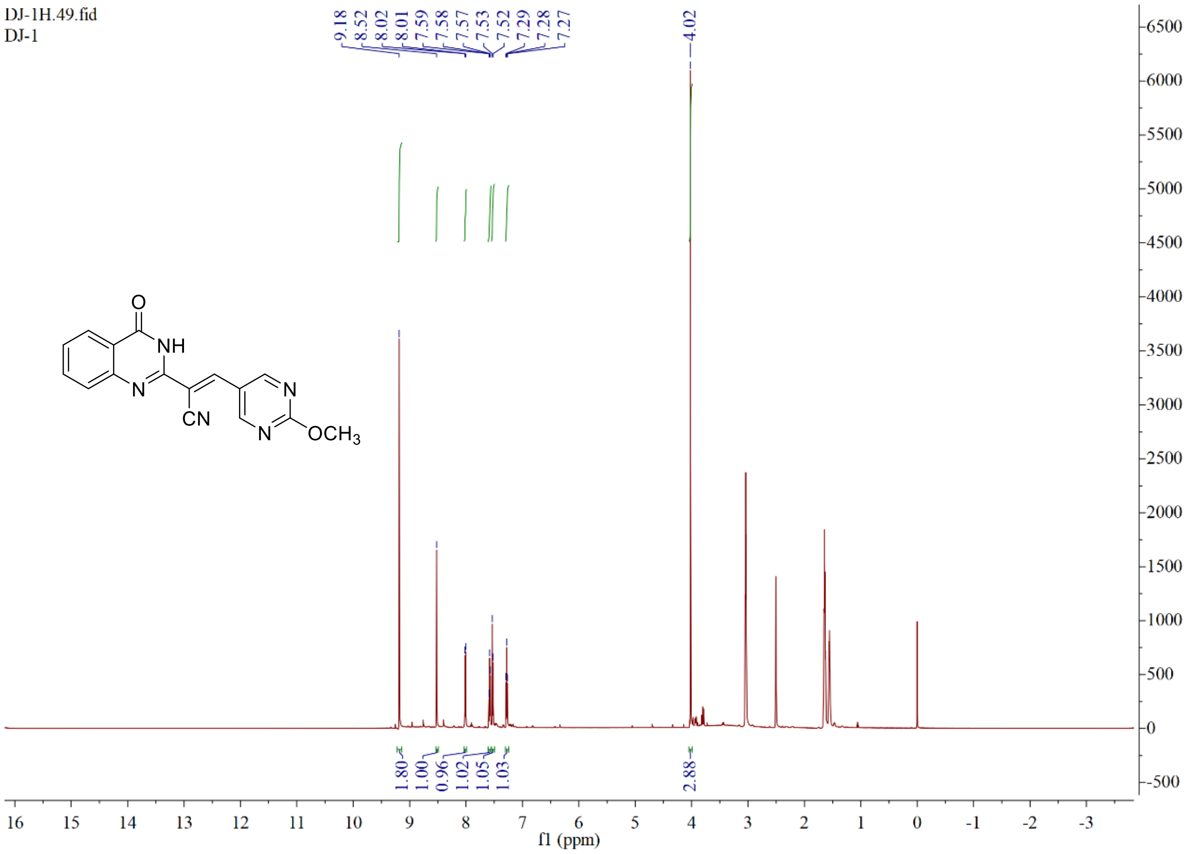

^{13}C NMR spectrum

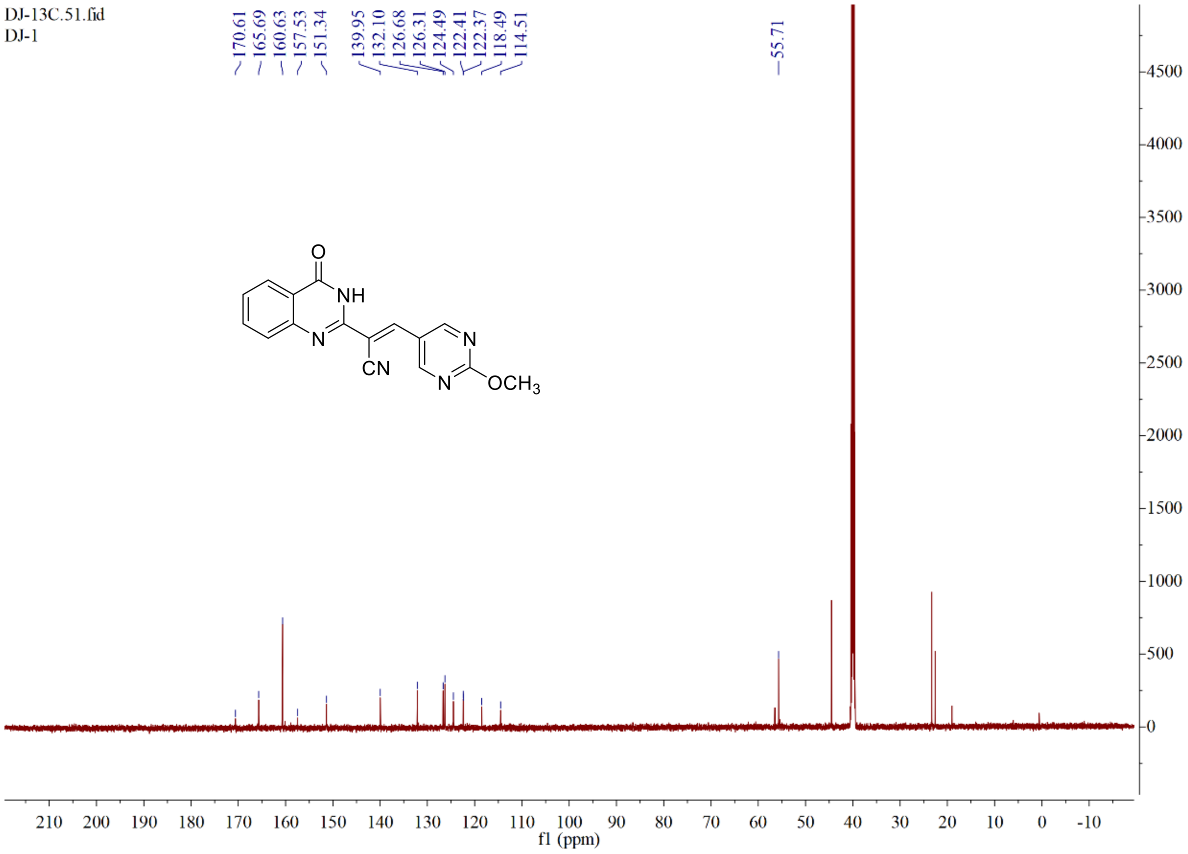

HRMS spectrum

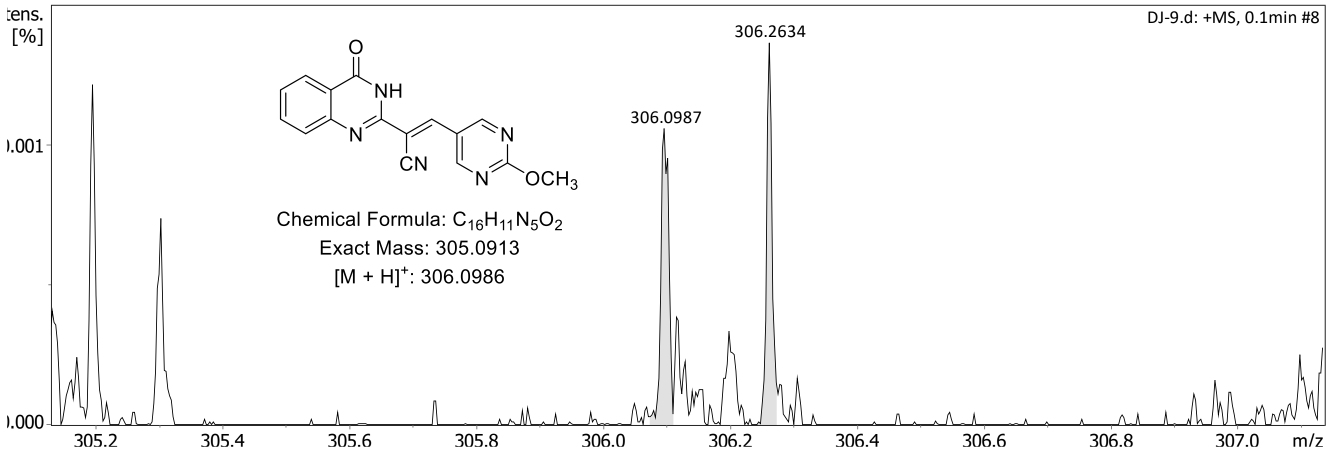

12.14 Spectra of compound **11a**

^{1}H NMR spectrum

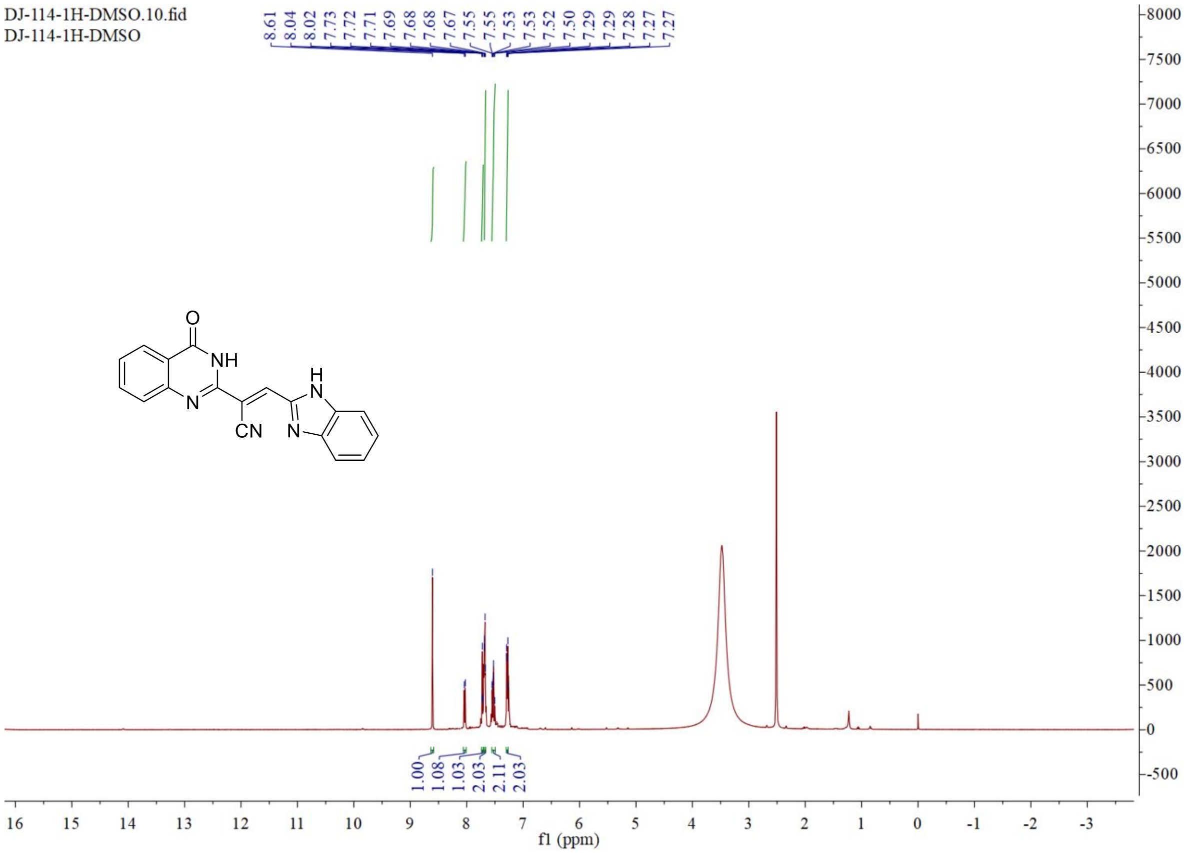

^{13}C NMR spectrum

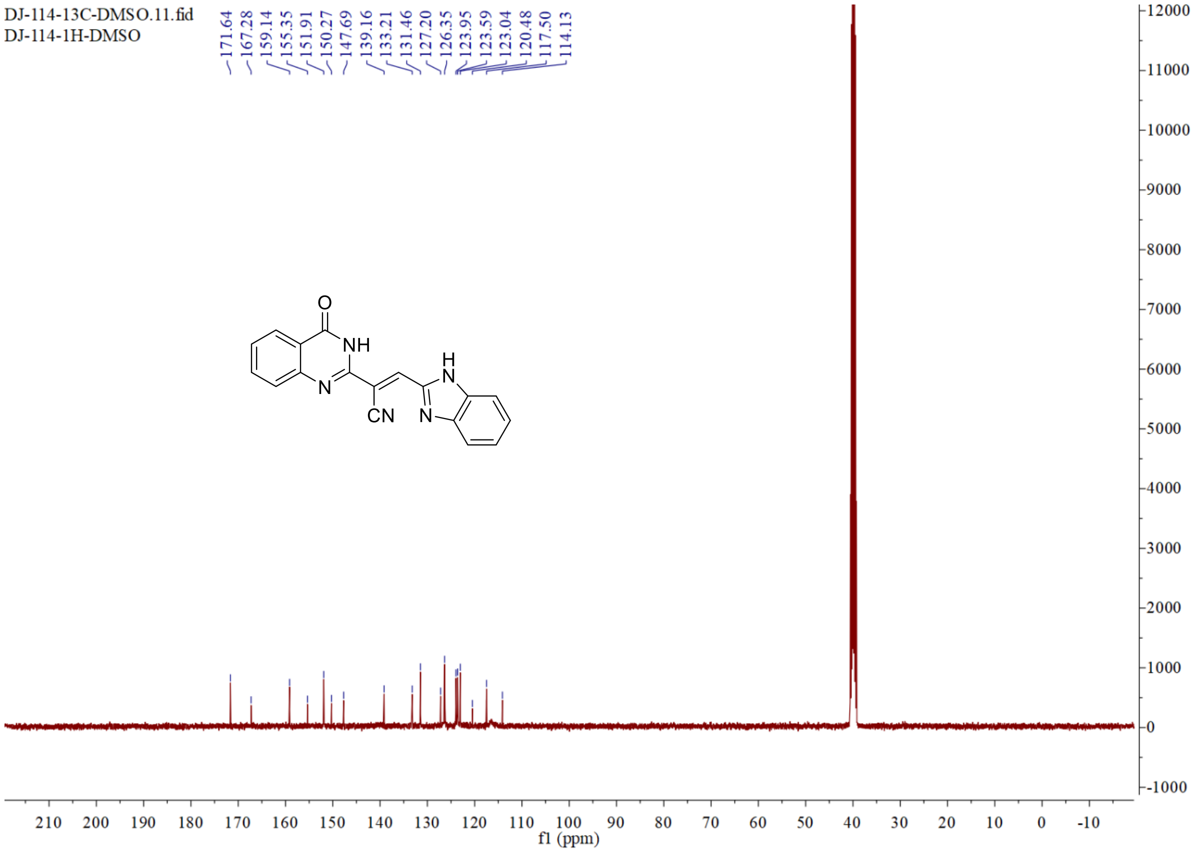

HRMS spectrum

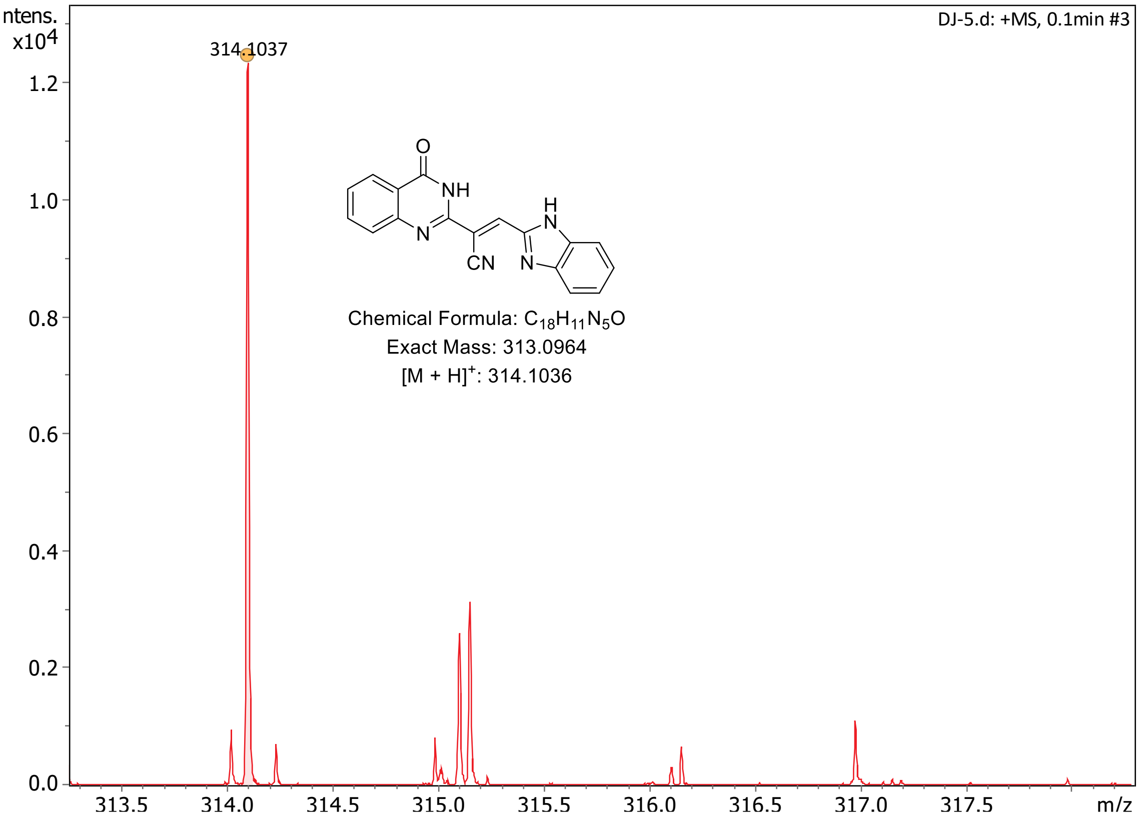

12.15 Spectra of compound **11b**

^{1}H NMR spectrum

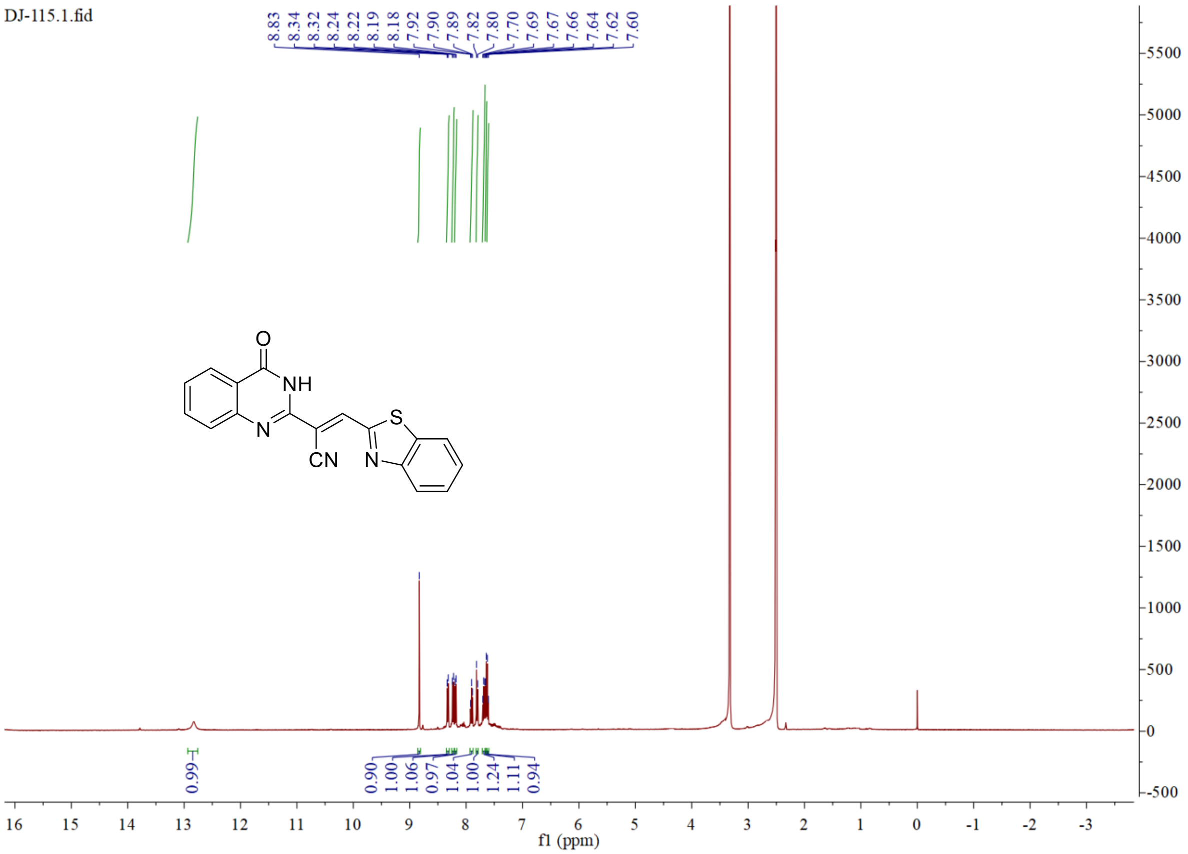

HRMS spectrum

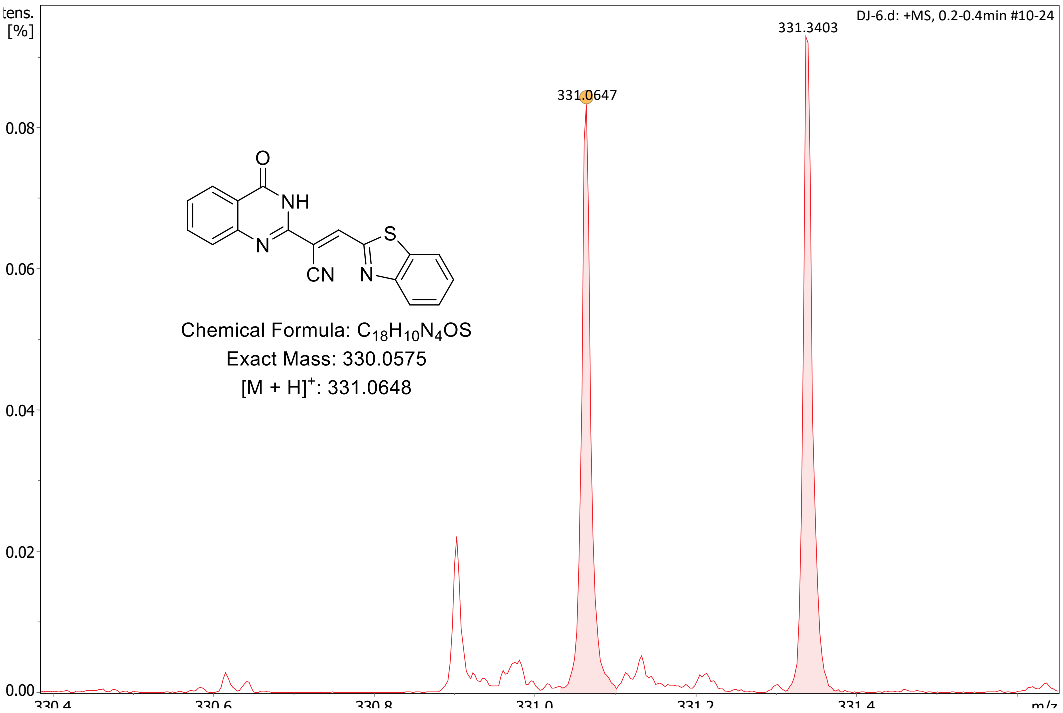

12.16 Spectra of compound **12a**

^{1}H NMR spectrum

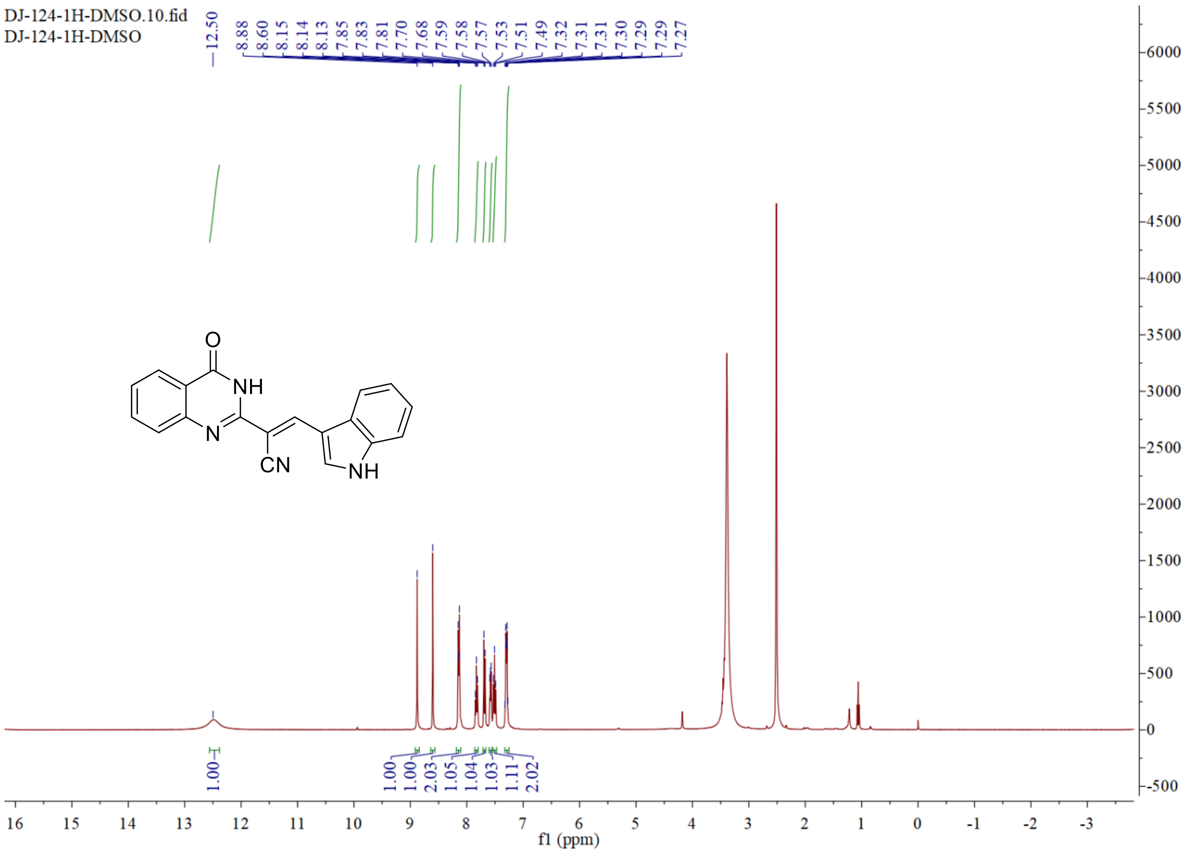

^{13}C NMR spectrum

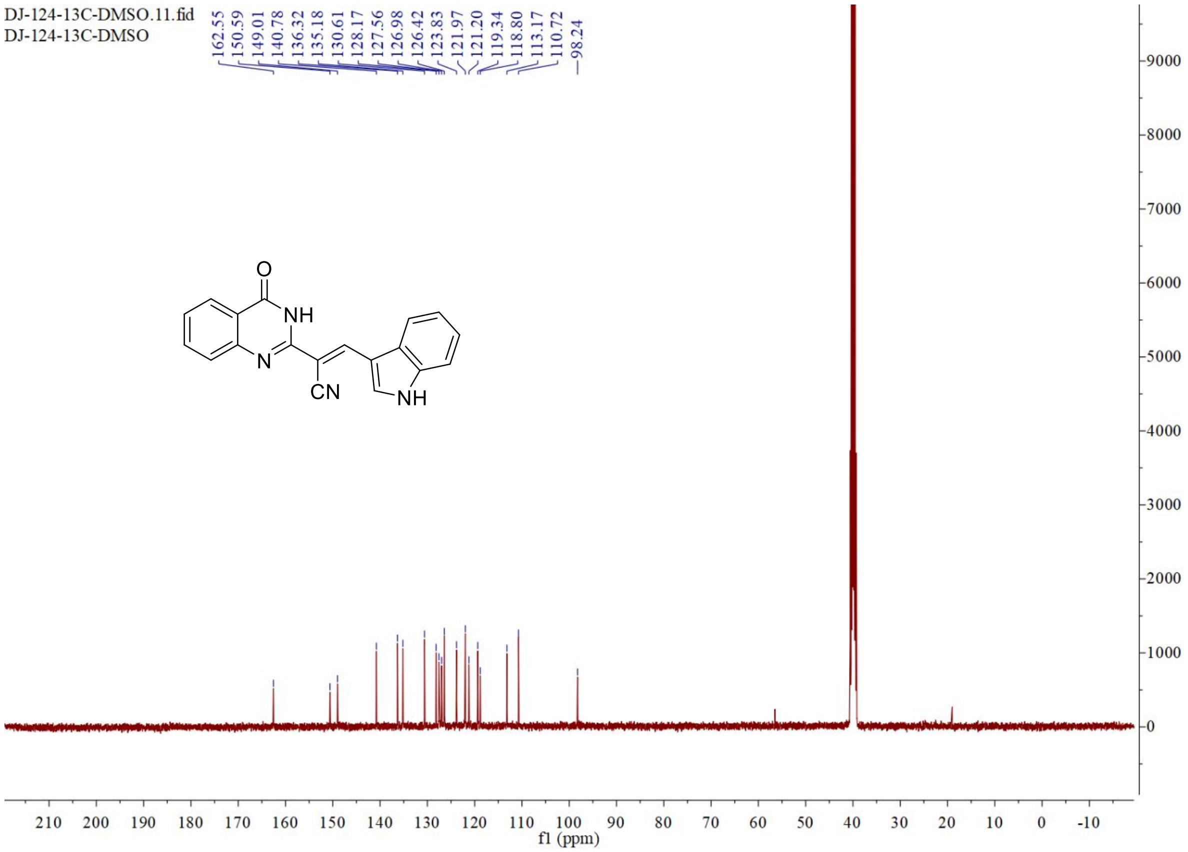

HRMS spectrum

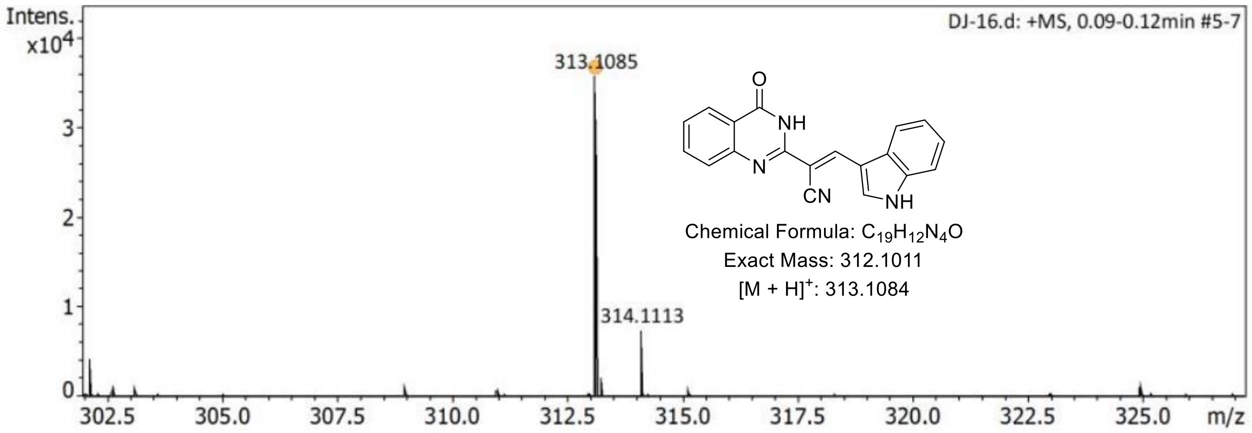

Meas. m/z	#	Ion Formula	m/z	err [ppm]	mSigma	# mSigma	Score	rdb	e¡¥ Conf	N-Rule
313.1085	1	C19H13N4O	313.1084	-0.5	16.8	1	100.00	16.0	even	ok

12.17 Spectra of compound **12b**

^{1}H NMR spectrum

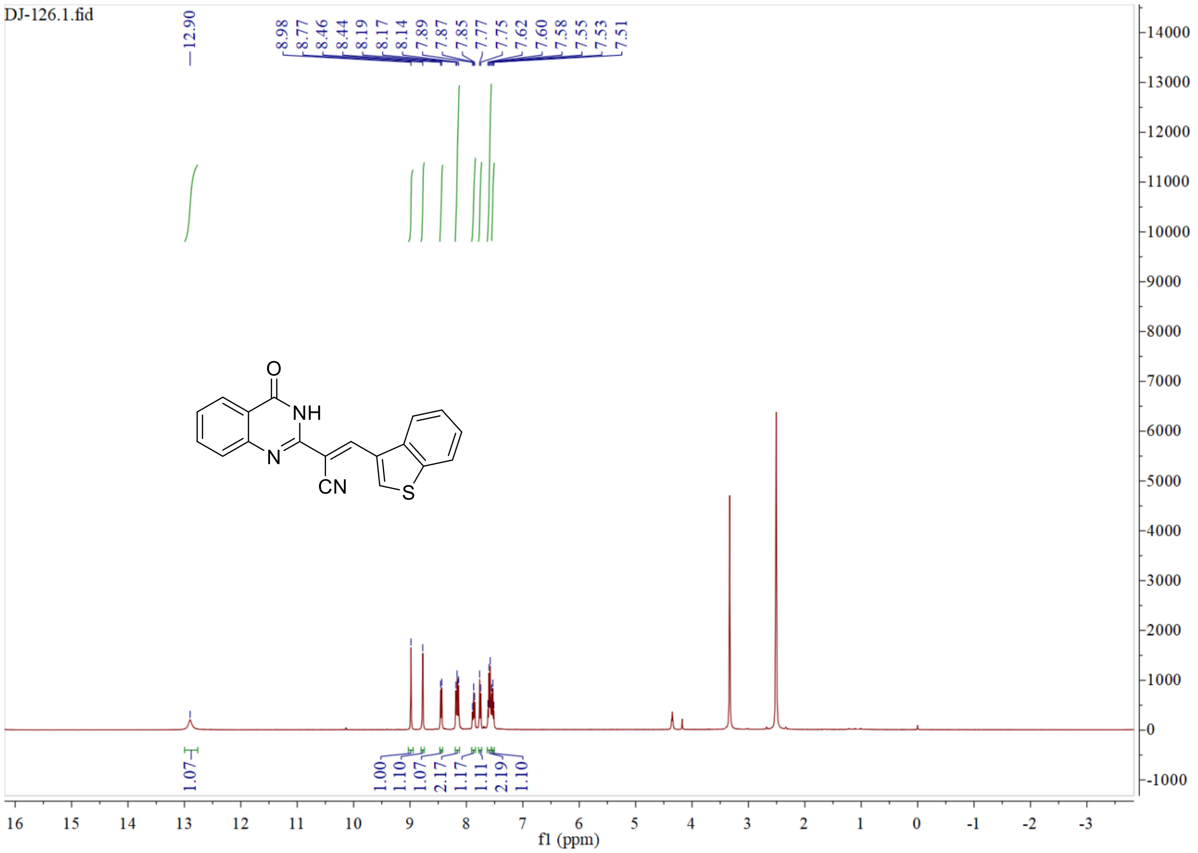

^{13}C NMR spectrum

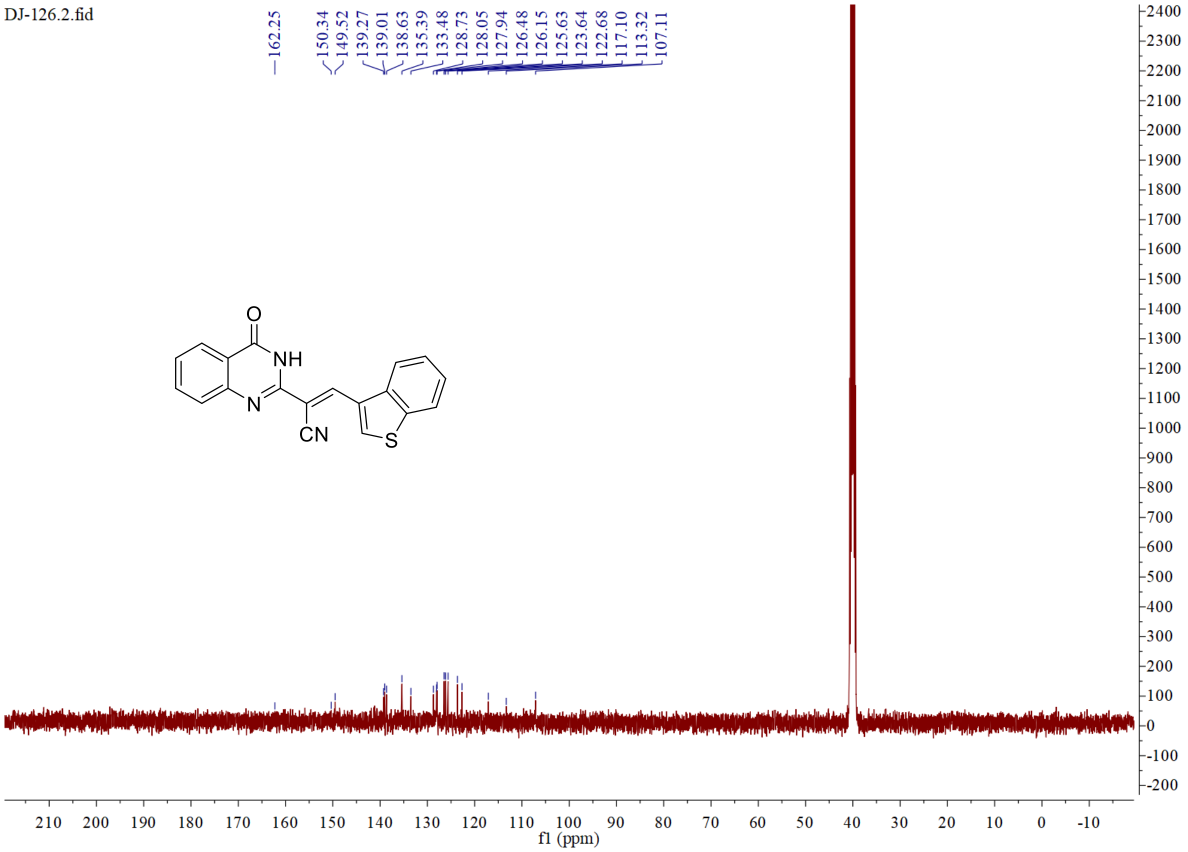

HRMS spectrum

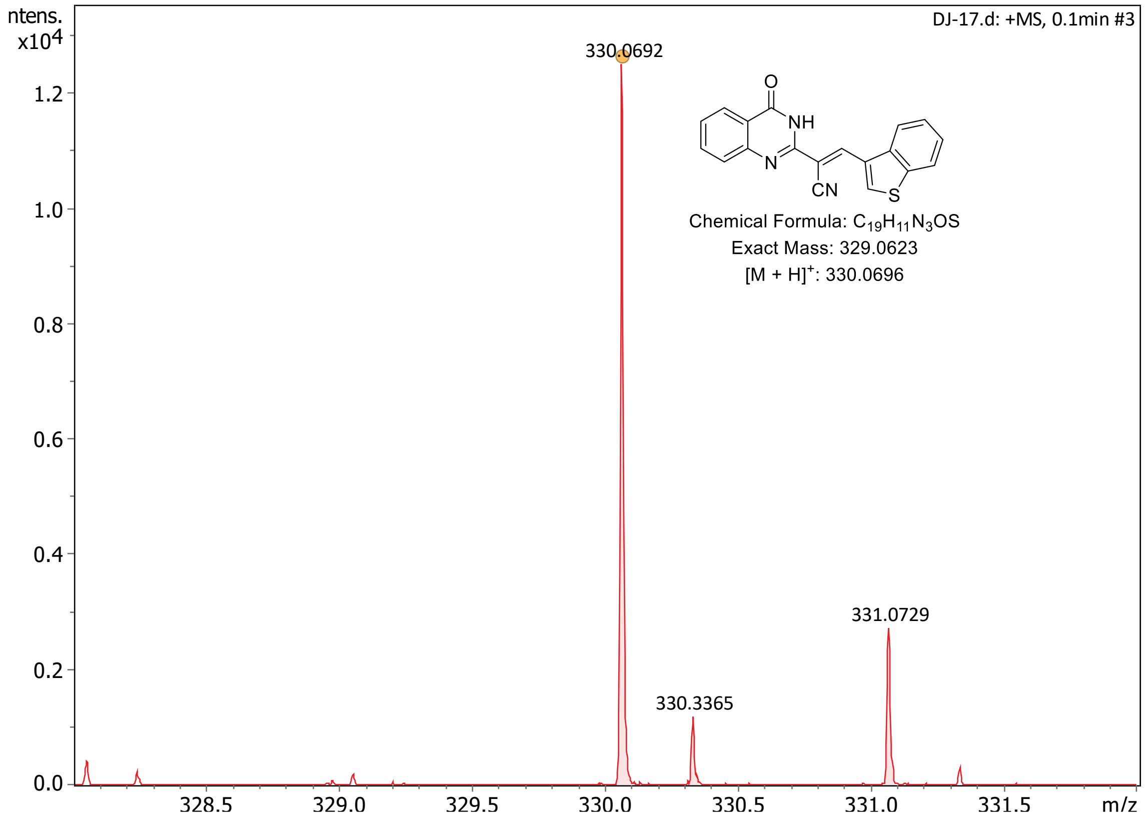

12.18 Spectra of compound **12c**

^{1}H NMR spectrum

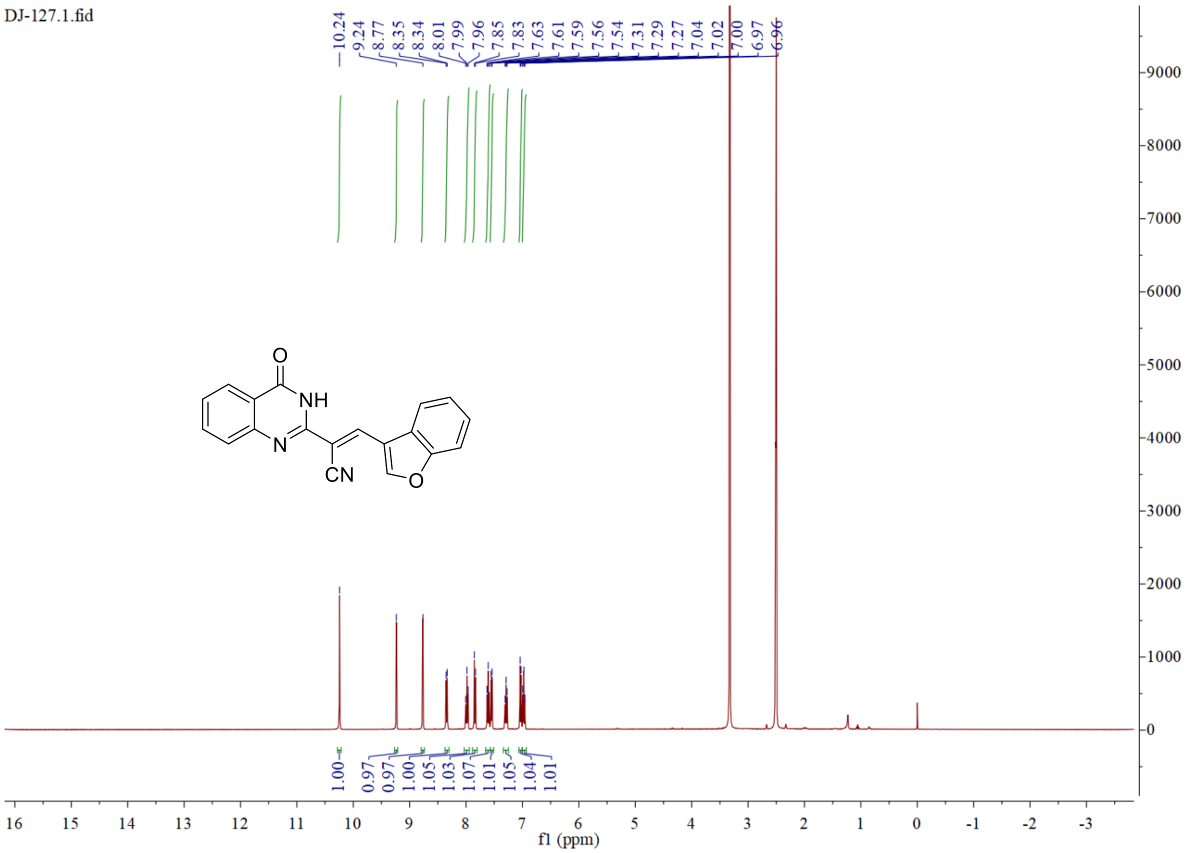

^{13}C NMR spectrum

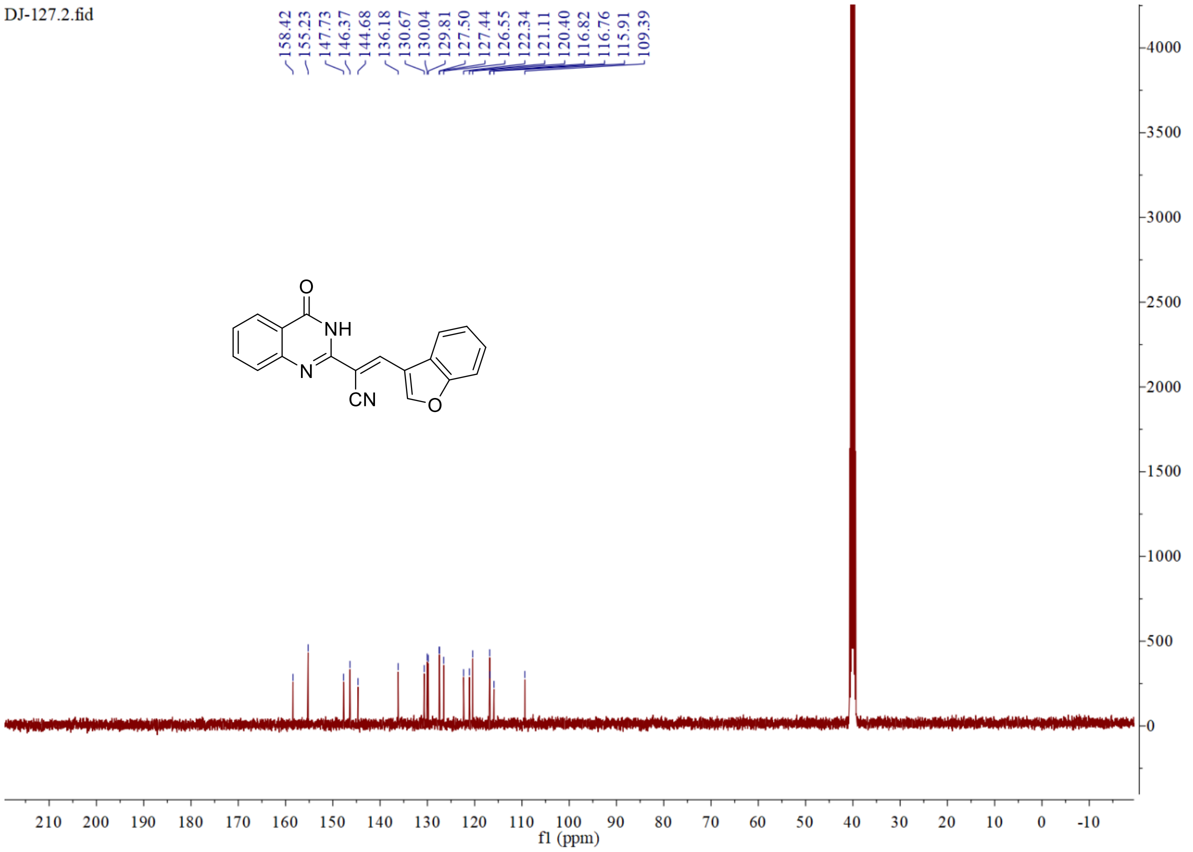

HRMS spectrum

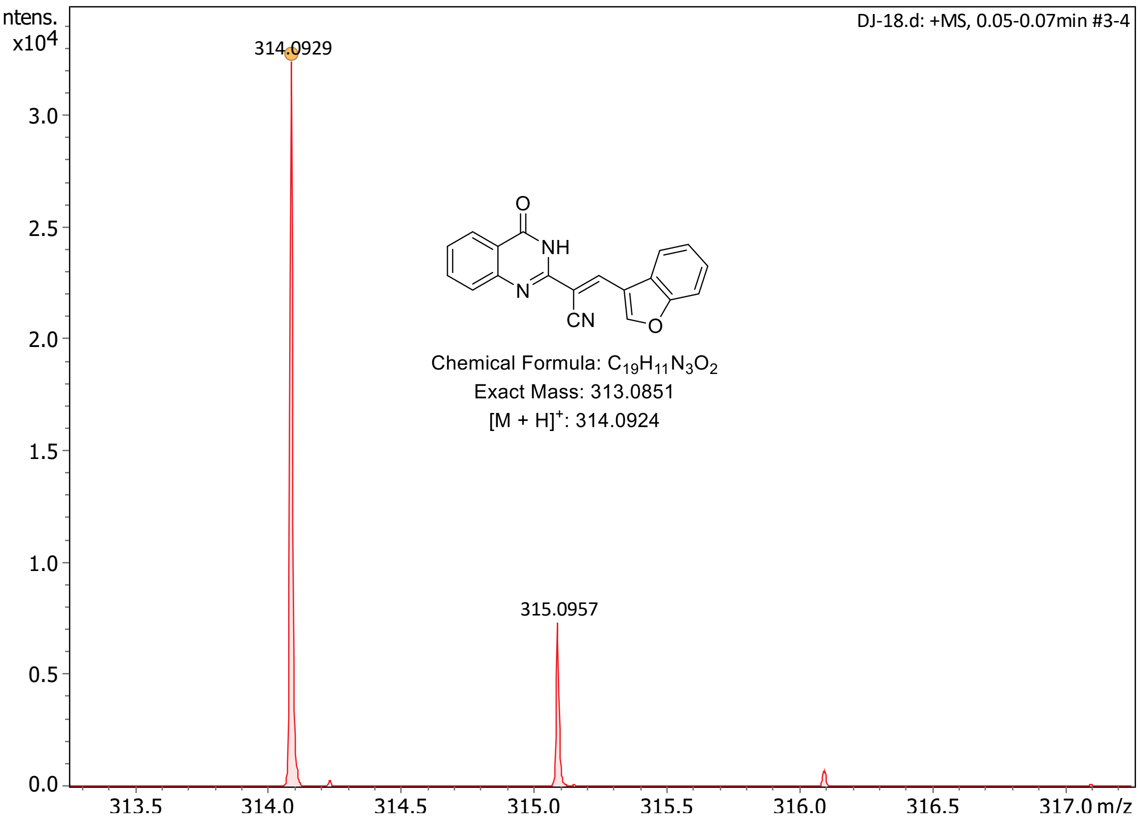

12.19 Spectra of compound **13a**

^{1}H NMR spectrum

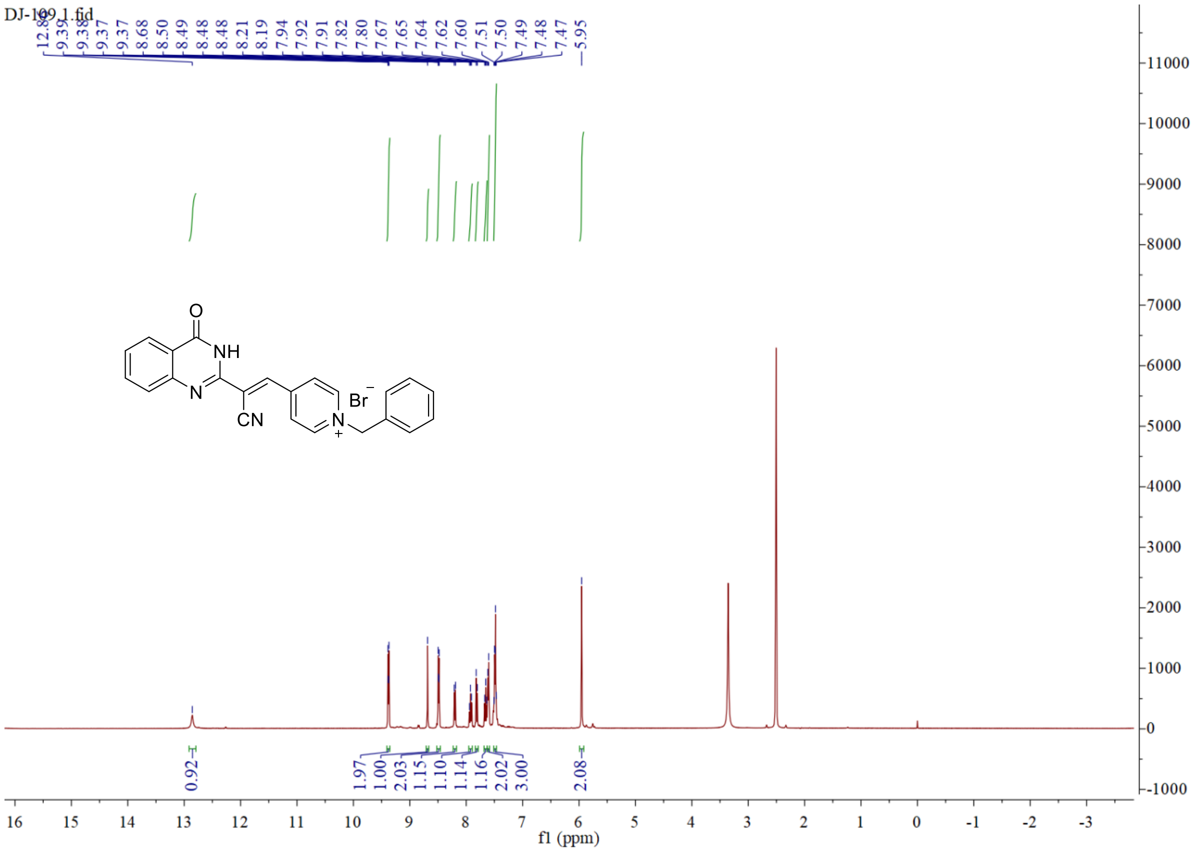

^{13}C NMR spectrum

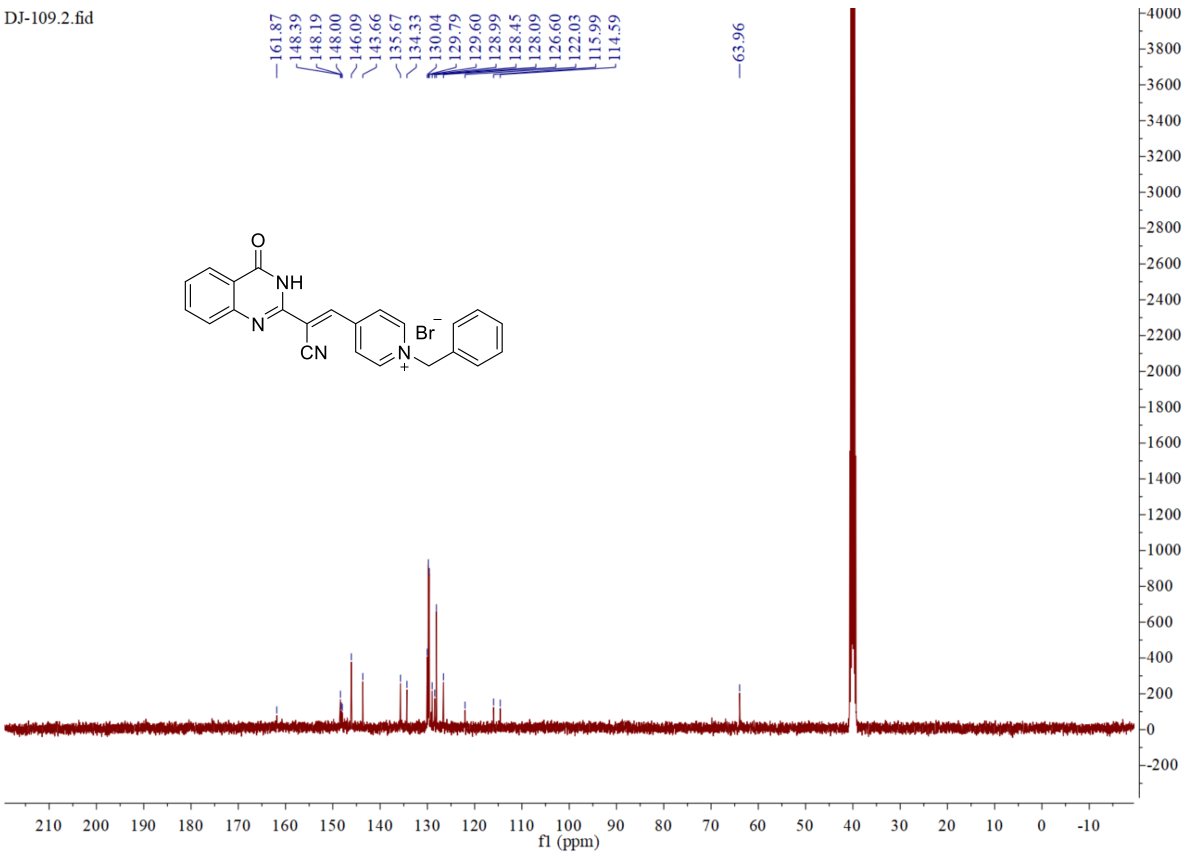

HRMS spectrum

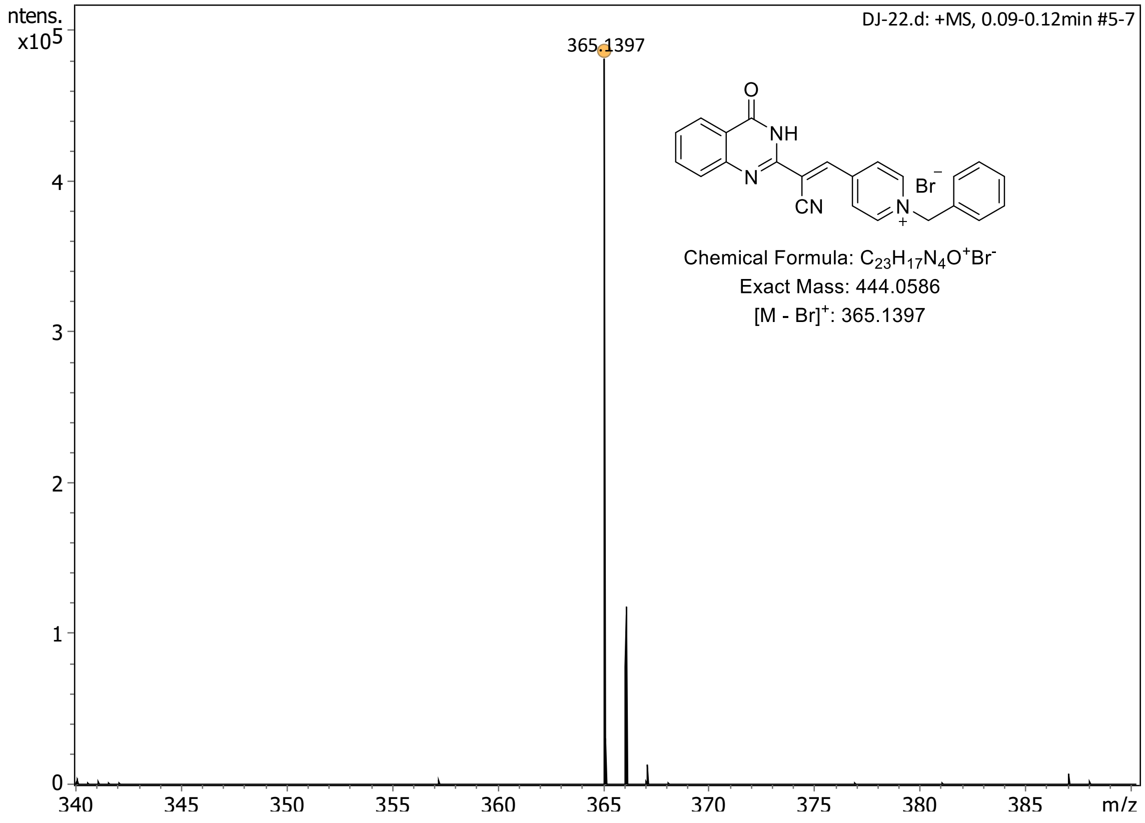

12.20 Spectra of compound **13b**

^{1}H NMR spectrum

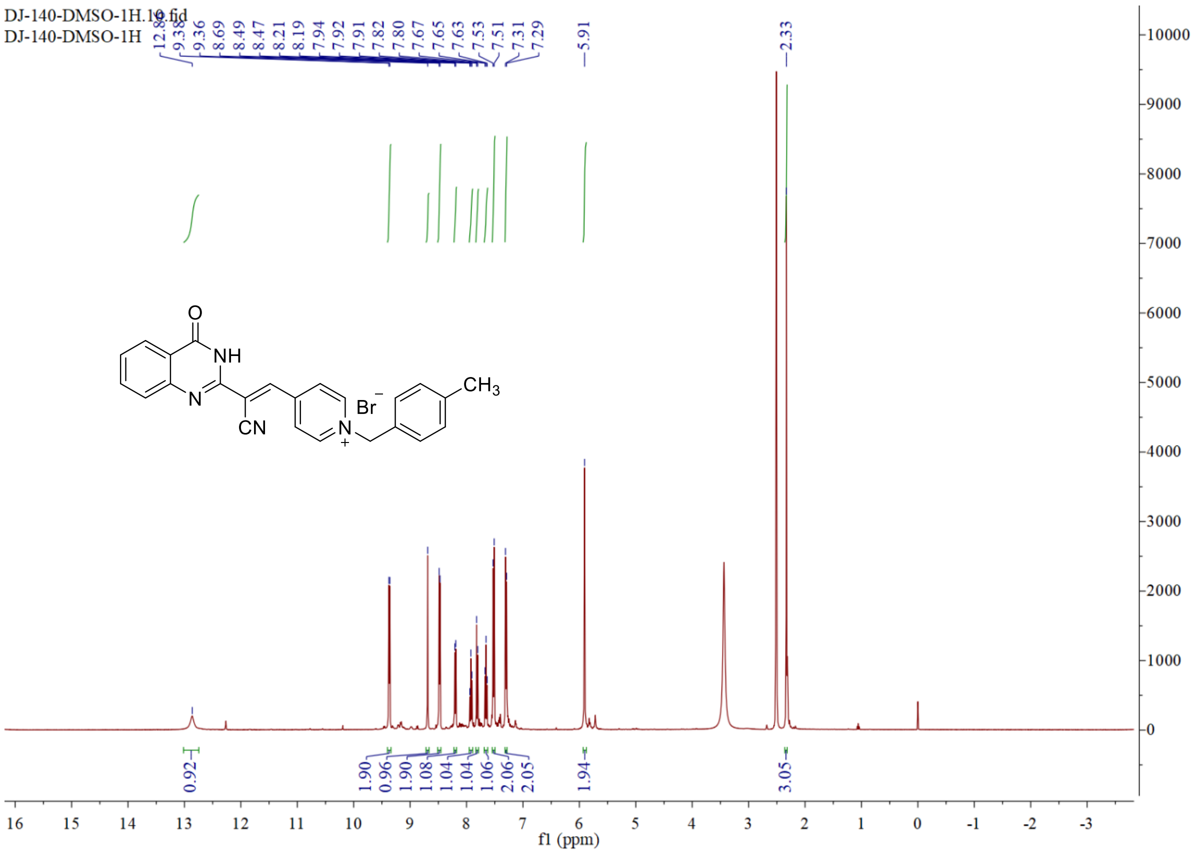

^{13}C NMR spectrum

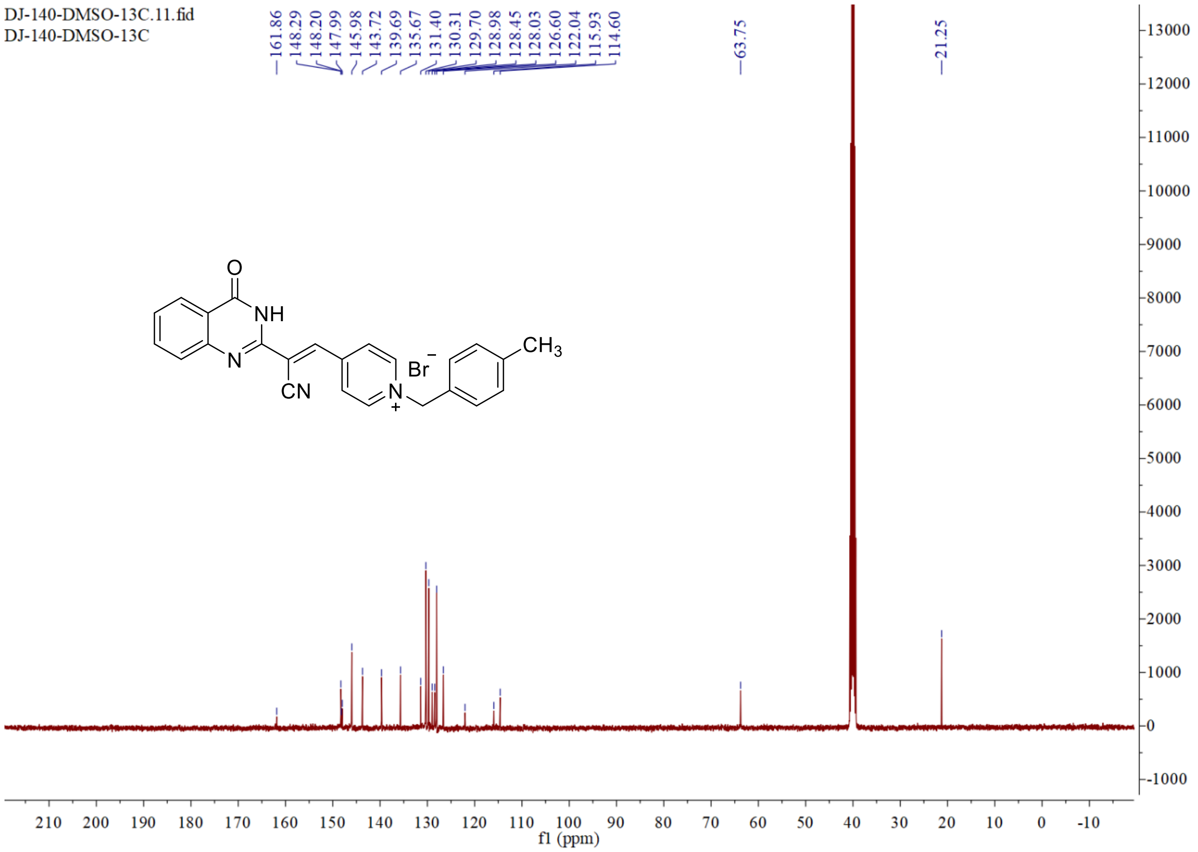

HRMS spectrum

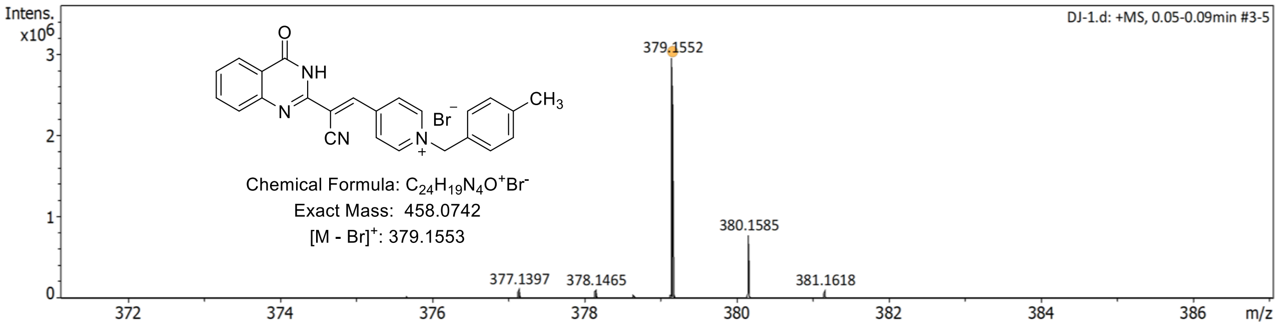

Meas. m/z	#	Ion Formula	m/z	err [ppm]	mSigma	# mSigma	Score	rdb	e¡¥ Conf	N-Rule
379.1552	1	C24H19N4O	379.1553	0.3	8.8	1	100.00	18.0	even	ok

12.21 Spectra of compound **13c**

^{1}H NMR spectrum

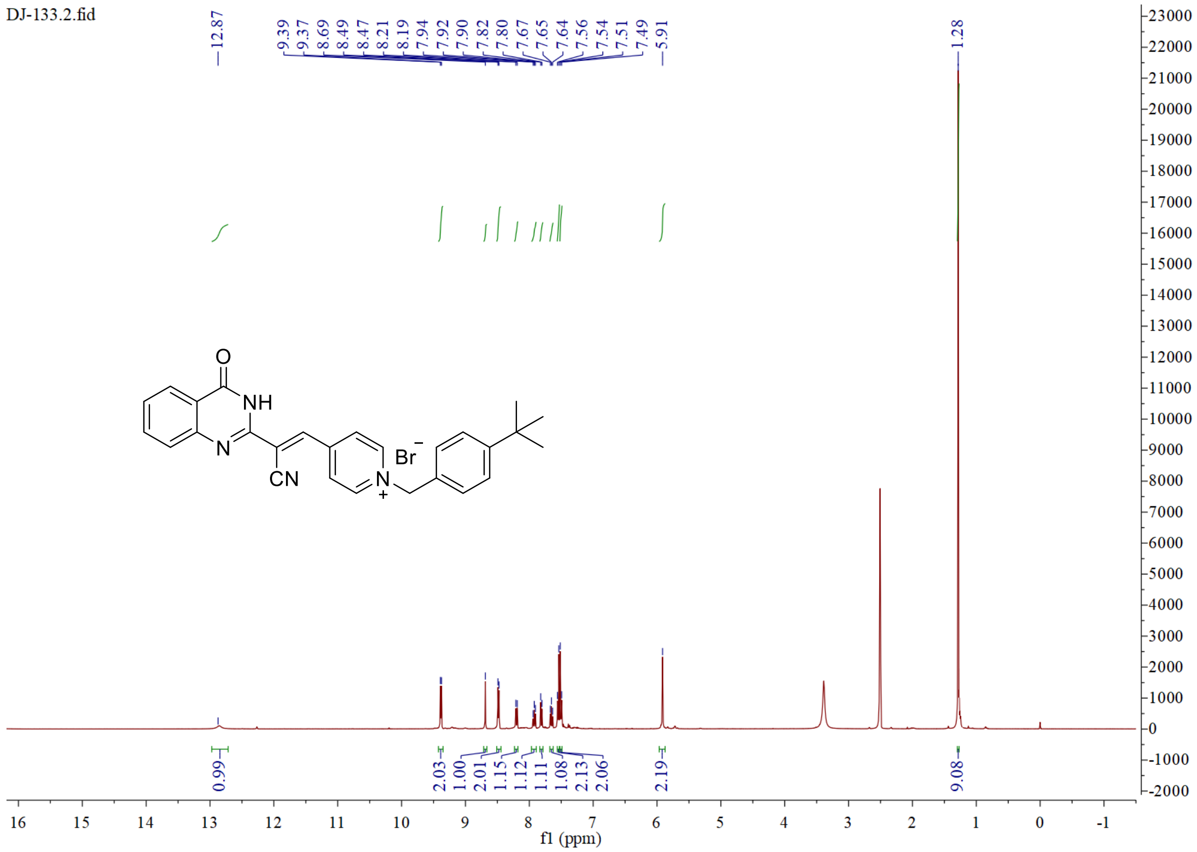

^{13}C NMR spectrum

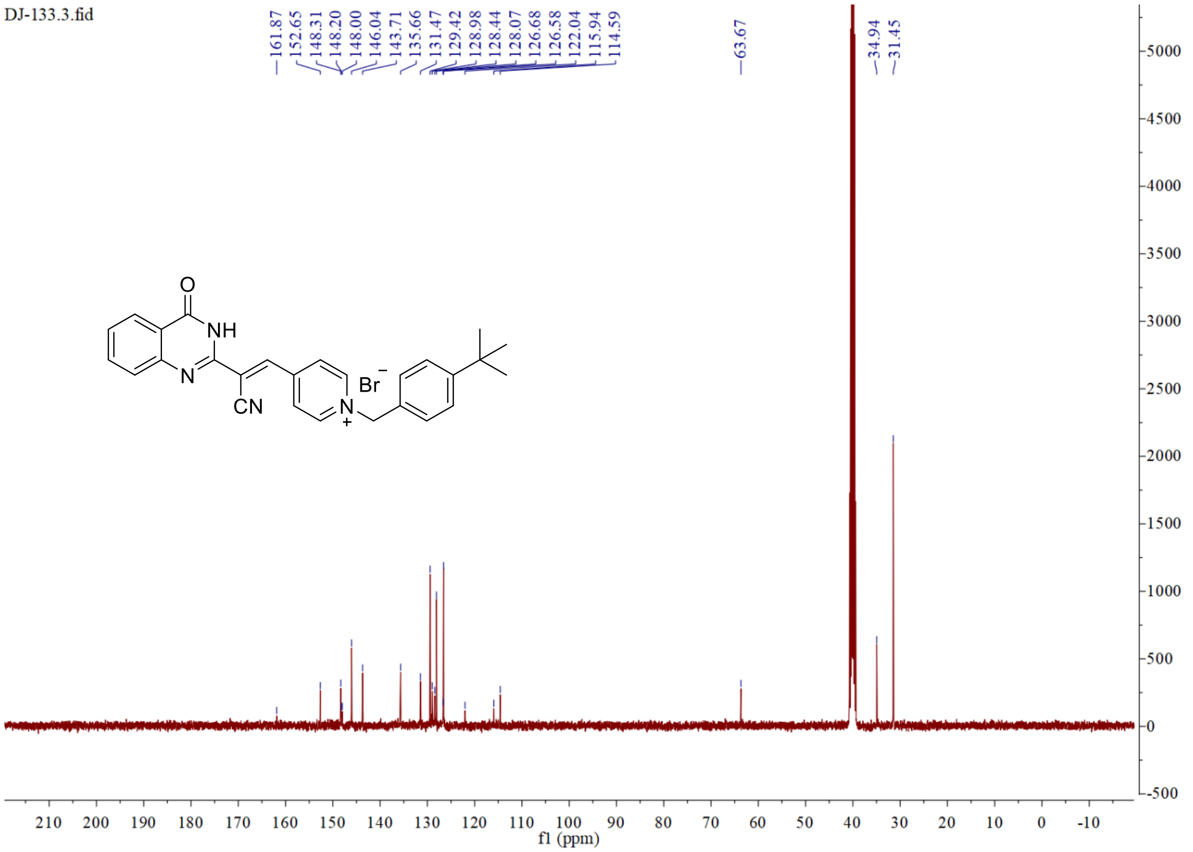

HRMS spectrum

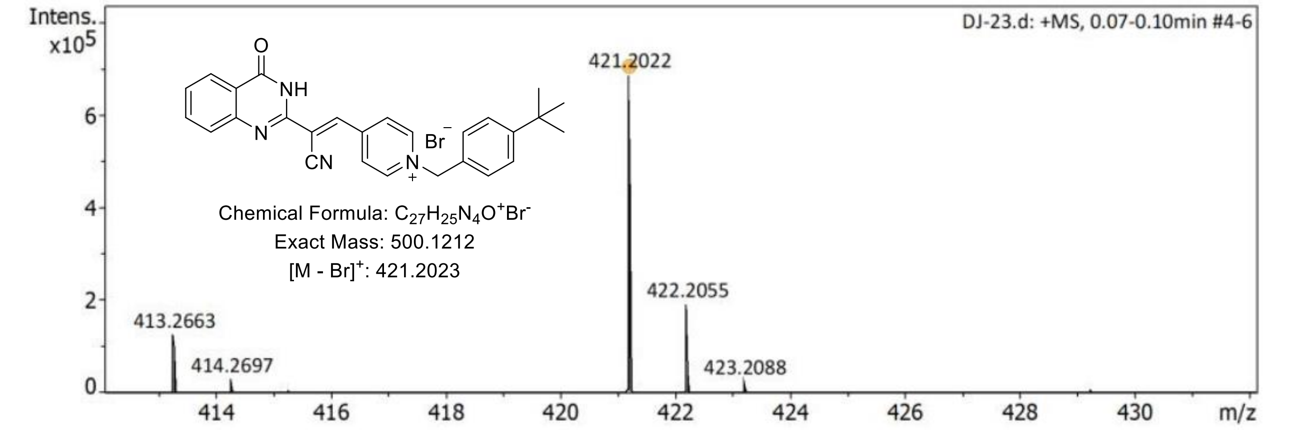

Meas. m/z	#	Ion Formula	m/z	err [ppm]	mSigma	# mSigma	Score	rdb	e¡¥ Conf	N-Rule
421.2022	1	C27H25N4O	421.2023	0.2	18.7	1	100.00	18.0	even	ok

12.22 Spectra of compound **13d**

^{1}H NMR spectrum

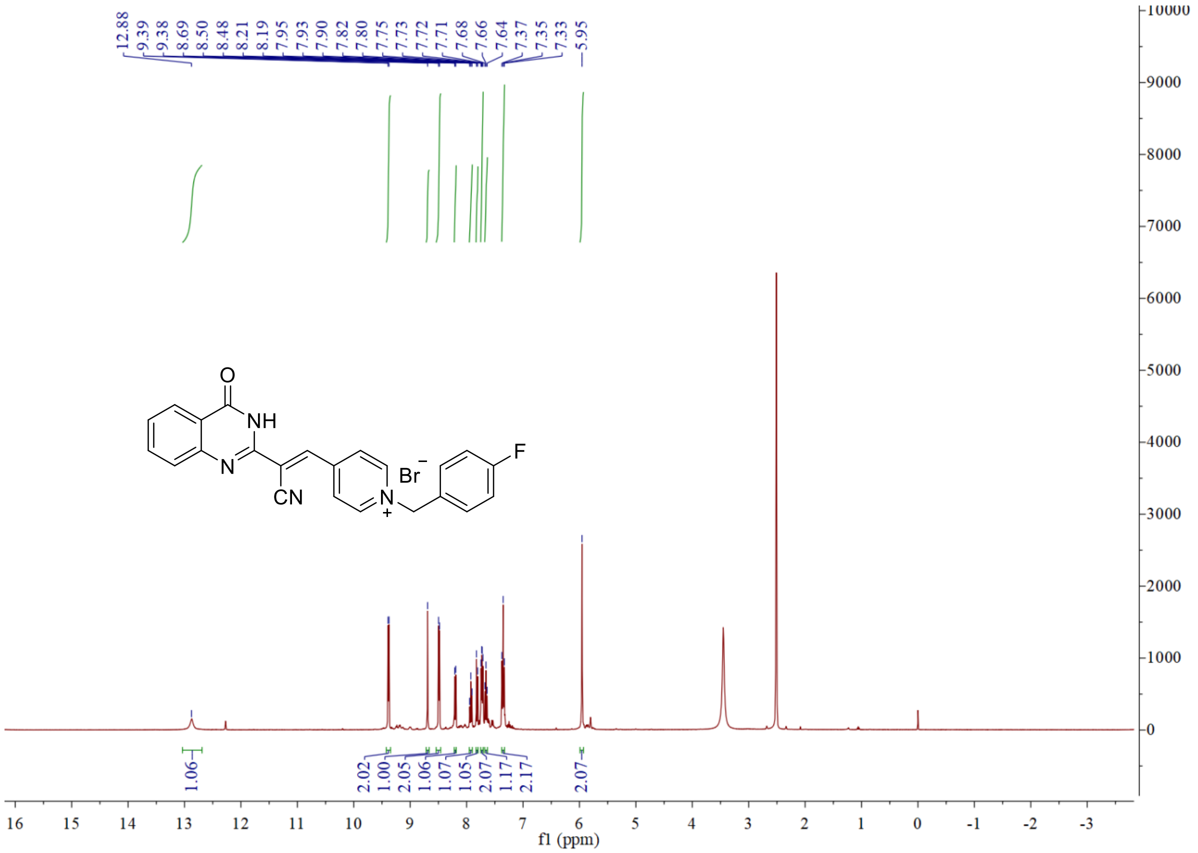

^{13}C NMR spectrum

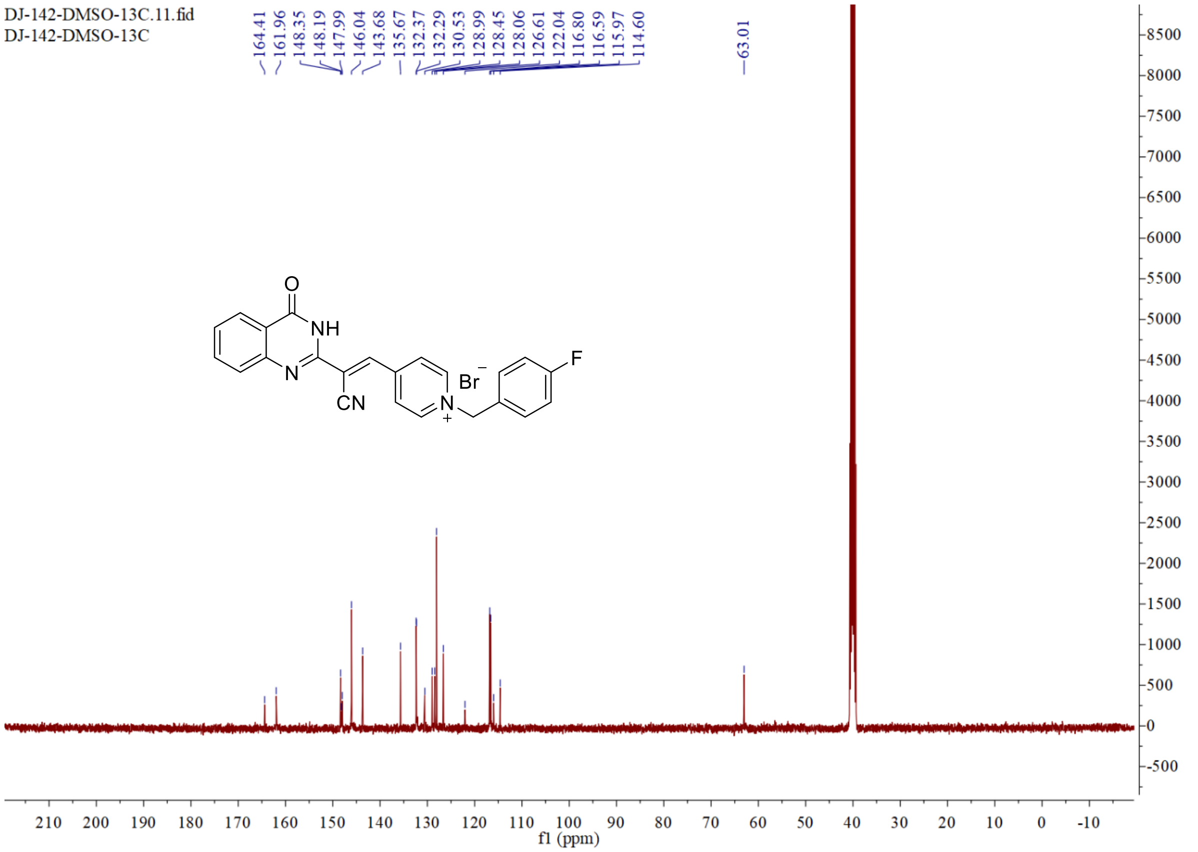

HRMS spectrum

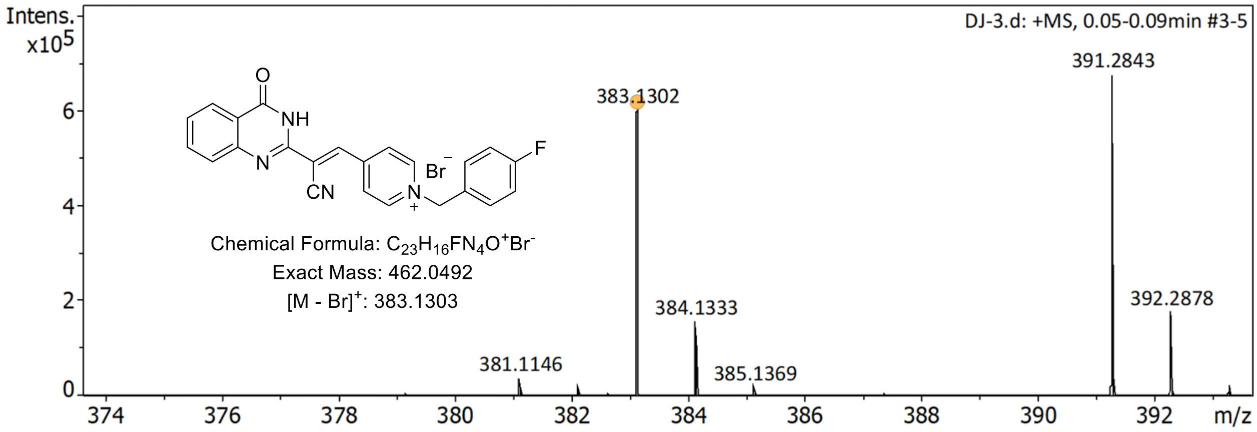

Meas. m/z	#	Ion Formula	m/z	err [ppm]	mSigma	# mSigma	Score	rdb	e¡¥ Conf	N-Rule
383.1302	1	C23H16FN4O	383.1303	0.2	3.5	1	100.00	18.0	even	ok

12.23 Spectra of compound **13e**

^{1}H NMR spectrum

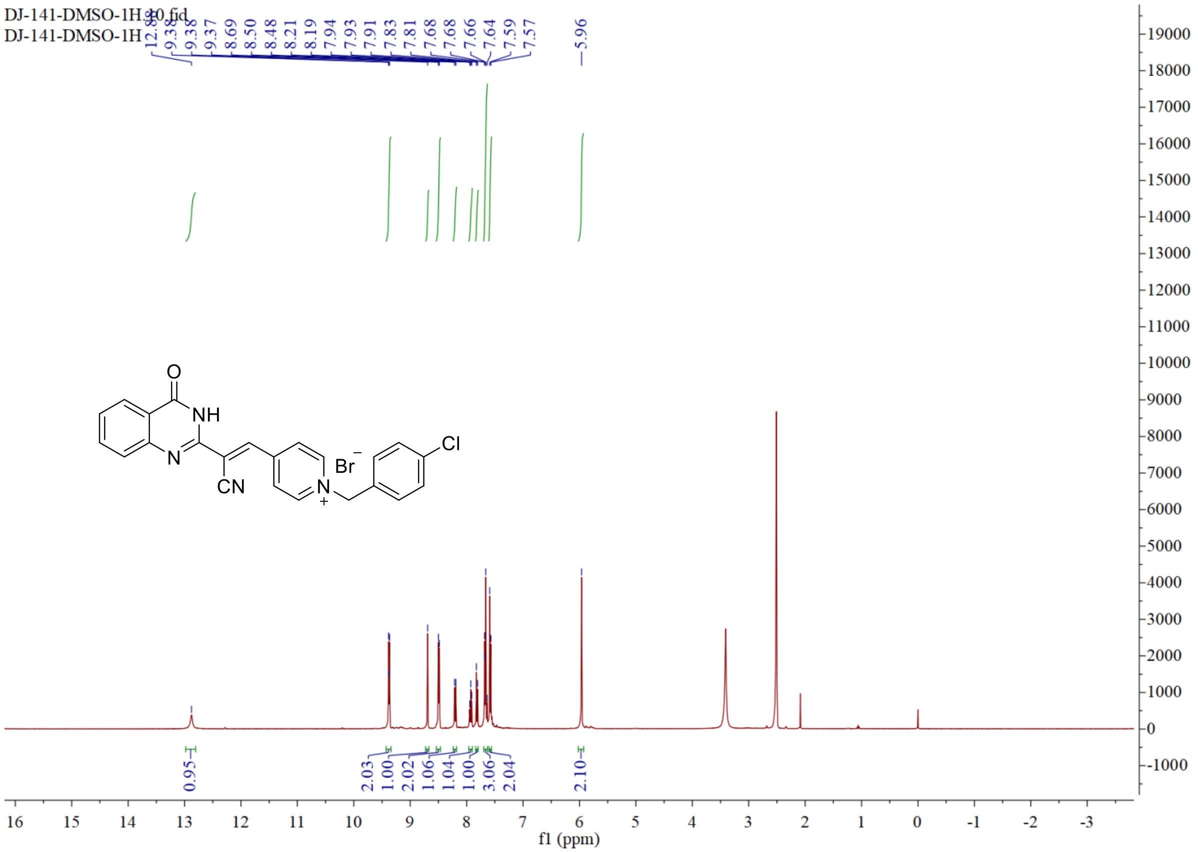

^{13}C NMR spectrum

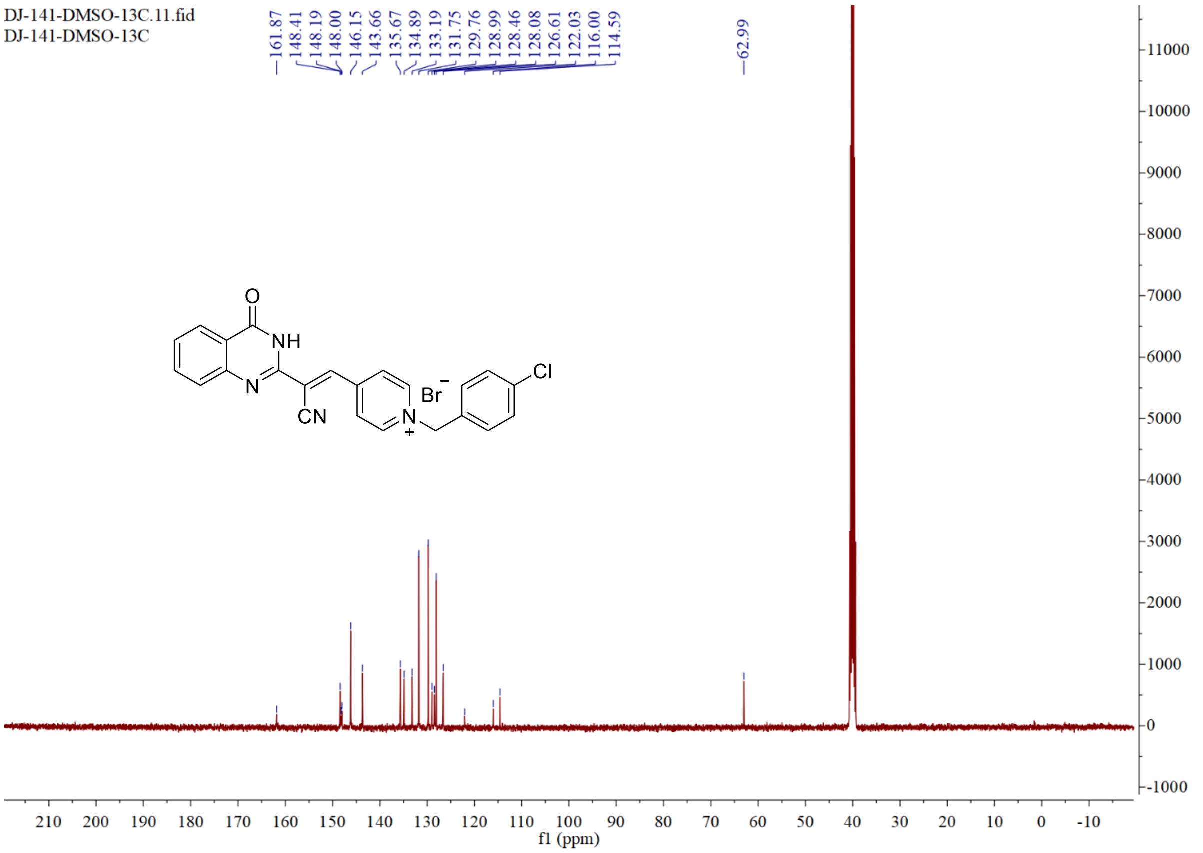

HRMS spectrum

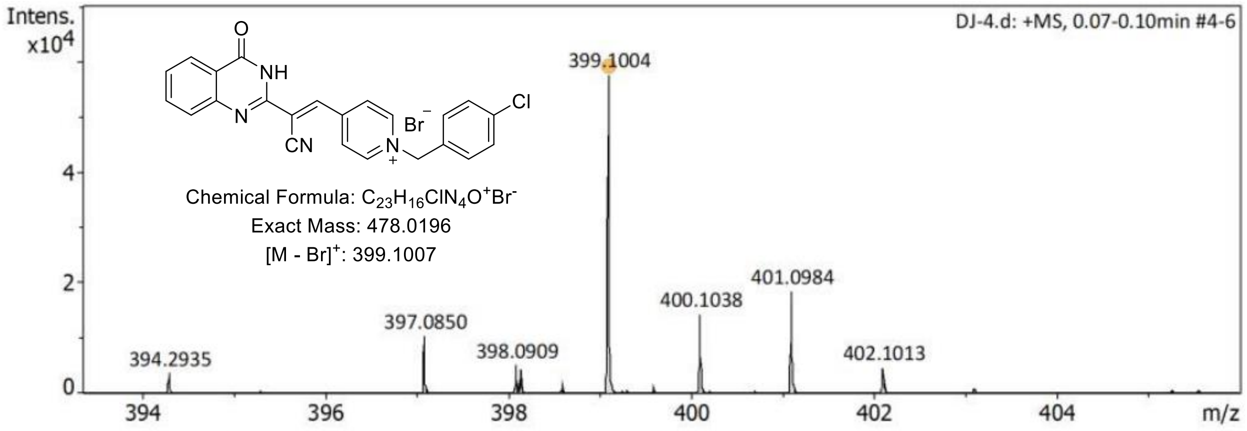

Meas. m/z	#	Ion Formula	m/z	err [ppm]	mSigma	# mSigma	Score	rdb	e¡¥ Conf	N-Rule
399.1004	1	C23H16ClN4O	399.1007	0.7	17.7	1	100.00	21.0	even	ok

12.24 Spectra of compound **13f**

^{1}H NMR spectrum

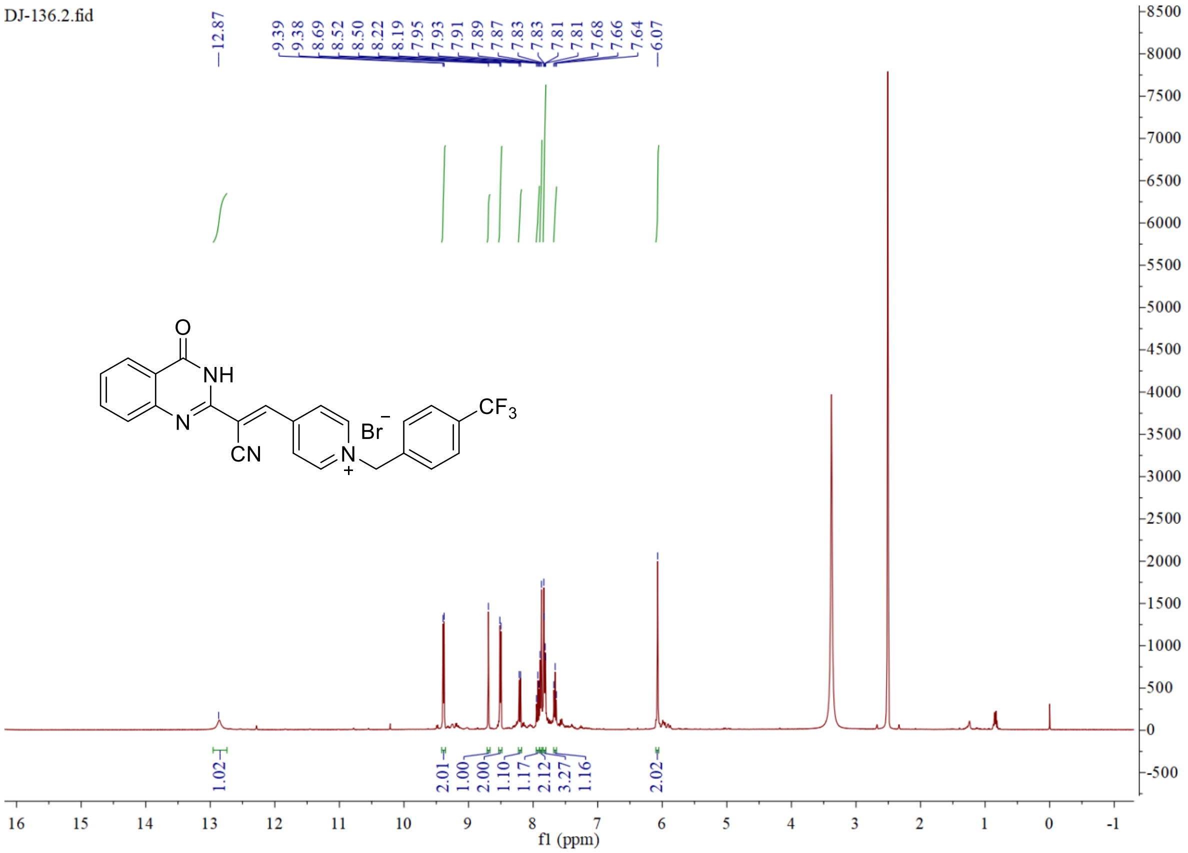

^{13}C NMR spectrum

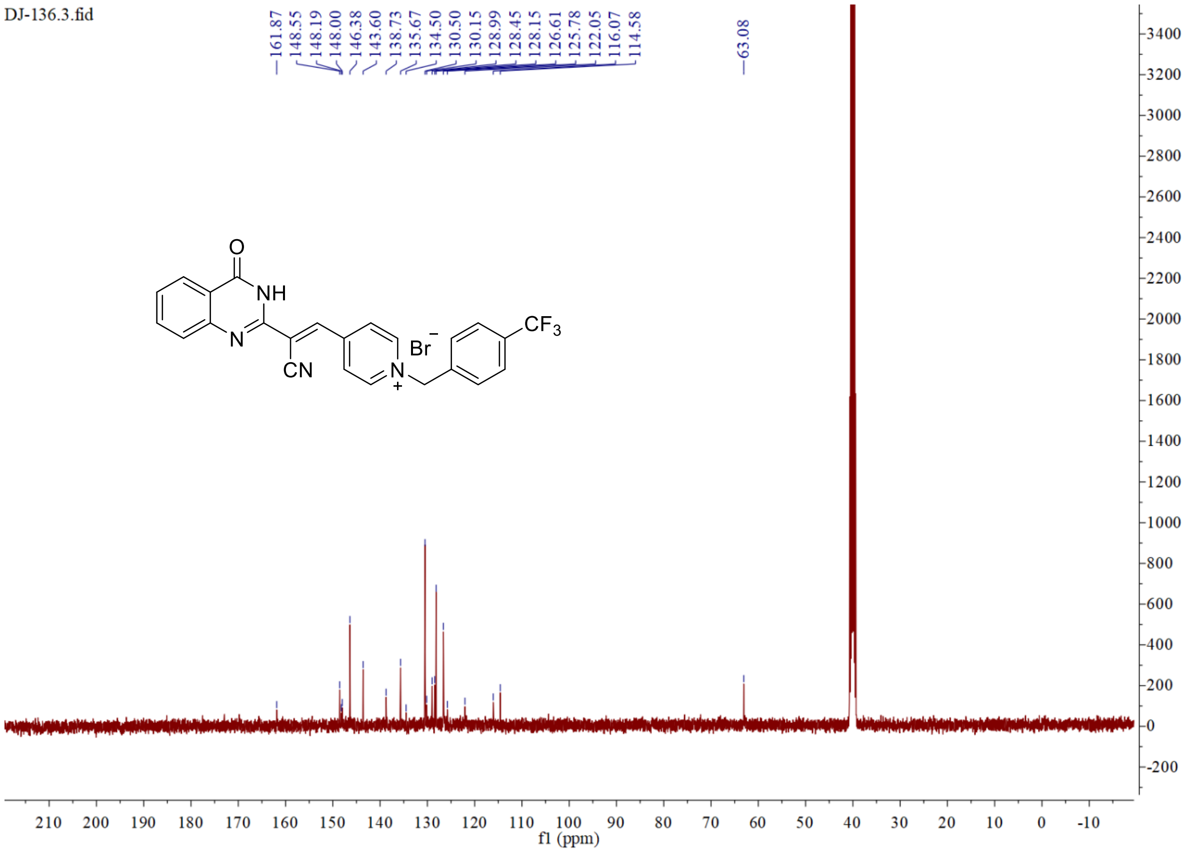

HRMS spectrum

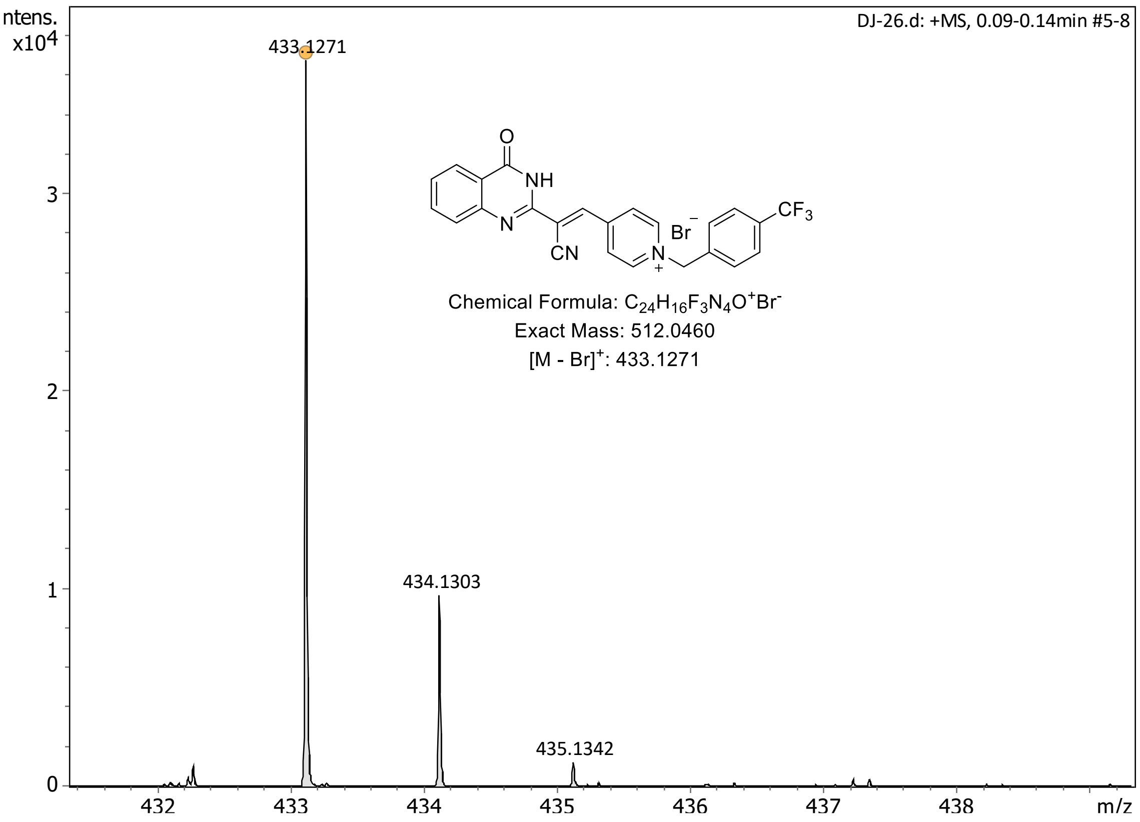

12.25 Spectra of compound **13g**

^{1}H NMR spectrum

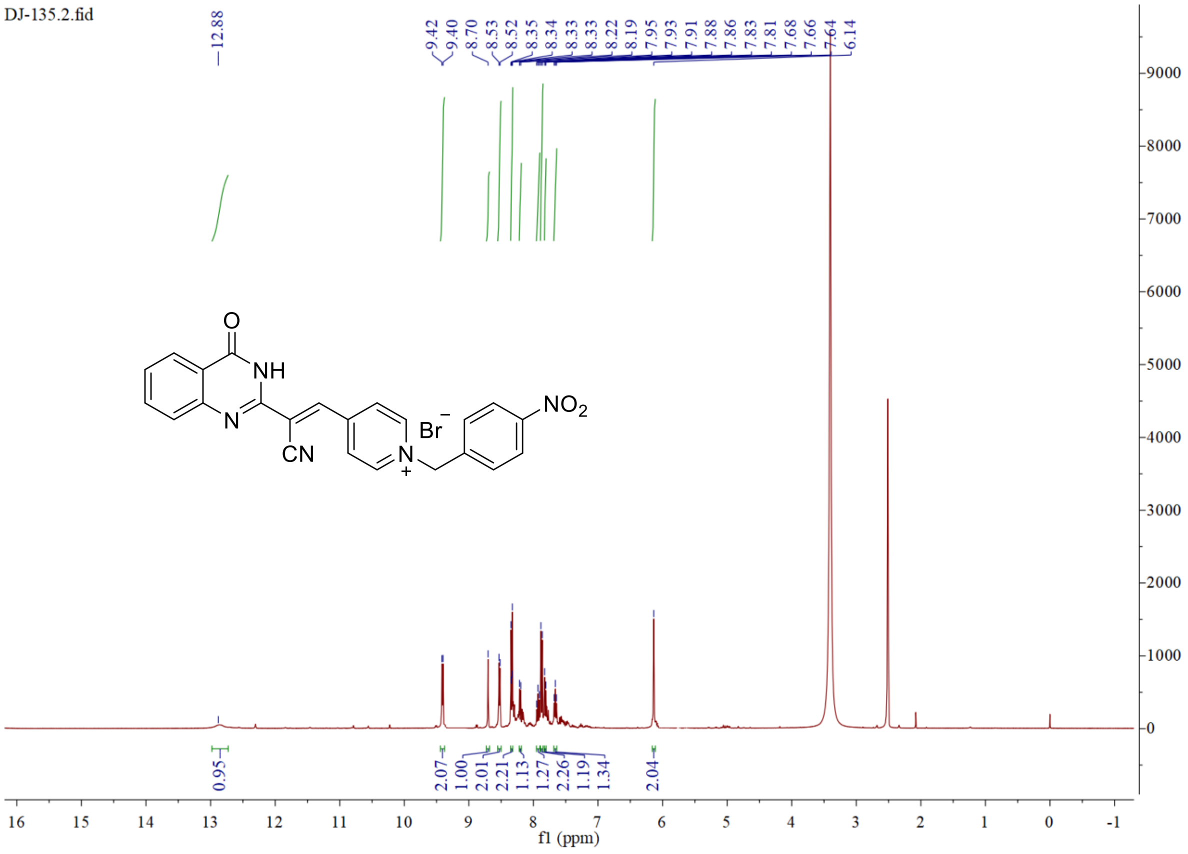

^{13}C NMR spectrum

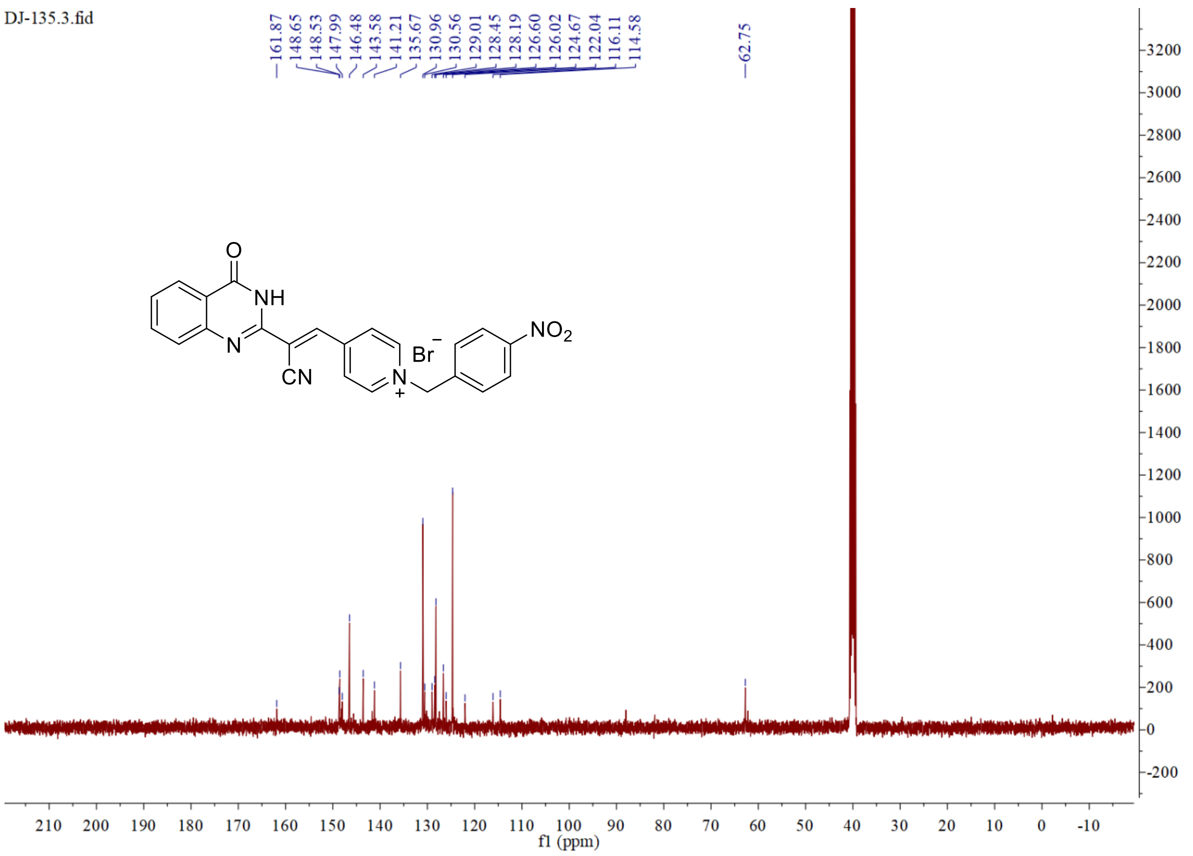

HRMS spectrum

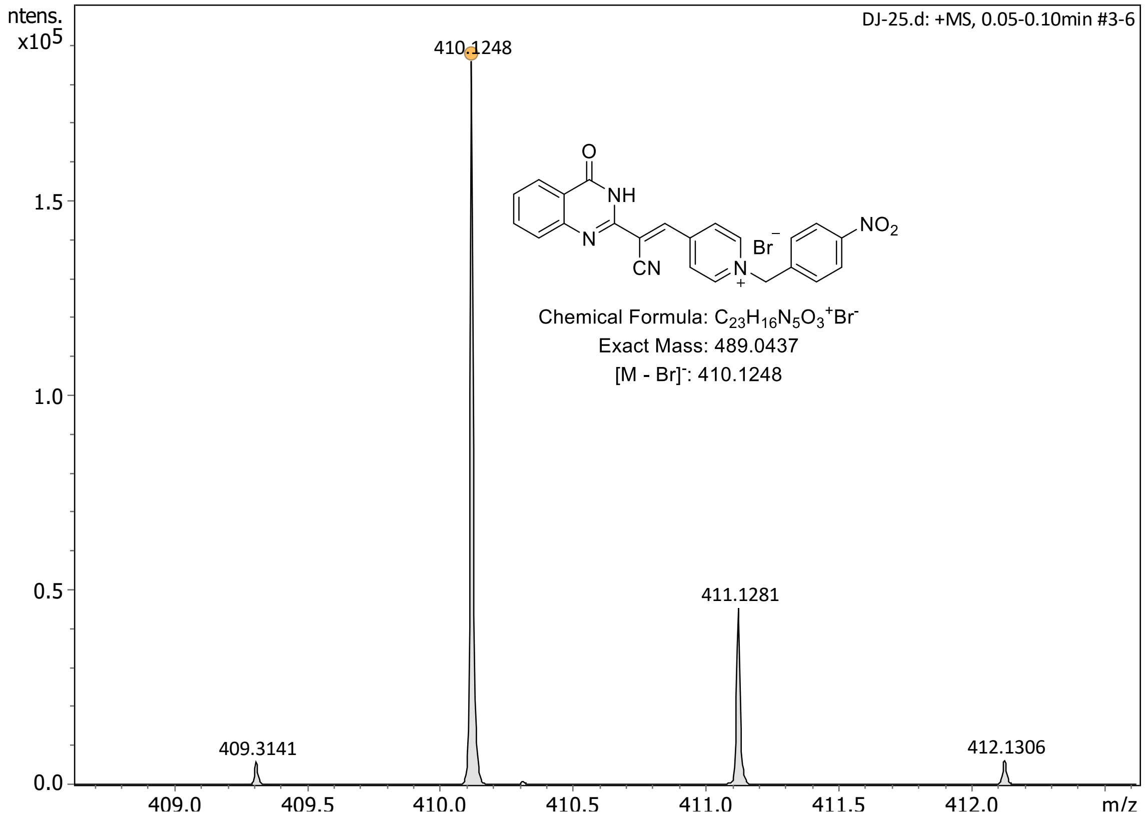

12.26 Spectra of compound **13h**

^{1}H NMR spectrum

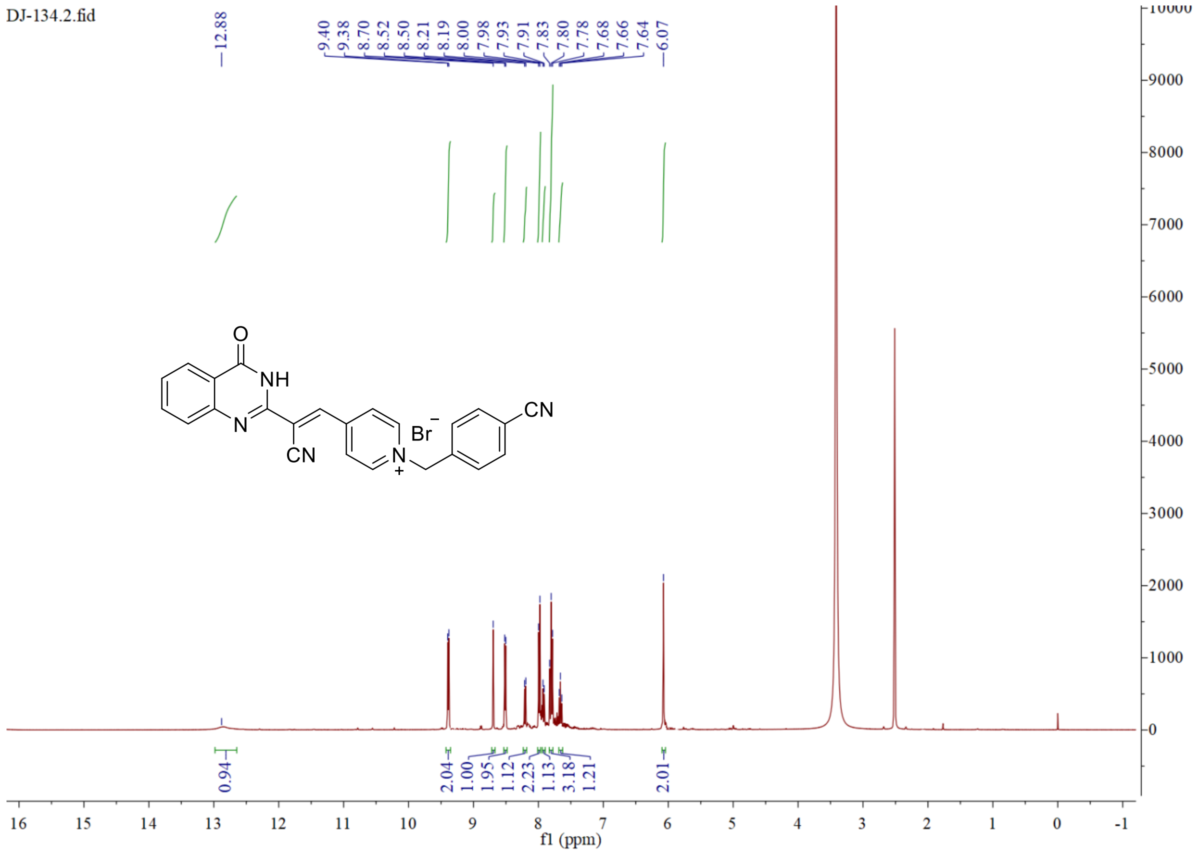

^{13}C NMR spectrum

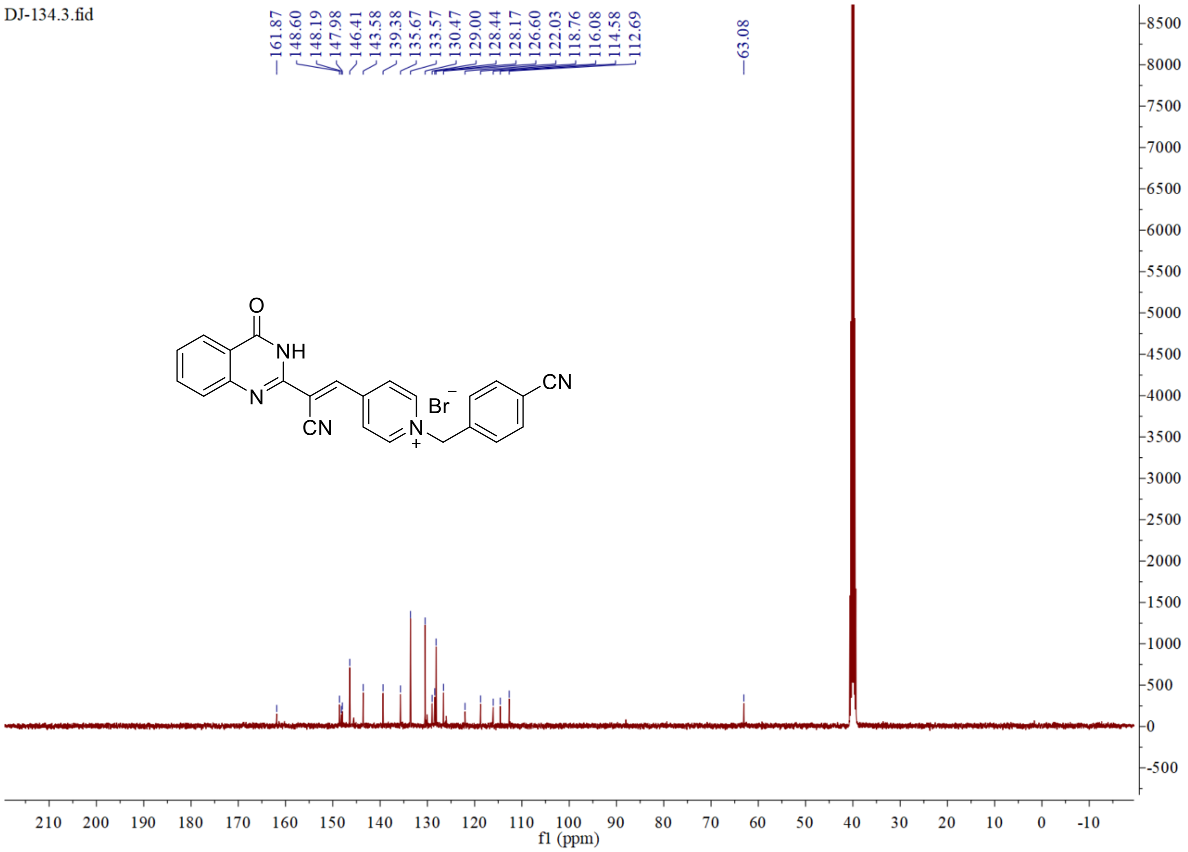

HRMS spectrum

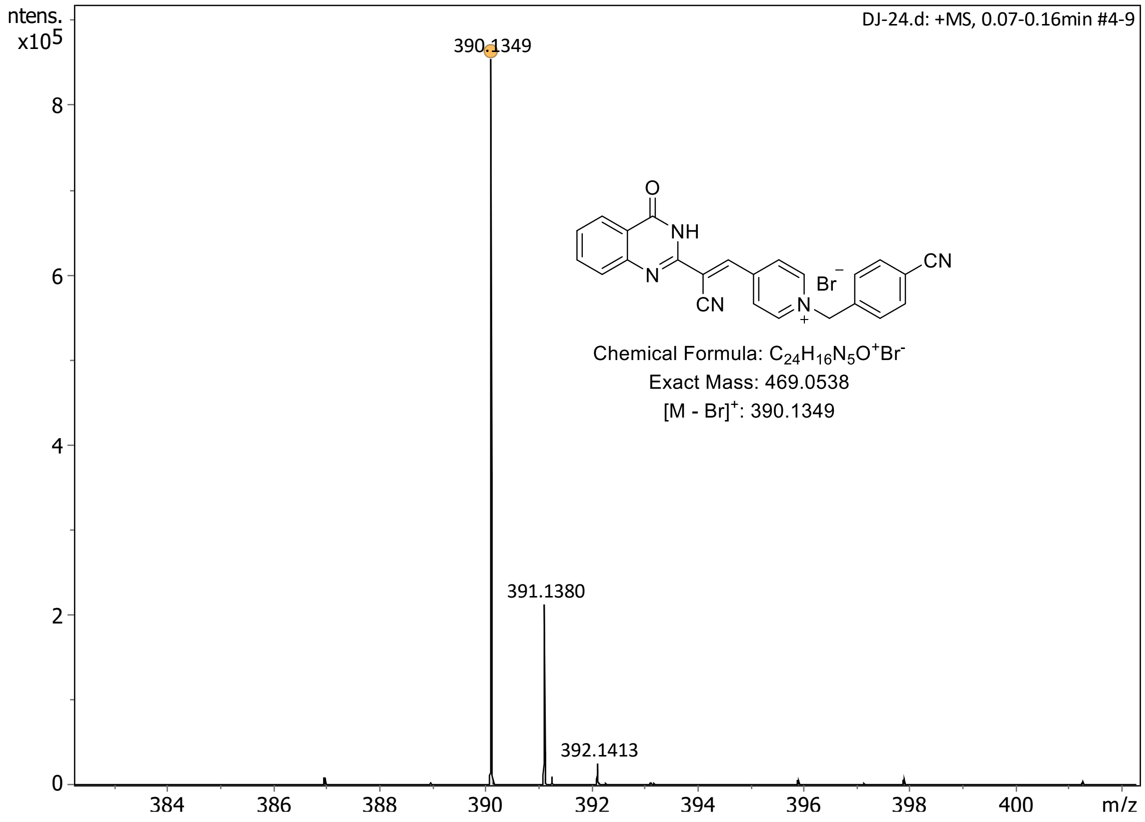

12.27 Spectra of compound **13i**

^{1}H NMR spectrum

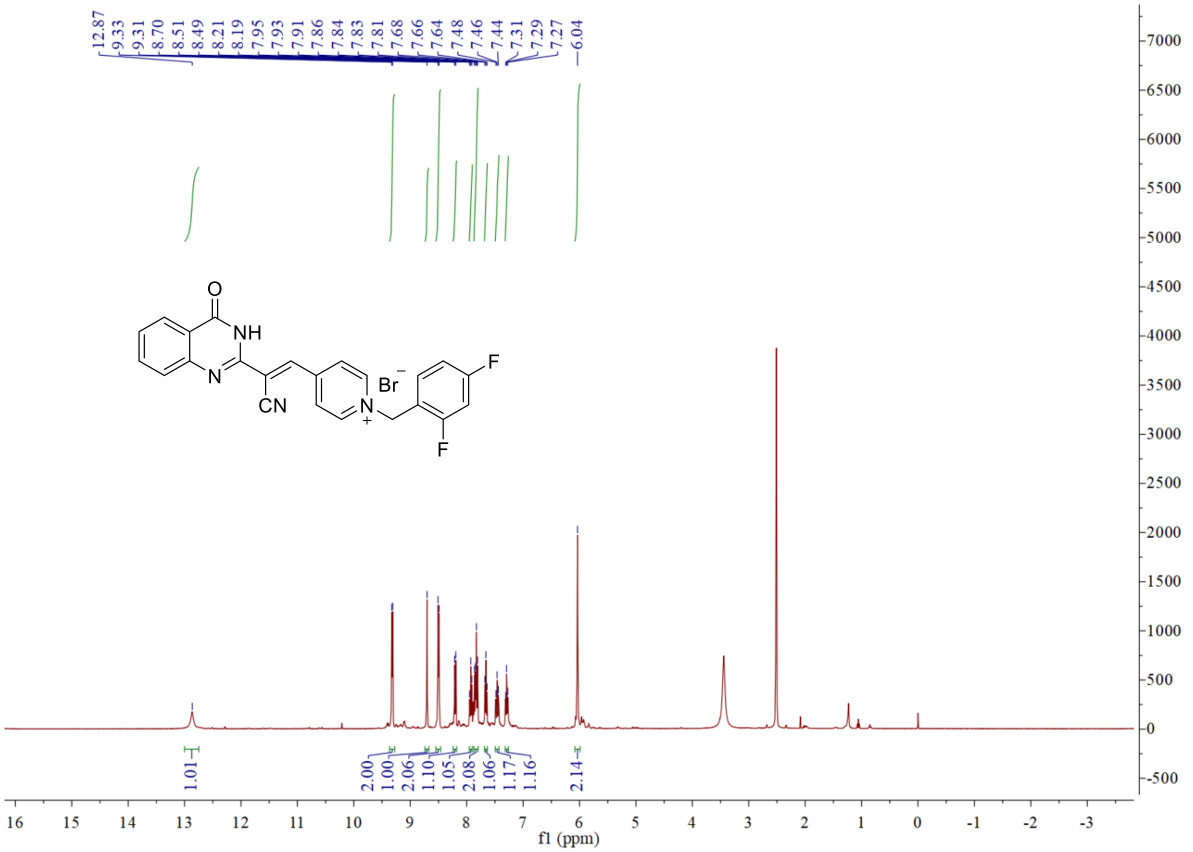

^{13}C NMR spectrum

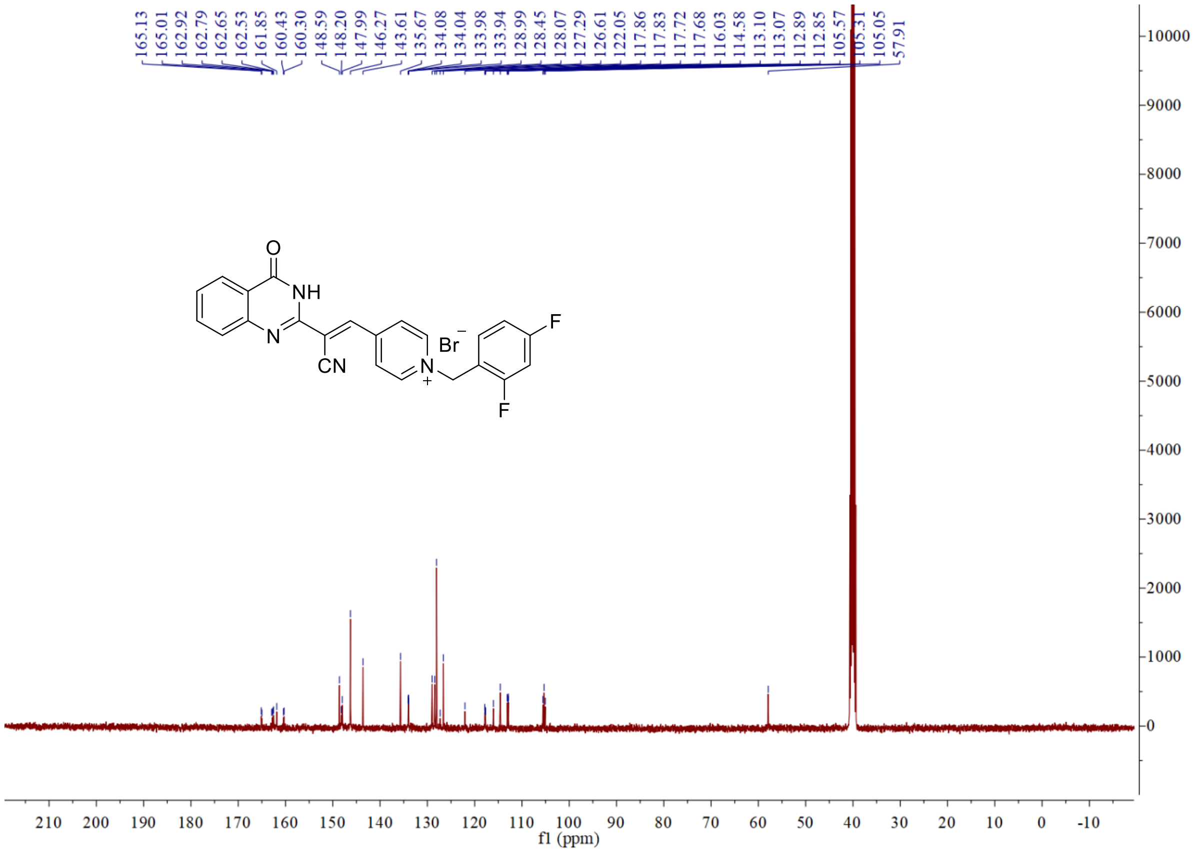

HRMS spectrum

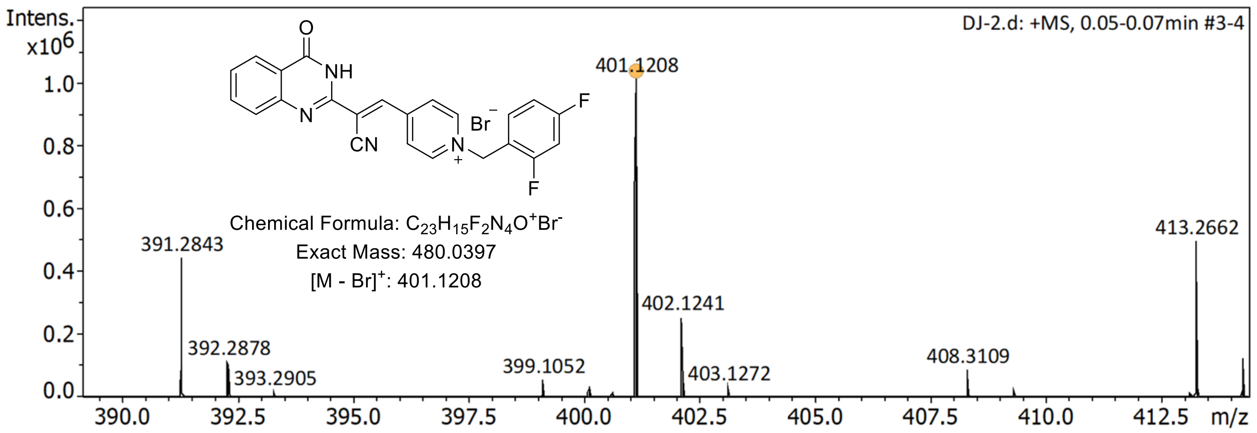

Meas. m/z	#	Ion Formula	m/z	err [ppm]	mSigma	# mSigma	Score	rdb	e¡¥ Conf	N-Rule
401.1208	1	C23H15F2N4O	401.1208	0.2	9.0	1	100.00	18.0	even	ok

12.28 Spectra of compound **19a**

^{1}H NMR spectrum

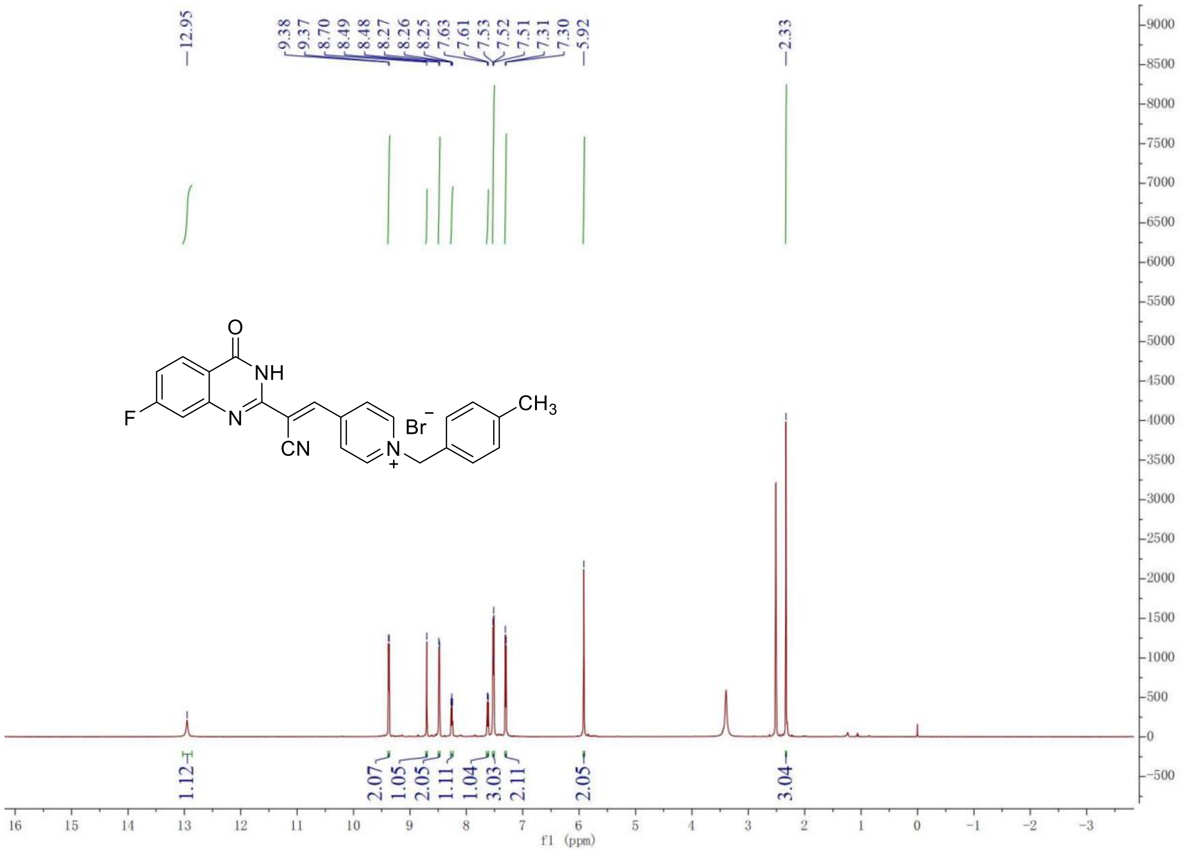

^{13}C NMR spectrum

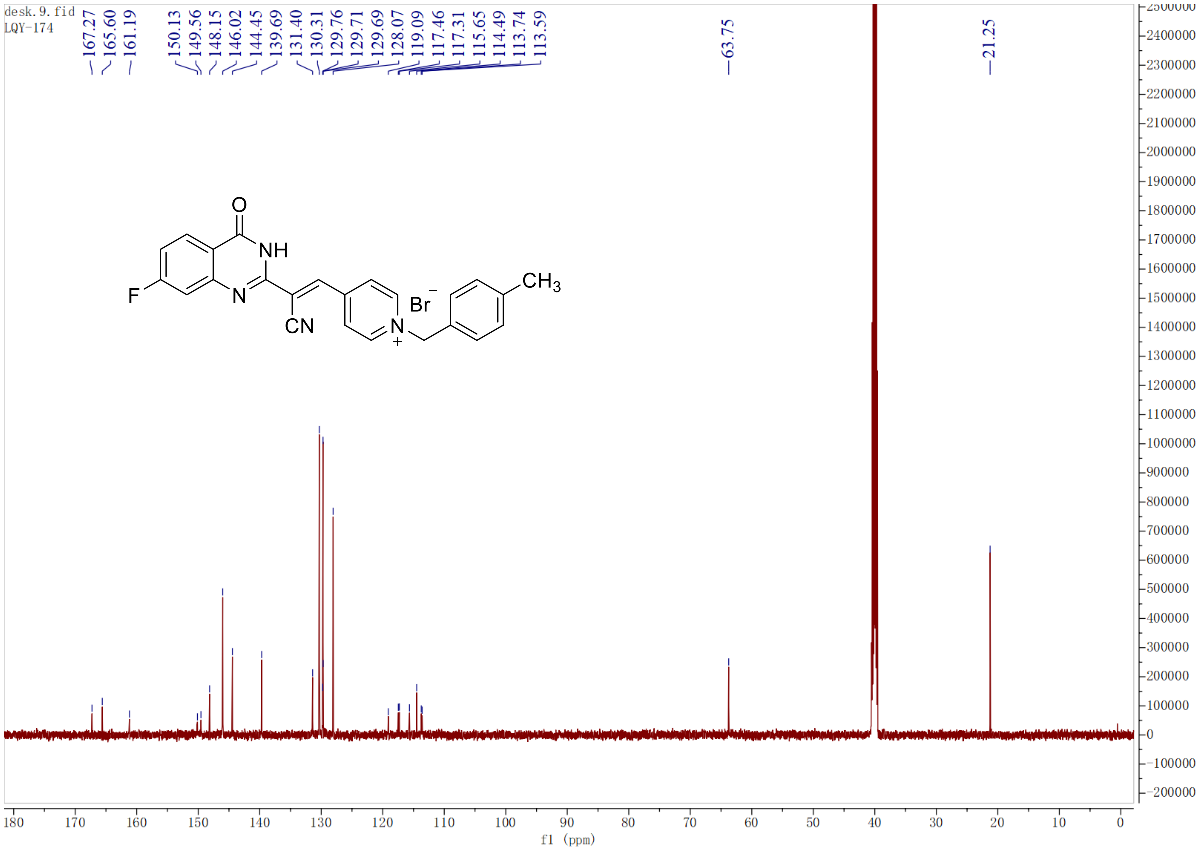

DEPT spectrum

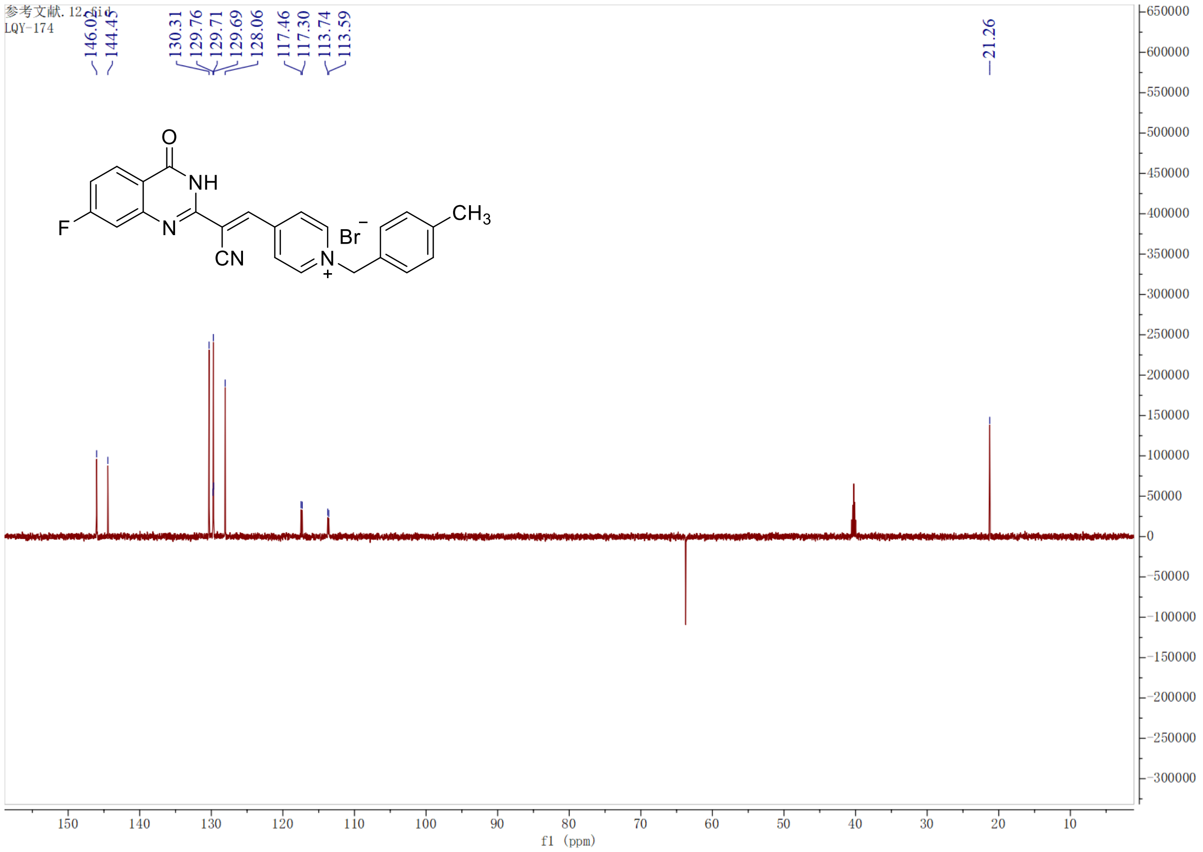

HMQC spectrum

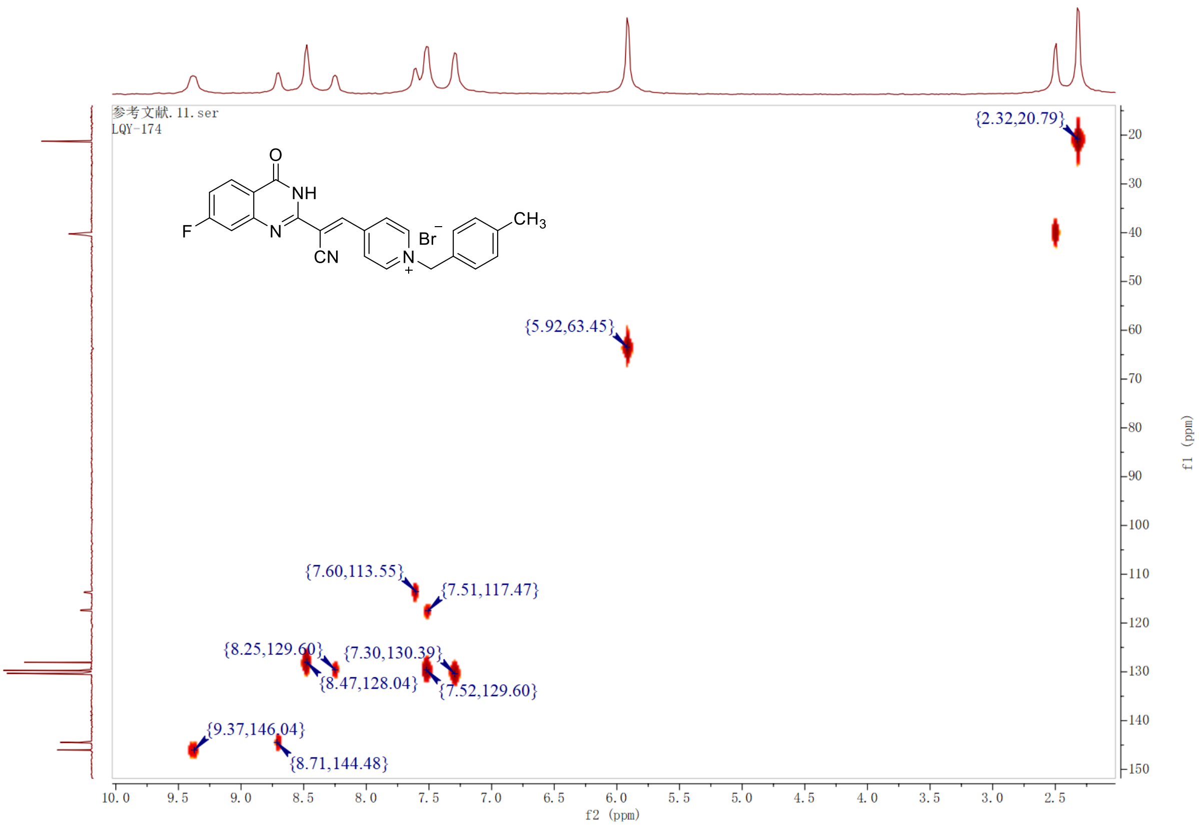

HRMS spectrum

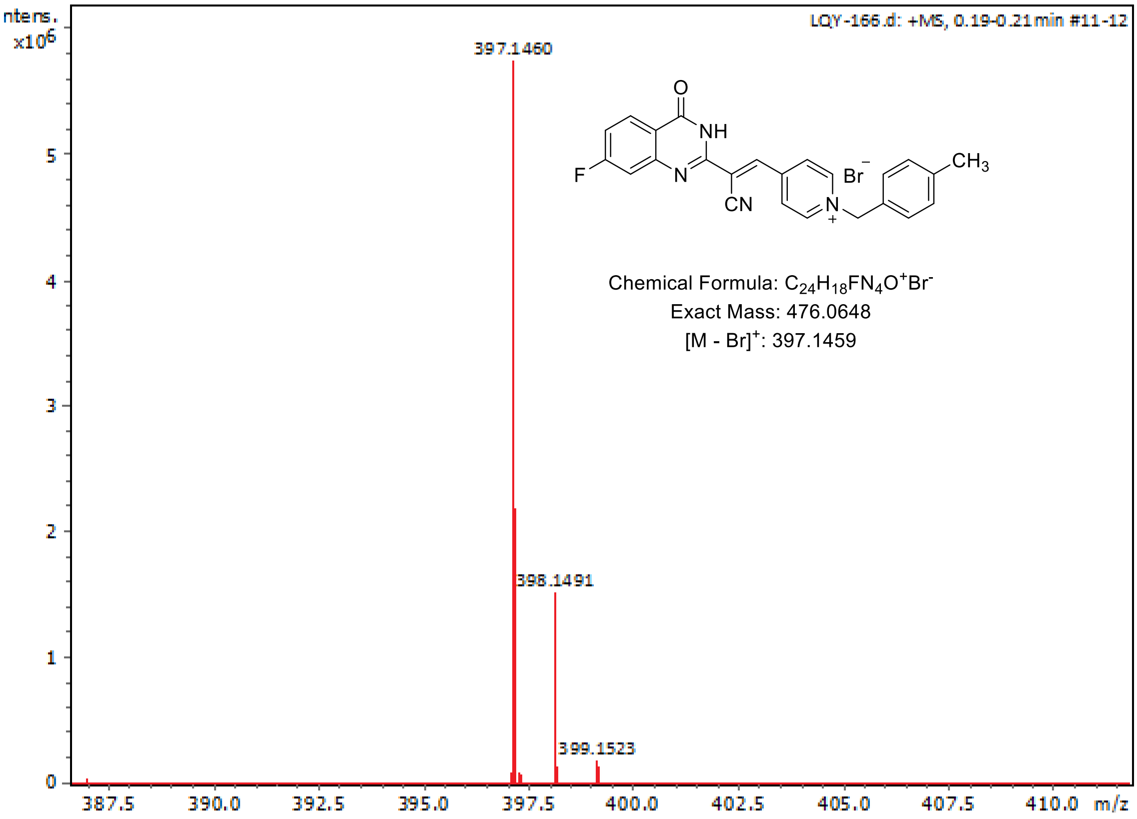

12.29 Spectra of compound **19b**

^{1}H NMR spectrum

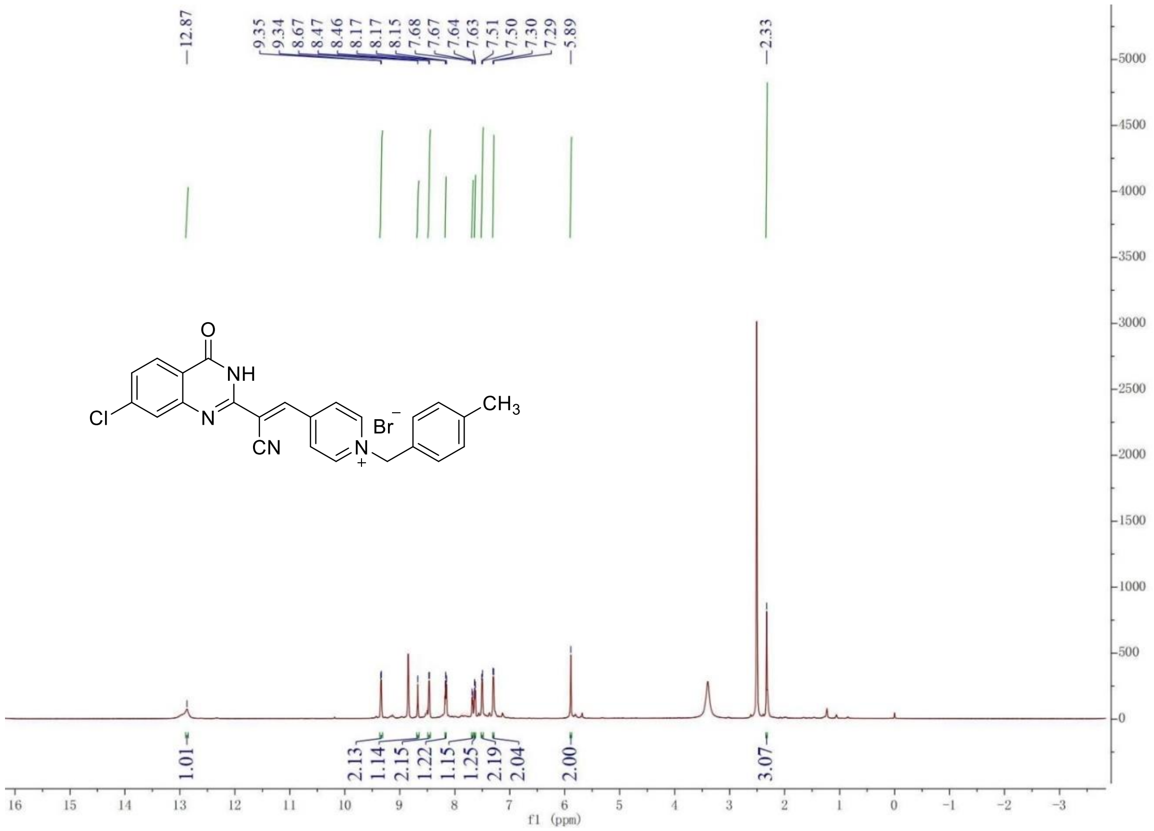

12.30 Spectra of compound **19c**

^{1}H NMR spectrum

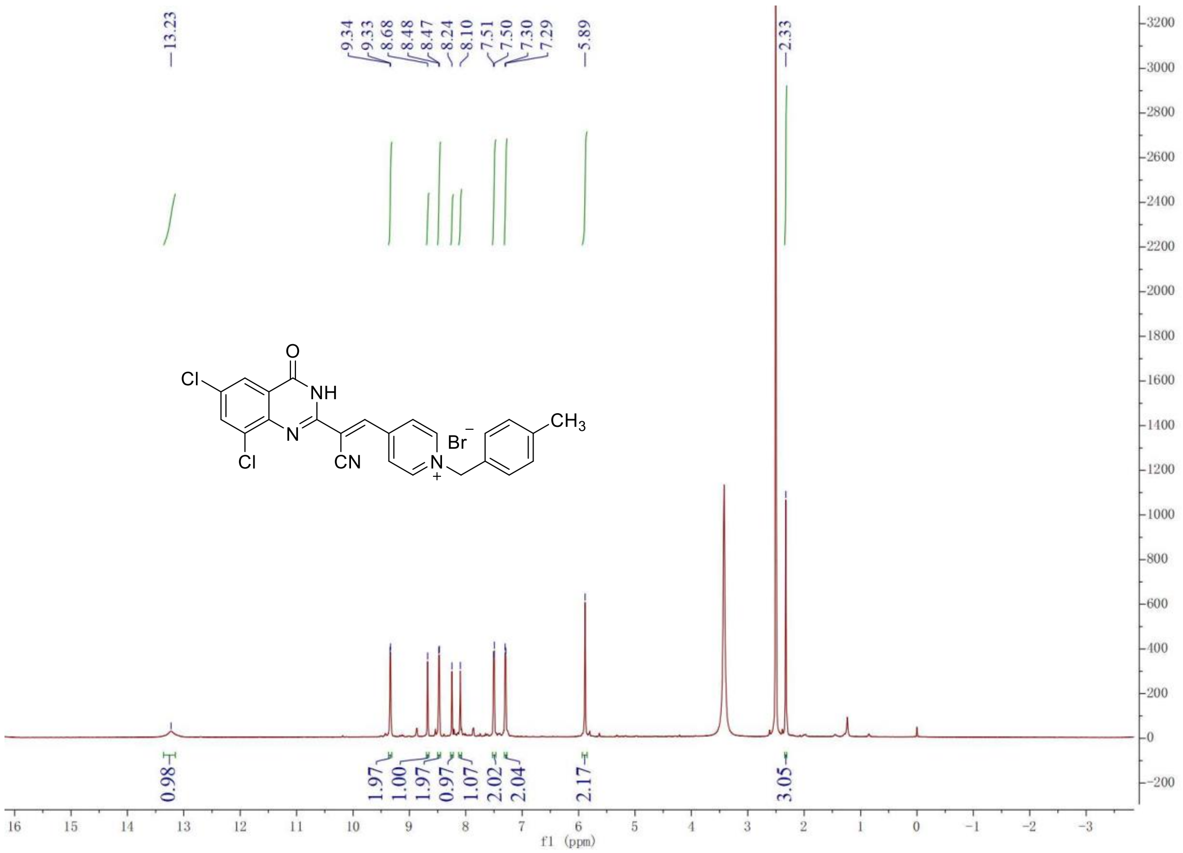

12.31 Spectra of compound **19d**

^{1}H NMR spectrum

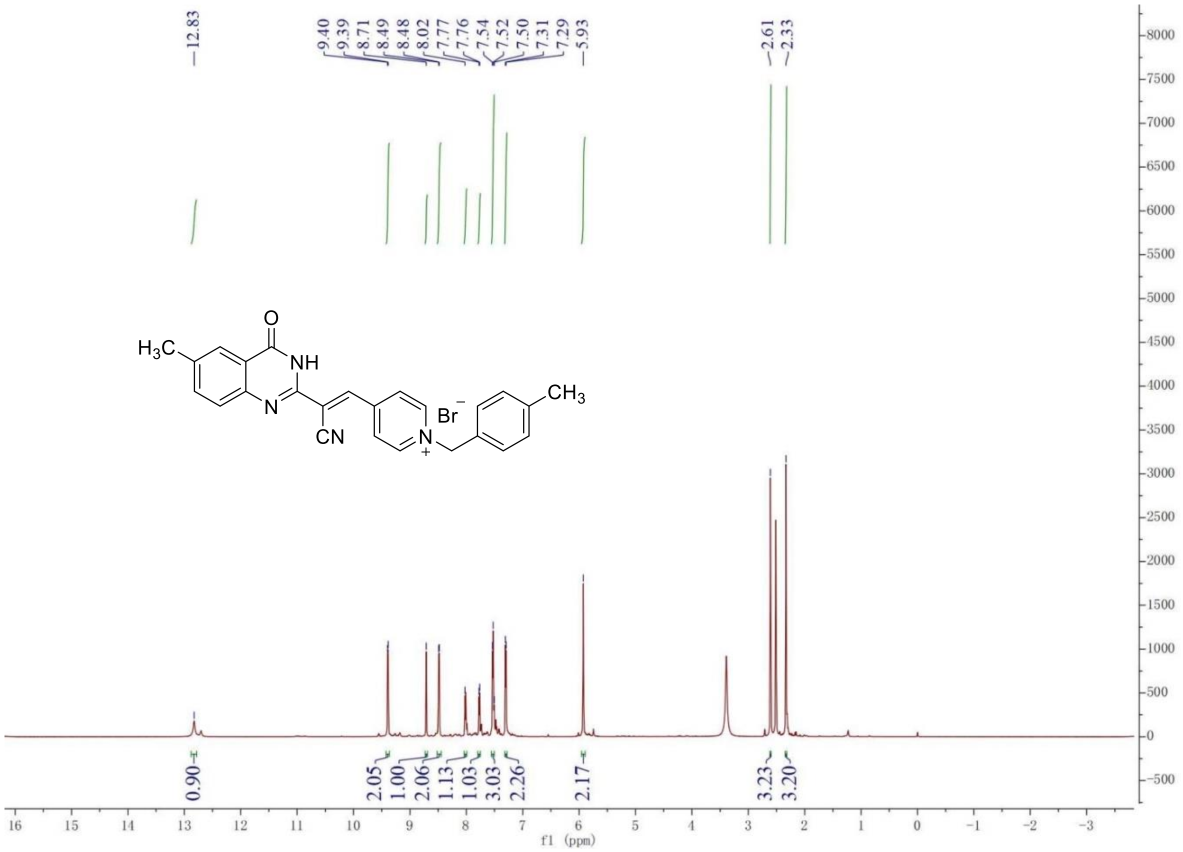

^{13}C NMR spectrum

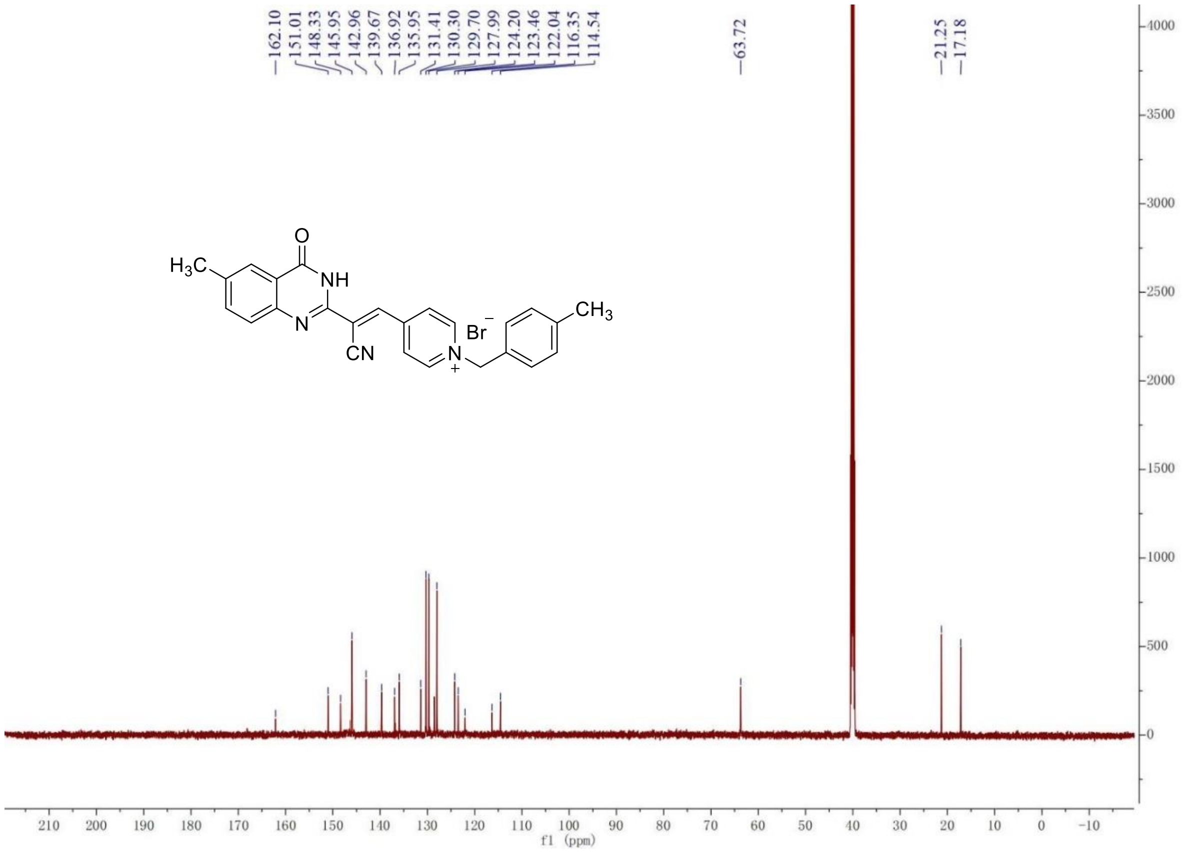

12.32 Spectra of compound **19e**

^{1}H NMR spectrum

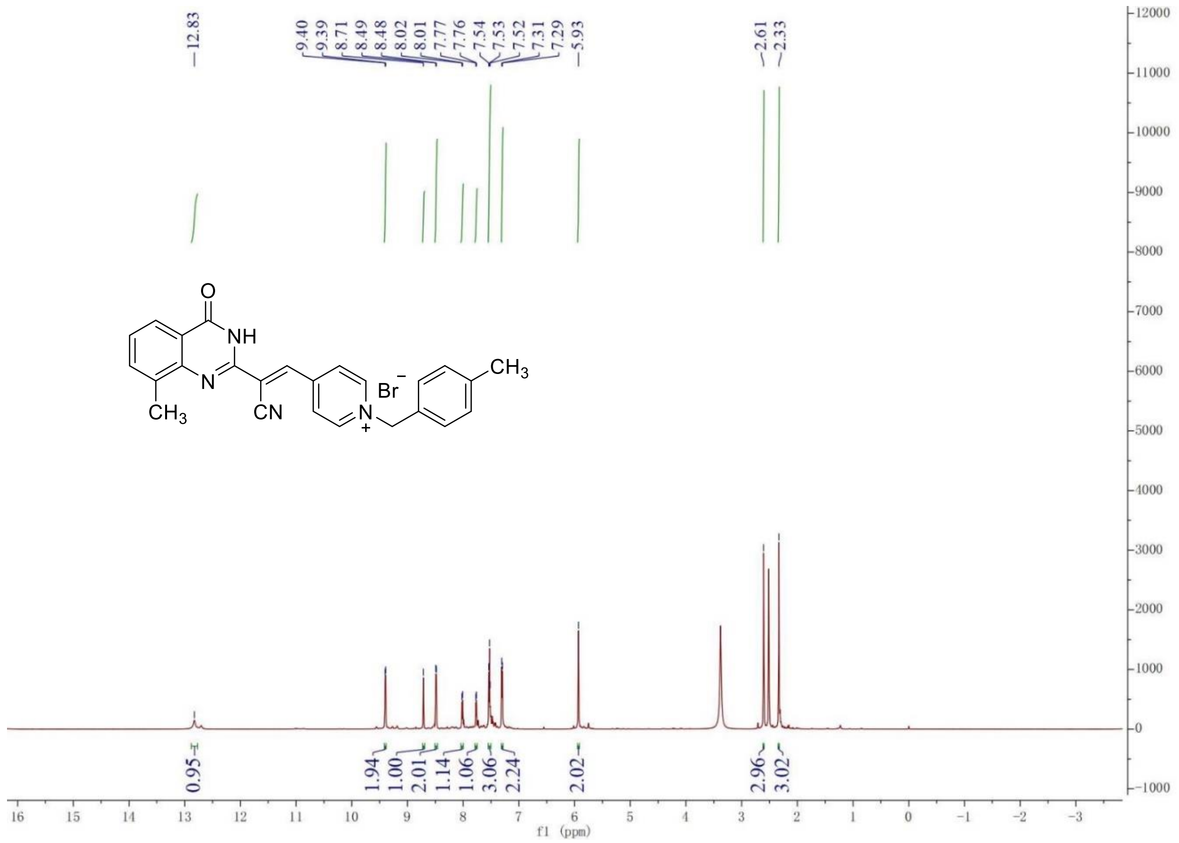

^{13}C NMR spectrum

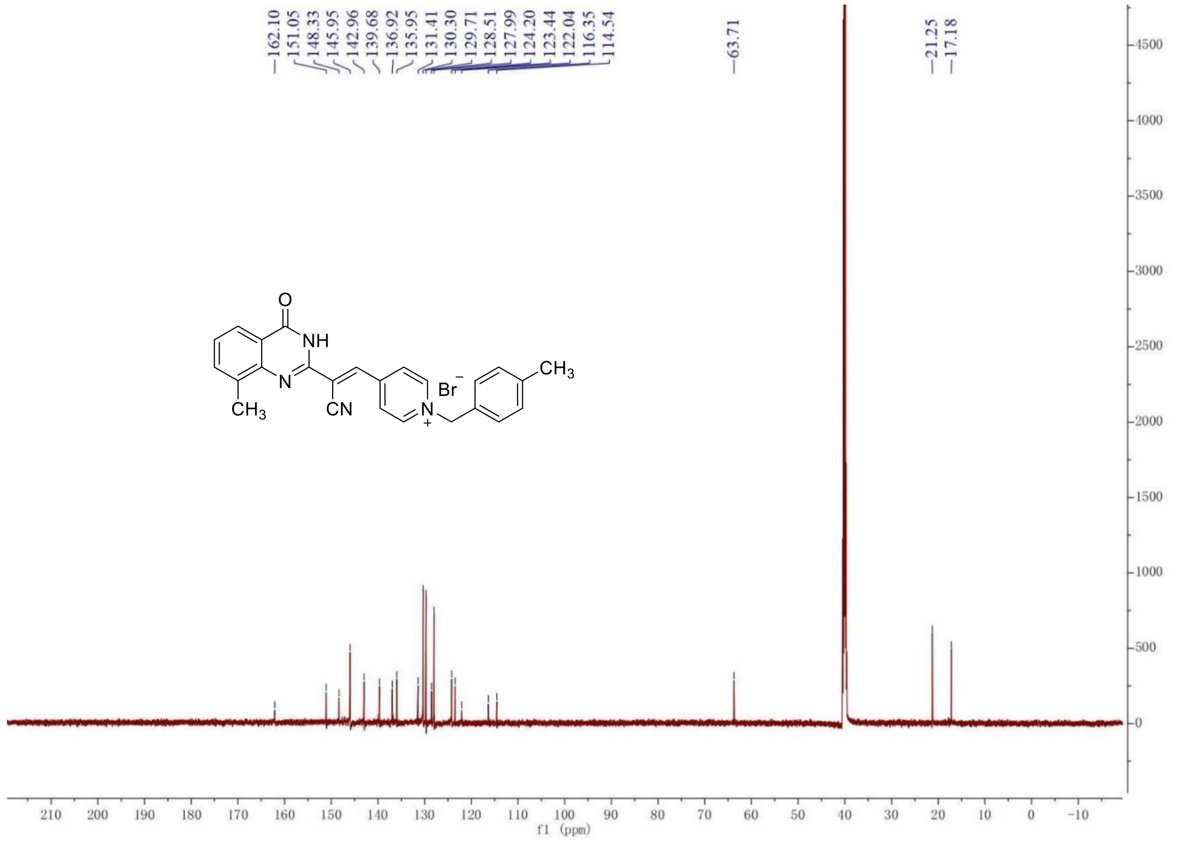

Supplement: Supplementary file 1 [file molecules-30-00243-s001.zip › molecules-3372373-supplementary.pdf]
